# Vaccination Coverage Against Coronavirus Disease 2019 in People Living on Quilombos in Brazil and Its Association With the Human Development Index and the Quality of the Health System

**DOI:** 10.1002/jmv.70533

**Published:** 2025-08-05

**Authors:** Patrícia Teixeira Costa, Lucas Silva Mello, Luiz Felipe Azevedo Marques, Vinícius Santiago dos Santos, Fernando Augusto Lima Marson

**Affiliations:** ^1^ Laboratory of Molecular Biology and Genetics, Health Sciences Postgraduate Program São Francisco University Bragança Paulista São Paulo Brazil; ^2^ Laboratory of Clinical and Molecular Microbiology, Health Sciences Postgraduate Program São Francisco University Bragança Paulista São Paulo Brazil; ^3^ LunGuardian Research Group – Epidemiology of Respiratory and Infectious Diseases, Health Sciences Postgraduate Program São Francisco University Bragança Paulista São Paulo Brazil

**Keywords:** epidemiology, pandemic, public health, quilombo, SARS‐CoV‐2, vaccine, vulnerable population

## Abstract

Since the slavery period in Brazil, the Quilombola population has faced problems related to social vulnerability and poor health. The coronavirus disease (COVID)‐19 pandemic has made this social inequality more evident, and the postpandemic scenario has proved even more challenging with the difficult access to basic living conditions, including low vaccination coverage. In view of this, this study aims to present the epidemiological profile of vaccination against COVID‐19 in the population living in quilombos in Brazil. An observational study was carried out using information from the National Health Data Network provided by the Brazilian Ministry of Health. Vaccination coverage was correlated with the human development index (HDI) and health quality indicators. In the Quilombola population, a total of 1 848 494 doses of vaccines were administered, mostly related to the first dose (45.43%) and the second or single‐dose vaccine vials (42.17%), representing a vaccination coverage of 48.43% and 49.41%, respectively; in addition, a total of 159.26 doses were administered per 100 inhabitants. There was an imbalance in the vaccination coverage rate and the number of doses administered in relation to the macroregions of Brazil and the Federative Units. In terms of doses administered by age group, the highest concentration was observed among individuals aged 20–59, with over 100 000 doses administered within each group. In the Spearman correlation, the following coefficients were significant: (a) first dose with municipal health spending [CC = −0.57]; (b) second dose or single‐dose vaccine vial with HDI [CC = 0.58], HDI–income [CC = 0.58], HDI–education [CC = 0.65], and municipal health spending [CC = −0.61]; and (c) the total number of doses with HDI [CC = 0.58], HDI–income [CC = 0.56], HDI–education [CC = 0.67], and municipal health spending [CC = −0.55]. The epidemiological profile of vaccination against COVID‐19 in the Quilombola population in Brazil was associated with a wide variability in relation to macroregions and Federative Units, with few Federative Units vaccinating more than 50% of Quilombola individuals. Among the markers evaluated, the best HDI and the best quality of health services were associated with better vaccination coverage for the first dose, second dose, single‐dose vaccine vial, and total number of doses administered in the Quilombola population, demonstrating that there is a relation between social and economic characteristics and the management of vaccines with a potential influence on the outcomes associated with the COVID‐19 pandemic.

## 1 Introduction

During the slavery period, Brazil received several ships with millions of slaves from the most diverse parts of Africa, distributed throughout Brazilian territory, but mostly concentrated in the North and Northeast regions [1]. After centuries of oppression, these people banded together in smaller groups and spread out through the forests in a form of resistance, thus giving rise to the quilombos [2, 3]. Thus, these peoples are characterized by having Black ancestry, specific territorial relations, and their own religious, sociocultural, and economic customs [4, 5]. Currently, there are 2958 quilombos in Brazil [4, 6] but there is a divergence about this number [7, 8] since these people were only included in Brazil's demographic census in 2020 [7].

Among the countless marginalization strategies used to make Quilombolas invisible, one of the main ones was to deny them the right to citizenship. Only in 1850 were they considered freed, but still without the title of citizens of Brazil [9]. After the abolition of slavery in 1888, an arduous process of winning basic rights began [6], which were only acquired after the 1988 Federal Constitution was established [1, 3]. Subsequently, they came to be known as quilombo remaining communities [10, 11, 12]. Even after these advances, Quilombolas still face numerous difficulties, including access to health, education, and quality infrastructure, as well as being in geographically remote areas [10, 11, 13]. Such precarious conditions are linked to the ethnic–racial discrimination suffered by these people for generations, which significantly contributes to their vulnerability, especially in the area of health [5, 6, 14].

The recent pandemic declared by the World Health Organization in 2020, caused by the severe acute respiratory syndrome coronavirus 2 (SARS‐CoV‐2), has triggered numerous existing social inequalities [1, 15, 16]. In Brazil, this process has been inflated by state inefficiency in managing the pandemic, whether by loosening laws protecting Indigenous and Quilombola lands [1, 17, 18, 19, 20] which were based on encouraging miners, farmers, and cattle ranchers to occupy these peoples' lands for use and exploitation [18], by discrediting the effectiveness of vaccination against coronavirus disease (COVID)‐19, which brought as an effect the disorder over the correct treatment and adherence by the population to protective measures [21, 22]. Such conduct has made Brazil one of the worst countries in managing the pandemic [1, 21]. As a result, the postpandemic scenario has become even more challenging for minority groups due to their difficult access to basic living conditions [1, 4, 23, 24].

There is a problem in terms of health due to adverse socioeconomic conditions and institutionalized racism resulting from the COVID‐19 pandemic. Less than 7% of Quilombola lands are regularized by the National Institute of Colonization and Agrarian Reform, making access to vaccination and medical care difficult, as well as contributing to the underreporting of cases in this group, especially during the pandemic [1, 7, 8, 9, 25]. Studies indicate that chronic diseases such as systemic arterial hypertension and diabetes mellitus have a high incidence among Quilombolas, reflected in the higher mortality rates from COVID‐19 in the Black population compared to the White population due to the interaction of prevalent chronic diseases with COVID‐19 as well as the lack of access to medical care [1, 4, 5, 7, 10, 13, 25, 26]. Moreover, the epidemiological profile showed a higher percentage of COVID‐19 among females, adults, with low levels of schooling and family farming workers [1, 4, 8, 10, 27].

About vaccination, the situation is equally worrying, as the World Health Organization encourages vaccination coverage of at least 90% of the general population, however, vaccination coverage among Quilombolas was only 52.8% [28]. These rates have been exacerbated by the pandemic due to social vulnerability, lack of regularization of territories, and insufficient inclusion in state vaccination plans [29]. Only 12 of the 23 Brazilian States with an available vaccination plan included Indigenous and Quilombola populations as priority groups in their COVID‐19 vaccination plans, initially resulting in a national vaccination rate of only 0.62% [23, 25, 28].

To improve vaccination strategies and better reach vulnerable populations such as Quilombolas, international evidence suggests that expanding vaccination sites beyond traditional health facilities to include pharmacies, schools, and community centers can be highly effective. For instance, pharmacy‐based vaccine delivery in regions of Italy demonstrated increased vaccination coverage and improved access, particularly for adults, elderly, and vulnerable groups [30]. Similarly, regional strategies in Italy employing alternative vaccination providers such as family pediatricians and hospitals contributed to significant improvements in vaccine uptake across diverse populations [31]. Furthermore, the COVID‐19 vaccination campaign globally has highlighted the importance of resilient public health systems, community engagement, and tailored communication strategies to overcome logistical and sociocultural barriers, emphasizing innovative partnerships and technology integration for equitable vaccine distribution [32].

Additionally, nationwide, 1 176 173 doses of COVID‐19 vaccines were allocated to declared Indigenous people and Quilombolas, with the largest contingent of vaccinated Quilombolas in the Northeast (40.6%), highlighting the prioritization of these groups in this region. However, 15.76% of CoronaVac regimens and 99.58% of AstraZeneca regimens were not complete for this priority group, indicating variations in vaccine availability and acceptance. Temporal analysis revealed different patterns of vaccination, with high clusters of initial doses of CoronaVac in the first few weeks and later vaccination, especially for AstraZeneca, among Quilombolas [25].

With this scenario, a set of prevention and health promotion actions is needed to protect the historically marginalized Quilombola group, as the cultural impact of the loss of a Quilombola represents a significant reduction for the collective memory and identity of these communities [5]. Considering this context, the objective of this study was to present the epidemiological profile of vaccination against COVID‐19 in the Quilombola population in Brazil and to associate this profile with the human development index (HDI) and indicators of quality of health services offered to the population.

## 2 Methods

### 2.1 Profile of Vaccination Against COVID‐19 in Quilombolas in Brazil

This observational epidemiological study used data from a public platform containing information on COVID‐19 vaccines available in Brazil (https://infoms.saude.gov.br/). The platform was developed by the Department of Monitoring, Evaluation, and Dissemination of Strategic Health Information in partnership with the Department of Immunization and Immunopreventable Diseases [33]. The authors obtained and compiled data covering the COVID‐19 pandemic period, updated until July 31, 2024.

The platform's data, sourced exclusively from the National Health Data Network and updated daily, are categorized into 11 filters: (i) region of the country, (ii) Federation Unit (States and Federal District), (iii) health macroregion (Central‐West, North, Northeast, South, and Southeast), (iv) health region, (v) municipality, (vi) year of vaccination, (vii) month of vaccination, (viii) date of vaccination, (ix) vaccine administered, (x) number of doses, and (xi) sex. The system is organized into four central themes: (i) monovalent vaccine, (ii) bivalent vaccine, (iii) Indigenous population served by the Indigenous Health Subsystem of Brazil's Unified Health System, and (iv) Quilombola population [33]. Notably, the Quilombola population includes only three filters: region of the country, Federation Unit, and municipality, making the full 11‐filter system applicable only to monovalent and bivalent vaccination data.

In this context, through the COVID‐19 vaccine information portal, data on Brazil's Quilombola population in relation to vaccination was quantified and categorized by total data and fractional data. The total data were stratified by type of dose (first dose, second dose and single‐dose vaccine vial, additional dose, booster dose, and 2nd booster dose), total number of doses administered, Quilombola population (number of inhabitants), and total number of vaccines administered and approved by Brazil's National Health Surveillance Agency [(a) Butantan–Sinovac—Coronavac, inactivated virus vaccine; (b) Fiocruz/AstraZeneca—ChAdOx‐1 nCov‐19—chimpanzee adenovirus vectored vaccine expressing the SARS‐CoV‐2 Spike protein; (c) Janssen—Ad26.COV.2‐S based on a nonreplicating recombinant vector of adenovirus serotype 26 (Ad26), which encodes the complete and stabilized spike (S) protein of SARS‐CoV‐2; and (d) Pfizer–Biontech—BNT162b2, mRNA vaccine that encodes for the complete S protein and not just the receptor binding domain].

The fractional data were analyzed based on the distribution of doses across Brazil's territory (doses administered by region and by Federation Unit—States and Federal District), in relation to population size, and stratified by age groups (3–4 years, 5–11 years, 12–17 years, 18–19 years, 20–24 years, 25–29 years, 30–34 years, 35–39 years, 40–44 years, 45–49 years, 50–54 years, 55–59 years, 60–64 years, 65–69 years, 70–74 years, 75–79 years, and 80 years and over) within the Quilombola population.

### 2.2 Population Census

The 2022 census of the Quilombola population was obtained from the Brazilian Institute of Geography and Statistics (IBGE of the Portuguese *Instituto Brasileiro de Geografia e Estatística*), the official institution responsible for producing statistics data in Brazil and a key provider of information for both civil and governmental use [34]. This census provided the number of individuals self‐identified as Quilombolas by region and Federation Units. These data were used to validate the information collected from the Brazilian Ministry of Health's COVID‐19 Vaccine Information Portal [33].

### 2.3 Human Development Index

The HDI characterizes long‐term progress in three basic dimensions of human development: income, education, and life expectancy. As such, this index is a general and synthetic measure that, despite broadening the perspective on human development, does not cover or exhaust all aspects of development. In the study, the values of the general development index (average of the three dimensions) and its dimensions (income, education, and life expectancy) were obtained according to the Federation Units of Brazil.

The HDI values were calculated by the Atlas of Human Development in Brazil (United Nations Development Program for Brazil, Institute for Applied Economic Research, and João Pinheiro Foundation, 2022) using the information provided by the IBGE and administrative records, as specified in the system's metadata [35]. The following HDI classification ranges were considered: (very low) 0–0.499, (low) 0.500–0.599, (moderate) 0.600–0.699, (high) 0.700–0.799, and (very high) 0.800–1.000.

### 2.4 Health Quality Indicators of Brazil's Federation Units

The quality of health services, at the Federation Unit level, was assessed using indicators obtained from the Institute for Health Policy Studies Portal. The indicators were divided into thematic blocks: (a) basic care, (b) resources, (c) mortality and morbidity, and (d) spending (municipal and state). The Institute for Health Policy Studies Portal is a tool for mapping and building comparative analyses with data from Brazil (IEPS Data Portal—iepsdata.org.br) [36].

The following indicators were considered for each thematic block evaluated: (primary care) polio vaccination coverage, primary care coverage, and percentage of live births with adequate prenatal care; (resources) number of nurses per 1000 inhabitants, number of doctors per 1000 inhabitants, number of beds available in the Unified Health System per 100 000 inhabitants, and number of beds available and not associated with the Unified Health System per 100 000 inhabitants; (mortality and morbidity) number of hospitalizations per primary care‐sensitive conditions per 100 000 inhabitants, adjusted mortality rate due to preventable causes per 100 000 inhabitants, and infant mortality rate per 1000 live births, and (municipal and state spending—use of resources linked to health) total health spending under the responsibility of the municipality, health spending using the municipality's own resources, total health spending under the responsibility of the States or Federal District, and health spending using the States or Federal District's own resources.

The thematic blocks were described in the study by the position occupied by each State or Federal District according to the indicators evaluated, that is, the first position is indicative of better indices in the indicators and, from there, up to position 27, we have a decrease in the evaluated indices (drop in the quality of health according to the indicators evaluated in the thematic block).

### 2.5 Statistical Analysis

The data are presented in absolute numbers for the number of inhabitants and the number of doses of COVID‐19 vaccines administered in the Quilombola population. The Quilombola vaccination coverage rate was calculated using the following formula: [(Number of inhabitants/Number of vaccine doses administered) * 100]. Thus, the index shows the number of doses per 100 inhabitants, representing the vaccination coverage (%) of the population served in relation to the first and second (or single dose) doses of COVID‐19 vaccines. The HDI is presented by the absolute value and its categorization into very low, low, moderate, high, and very high levels. The health quality indicators are presented by block (primary care, resources, mortality and morbidity, and municipal and state spending), according to the position occupied by the States and the Federal District.

Descriptive and inferential statistics analyses were performed using the Statistical Package for the Social Sciences software [IBM SPSS Statistics for Macintosh, Version 28.0]. The normality of the numerical data was assessed in SPSS using the following techniques: (i) analysis of descriptive measures of central tendency, (ii) graphical methods (Q–Q graph and boxplot), and (iii) statistical testing methods (normality tests): Kolmogorov–Smirnov and Shapiro–Wilk.

The correlation between the markers was carried out using Spearman's correlation test. The analysis included the (i) points obtained in the calculation of the vaccination coverage index (first dose, second dose or single dose, and number of total doses administered), (ii) absolute values of the HDI [average and its three dimensions], and (iii) state health quality indicators—the position occupied by the States and the Federal District in each block evaluated (basic care, resources, mortality and morbidity, and municipal and state spending).

Spearman's correlation considered the following cut‐off points: (i) ±0.90–1.0 [very high positive–negative correlation index], (ii) ±0.70–0.89 [high positive–negative correlation index], (iii) ±0.40–0.69 [moderate positive–negative correlation index], (iv) ±0.10–0.39 [low positive–negative correlation index], and (v) 0.00–0.10 [insignificant positive–negative correlation index] [37]. An *α* error of 0.05 was used in the statistical analysis. Furthermore, the correlation values are presented by the correlation coefficient (CC) and its respective 95% confidence interval (95% CI).

The data were prepared in the form of tables and informative figures. The figures presented in the article were constructed in GraphPad Prism version 10.2.3 for Mac, GraphPad Software, Boston, Massachusetts, USA, www.graphpad.com.

## 3 Results

### 3.1 Epidemiological Profile of the Vaccination of the Population Living in Quilombos in Brazil

**Figure 1:**
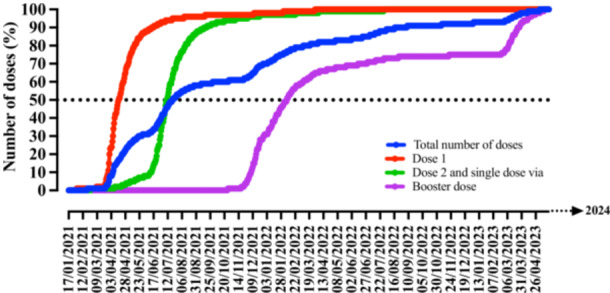
Number of doses of vaccines against coronavirus disease (COVID)‐19 administered in the Quilombola population of Brazil—temporal description. The image shows the percentage of the total number of doses administered by type of dose [dose 1 (first dose), dose 2 (second dose) and single dose, booster dose, and total number of doses]. The number of vaccine doses administered was obtained from the National Health Data Network, Brazilian Ministry of Health, updated on 07/31/2024.

**Figure 2:**
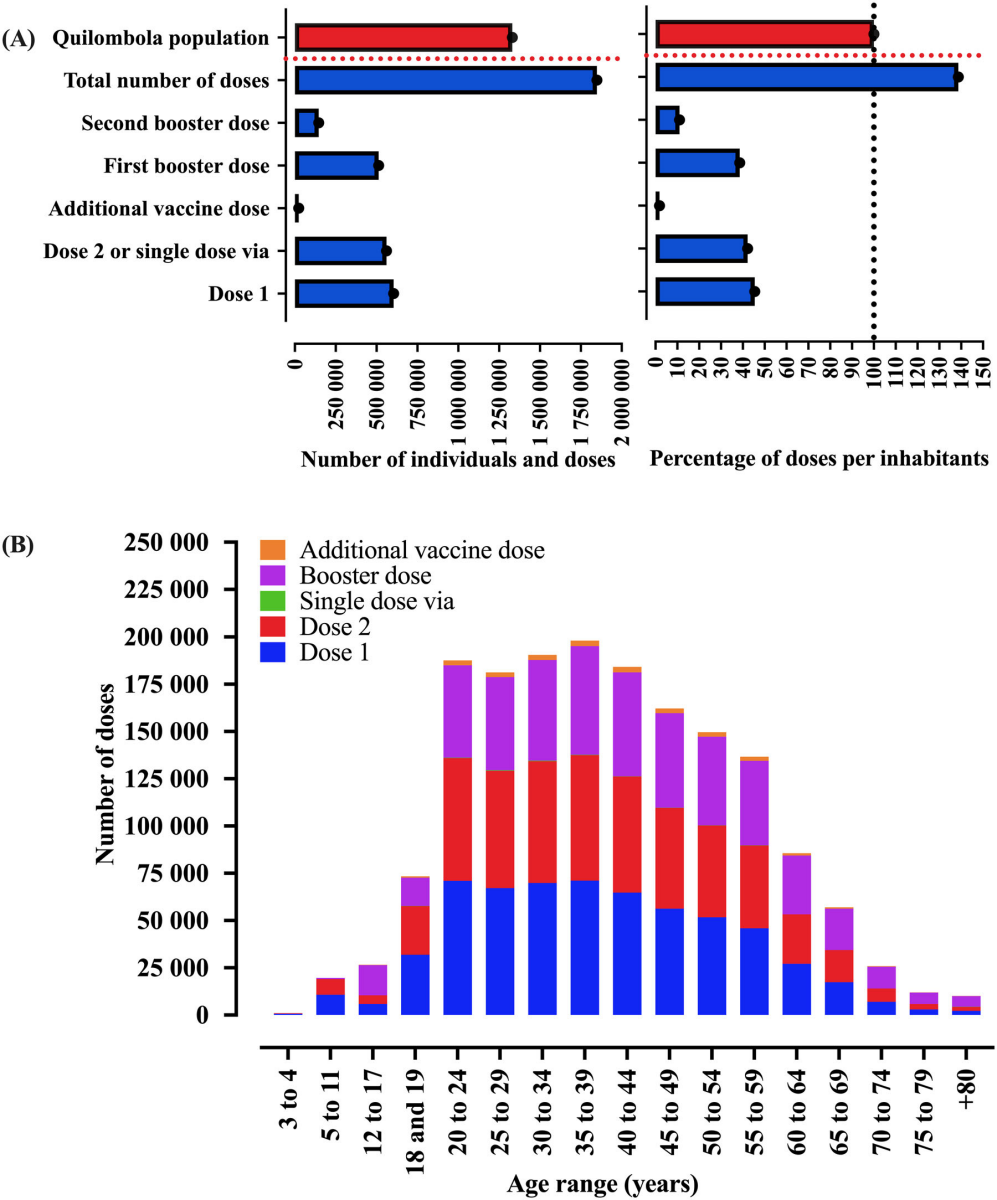
Profile of vaccination against coronavirus disease (COVID)‐19 in the Quilombola population of Brazil. (A) Overview of the distribution of dose profiles of COVID‐19 vaccines used in the Quilombola population of Brazil. (B) Overview of the distribution of dose profiles of COVID‐19 vaccines used in the Quilombola population of Brazil according to the age group of the analyzed population. The number of vaccine‐administered doses was obtained from the National Health Data Network, Brazilian Ministry of Health, updated on 07/31/2024. The age range description was made according to the platform used to obtain the data.

**Figure 3:**
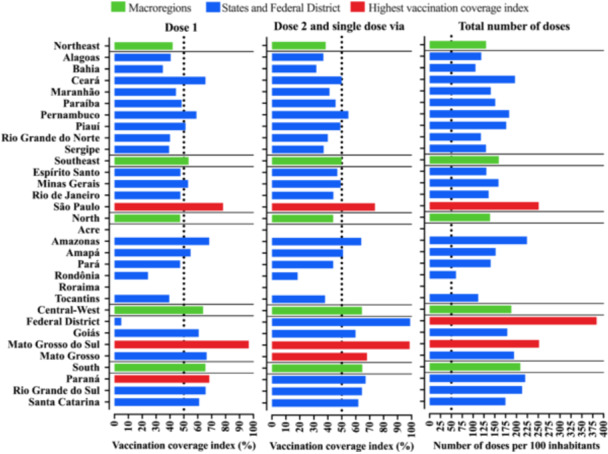
Overview of the vaccination profile (vaccination coverage rate and number of doses per 100 inhabitants) against coronavirus disease (COVID)‐19 of the Quilombola population in Brazil according to the first dose, second dose, or single dose, and total number of doses. The data are presented by macroregion (green: South, Central‐West, North, Southeast, and Northeast) and Federation Units [blue and red (best rates): States and Federal District]. The vaccination coverage rate for Quilombolas was calculated using the following formula: [(Number of inhabitants/Number of vaccine doses administered) * 100]. Thus, the index shows the number of doses per 100 inhabitants, representing the vaccination coverage (%) of the population served in relation to the first and second (or single dose) doses of COVID‐19 vaccines. The number of doses of vaccines administered was obtained from the National Health Data Network, Brazilian Ministry of Health, updated on 07/31/2024.

The distribution of the number of vaccines according to the day of vaccination and the dose profile, namely first dose, second dose or single‐dose vaccine vial, booster dose, and total doses is shown in Figure 1 and Table S1. In Brazil's Quilombola population, a total of 1 848 494 vaccine doses were administered, mostly related to the first dose (*N *= 604 329, 45.43%) and the second dose or single‐dose vaccine vial (*N* = 560 888; 42.17%) (Figure 2a). In the entire Quilombola population, vaccination coverage for the first dose was 48.43% and for the second dose or single‐dose vaccine vial was 49.41%; moreover, in relation to the total number of doses administered, a total of 159.26 doses were administered per 100 inhabitants. When describing the vaccination coverage rate and the number of doses administered in relation to the macroregions of Brazil and the Federative Units, there was an imbalance in the data described. In this context, in regard to the first dose, the best vaccination coverage rate was observed in the South region (65.61%), followed by the Central‐West (63.84%), Southeast (53.44%), North (47.28%), and Northeast (41.91%) regions. In regard to the second dose or single‐dose vaccine vial, the results were similar to the first dose, with the South region (64.76%) also showing the best vaccination coverage rate, followed by the Central‐West (64.49%), Southeast (49.89%), North (43.85%), and Northeast (38.47%) regions (Table 1 and Figure 3). Finally, in relation to the total number of doses administered, the vaccination coverage profile remained the same in relation to the rates for the first dose and the second dose or single‐dose vaccine vial [South region (208.79 doses/100 inhabitants), Central‐West region (188.37 doses/100 inhabitants), Southeast region (159.26 doses/100 inhabitants), North region (139.20 doses/100 inhabitants), and Northeast region (130.14 doses/100 inhabitants)] (Table 1 and Figure 3).

**Table 1:** Description of the distribution of doses of vaccines against coronavirus disease (COVID)‐19 in the Quilombola population of Brazil according to the Federation Units (States and Federal District) and vaccination coverage index (VCI).

Region	Dose 1	VCI^a^	Dose 2 and single dose	VCI^a^	Additional	Booster	2nd booster	Doses administered	VCI^a^	Total inhabitants^b^
Northeast	379 890	41.91	348 646	38.47	14 531	333 493	102 964	1 179 524	130.14	906 337
Alagoas	15 303	40.57	13 874	36.78	20	13 688	1938	44 823	118.82	37 724
Bahia	138 531	34.85	126 067	31.71	6705	106 742	41 466	419 511	105.54	397 502
Ceará	15 718	65.51	11 983	49.94	1185	13 225	5017	47 128	196.42	23 994
Maranhão	119 257	44.31	111 023	41.25	3544	116 104	29 376	379 304	140.92	269 168
Paraíba	8113	48.39	7638	45.56	10	7370	2213	25 344	151.17	16 765
Pernambuco	46 601	59.09	43 145	54.71	785	43 832	10 054	144 417	183.12	78 864
Piauí	16 293	51.26	15 554	48.93	212	15 701	8267	56 027	176.26	31 786
Rio Grande do Norte	8971	40.10	8944	39.98	1800	5901	791	26 407	118.04	22 371
Sergipe	11 103	39.42	10 418	36.99	270	10 930	3842	36 563	129.83	28 163
Southeast	97 497	53.44	91 005	49.89	2668	79 595	19 765	290 530	159.26	182 427
Espírito Santo	7450	47.58	7336	46.85	298	5122	276	20 482	130.80	15 659
Minas Gerais	71 729	53.01	66 550	49.18	2168	59 681	14 529	214 657	158.64	135 315
Rio de Janeiro	9705	47.46	8997	44.00	117	6644	2293	27 756	135.75	20 447
São Paulo	8613	78.26	8122	73.80	85	8148	2667	27 635	251.09	11 006
North	79 111	47.28	73 365	43.85	5163	62 536	12 716	232 891	139.20	167 311
Acre	0	0	7	0	1	5	4	17	0	0
Amazonas	1920	68.28	1800	64.01	743	955	889	6307	224.29	2812
Amapá	7067	54.81	6559	50.87	823	4000	1150	19 599	152.00	12 894
Pará	64 228	47.36	59 468	43.85	3178	53 666	9960	190 500	140.48	135 603
Rondônia	706	24.14	540	18.46	11	384	136	1777	60.75	2925
Roraima	15	0	21	0	0	10	1	47	0	0
Tocantins	5175	39.57	4970	38.01	407	3516	576	14 644	111.98	13 077
Central‐West	28 728	63.84	29 019	64.49	661	20 443	5912	84 763	188.37	44 997
Federal District	15	4.92	302	99.02	11	587	257	1172	384.26	305
Goiás	18 438	60.67	18 193	59.86	462	13 431	3785	54 309	178.70	30 391
Mato Grosso do Sul	2487	96.70	2539	98.72	33	1183	234	6476	251.79	2572
Mato Grosso	7788	66.40	7985	68.08	155	5242	1636	22 806	194.44	11 729
South	19 103	65.61	348 646	64.76	1014	16 966	4850	60 786	208.79	29 114
Paraná	4862	68.35	4772	67.09	449	4290	1290	15 663	220.20	7113
Rio Grande do Sul	11 527	65.67	11 327	64.53	523	10 812	3169	37 358	212.84	17 552
Santa Catarina	2714	61.00	2754	61.90	42	1864	391	7765	174.53	4449

^a^ The vaccination coverage index represents the number of doses of vaccines administered per 100 inhabitants. The index was calculated using the following formula: [(Number of inhabitants/Number of vaccine doses administered) * 100].

^b^ The number of inhabitants was obtained from data published in the Brazilian population census, Brazilian Institute of Geography and Statistics (IBGE of the Portuguese *Instituto Brasileiro de Geografia e Estatística*) (2022). The number of doses of vaccines administered was obtained from the National Health Data Network, Brazilian Ministry of Health, updated on 07/31/2024.

In the case of Brazilian States and Federal District, there was a wide range of vaccination coverage values for the first dose, second dose, or single‐dose vaccine vial, and the total number of doses administered per 100 inhabitants. The data are shown in Figure 3 and, additionally, in Table 1, according to the Quilombola population in Brazil as described by the IBGE. Additionally, all the indices are also presented according to the Quilombola population described by the National Health Data Network (Brazilian Ministry of Health) (Table S2). At the same time, Table S3 shows the numerical difference in the number of Quilombola inhabitants between the two databases, IBGE and the National Health Data Network.

In terms of doses administered by age group, the highest concentration was in the 20–59 age groups, where the number of doses administered per group was over 100 000 doses (Figure 2b and Supporting Material S4). In the Quilombola population, the age extremes had the lowest number of doses of vaccines administered, namely individuals aged 3–4 (*N* = 1027) and 80 or over (*N* = 10 086) (Figure 2b and Supporting Material S4). Furthermore, Figure 2b and Supporting Material S4 describe the distribution of vaccines according to dose profile—first dose, second dose or single‐dose vaccine vial, booster dose, and additional dose.

### 3.2 Profile of Vaccines Used in the Vaccination of Brazil's Quilombola Population

As for the different vaccines approved for use in Brazil, there was a predominance of the ChAdOx‐1 nCov‐19 vaccine (Fiocruz/AstraZeneca) (*N* = 1 143 429 doses; 61.86%), followed by the BNT162b2 (Pfizer‐Biontech) (*N* = 418 176 doses; 22.62%), Coronavac (Butantan‐Sinovac) (*N* = 418 176 doses; 5.76%), and Ad26.COV.2‐S (Janssen) (*N* = 106 468 doses; 2.91%) vaccines. A detailed description of the different vaccines administered according to macroregion and Federation Units in Brazil is shown in Table 2.

**Table 2:** Description of the distribution of coronavirus disease (COVID)‐19 vaccine doses administered in the Quilombola population in Brazil according to Federation Units (States and Federal District), type of immunobiological, and vaccination coverage index (VCI).

Region	Butanta–Sinovac	Fiocruz–AstraZeneca	Janssen	Pfizer–Biontech	No description	Administered doses	Total inhabitants^a^	VCI^b^
Northeast	30 895	751 472	34 832	273 726	88 599	1 179 524	906 337	130.14
Alagoas	322	29 829	227	10 537	3908	44 823	37 724	118.82
Bahia	17 744	275 384	13 462	94 045	18 876	419 511	397 502	105.54
Ceará	770	28 359	1183	12 605	4211	47 128	23 994	196.42
Maranhão	6511	239 750	7079	84 625	41 339	379 304	269 168	140.92
Paraíba	739	16 004	704	6126	1771	25 344	16 765	151.17
Pernambuco	2461	88 595	5139	37 201	11 021	144 417	78 864	183.12
Piauí	221	35 357	4210	13 577	2662	56 027	31 786	176.26
Rio Grande do Norte	458	17 660	1027	5912	1350	26 407	22 371	118.04
Sergipe	1669	20 534	1801	9098	3461	36 563	28 163	129.83
Southeast	19 064	180 191	7575	67 629	16 071	290 530	182 427	159.26
Espírito Santo	872	14 182	257	4204	967	20 482	15 659	130.80
Minas Gerais	3435	143 043	5997	49 650	12 532	214 657	135 315	158.64
Rio de Janeiro	3844	15 850	511	6822	729	27 756	20 447	135.75
São Paulo	10 913	7116	810	6953	1843	27 635	11 006	251.09
North	25 550	144 730	6574	39 914	16 123	232 891	167 311	139.20
Acre	0	9	2	5	1	17	0	0
Amazonas	109	4517	199	1249	233	6307	2812	224.29
Amapá	818	13 367	144	4948	322	19 599	12 894	152.00
Pará	24 291	115 039	5973	30 420	14 777	190 500	135 603	140.48
Rondônia	27	1217	43	399	91	1777	2925	60.75
Roraima	12	10	0	24	1	47	0	0
Tocantins	293	10 571	213	2869	698	14 644	13 077	111.98
Central‐West	27 597	30 383	3172	21 740	1871	84 763	44 997	188.37
Federal District	33	413	132	574	20	1172	305	384.26
Goiás	26 785	10 251	1387	14 733	1153	54 309	30 391	178.70
Mato Grosso do Sul	507	3817	382	1478	292	6476	2572	251.79
Mato Grosso	272	15 902	1271	4955	406	22 806	11 729	194.44
South	3362	36 653	1719	15 167	3885	60 786	29 114	208.79
Paraná	528	9963	575	3535	1062	15 663	7113	220.20
Rio Grande do Sul	2784	21 117	894	9888	2675	37 358	17 552	212.84
Santa Catarina	50	5573	250	1744	148	7765	4449	174.53

^a^ The number of inhabitants was obtained from data published in the Brazilian population census, Brazilian Institute of Geography and Statistics (IBGE of the Portuguese *Instituto Brasileiro de Geografia e Estatística*) (2022).

^b^ The vaccination coverage index represents the number of doses of vaccines administered per 100 inhabitants. The index was calculated using the following formula: [(Number of inhabitants/Number of vaccine doses administered) * 100]. The number of doses of vaccines administered was obtained from the National Health Data Network, Brazilian Ministry of Health, updated on 07/31/2024.

### 3.3 Characterization of the Human Development Index and Health Quality Indicators of the Federative Units

Table 3 shows the characterization of the markers associated with the HDI and the health quality indicators of Brazil's Federative Units. The HDI is presented directly according to its three dimensions and the quality of health is presented according to its thematic blocks, as described in Section 2.

**Table 3:** Description of the human development index (HDI) and its three dimensions and the health quality indicators (five domains) according to the Brazilian Federation Units (States and Federal District).

	HDI^a^				Health quality indicators^b^				
Region	HDI	HDI–income	HDI–education	HDI–life expectancy	Primary care	Resources	Mortality and morbidity	State expenditure	Municipal expenditure
Northeast									
Alagoas	0.684	0.630	0.679	0.748	15	23	20	20	17
Bahia	0.691	0.648	0.659	0.772	19	19	17	23	19
Ceará	0.734	0.658	0.766	0.784	5	20	11	19	13
Maranhão	0.676	0.603	0.716	0.715	23	22	27	24	23
Paraíba	0.698	0.653	0.669	0.779	6	10	14	25	14
Pernambuco	0.719	0.647	0.721	0.797	16	12	12	11	22
Piauí	0.690	0.649	0.698	0.726	10	13	26	12	8
Rio Grande do Norte	0.728	0.692	0.680	0.819	13	18	8	16	11
Sergipe	0.702	0.662	0.684	0.764	11	24	5	13	20
Southeast									
Espírito Santo	0.771	0.715	0.742	0.864	7	8	6	6	15
Minas Gerais	0.774	0.718	0.762	0.846	2	14	7	21	5
Rio de Janeiro	0.762	0.759	0.758	0.769	25	3	15	26	9
São Paulo	0.806	0.771	0.839	0.810	14	7	3	15	2
North									
Acre	0.710	0.655	0.692	0.788	21	21	10	2	25
Amazonas	0.700	0.641	0.720	0.744	24	25	19	5	21
Amapá	0.688	0.648	0.647	0.778	27	26	21	1	26
Pará	0.690	0.645	0.686	0.744	26	27	25	22	24
Rondônia	0.700	0.677	0.694	0.731	18	4	23	7	16
Roraima	0.699	0.680	0.673	0.745	22	11	24	3	18
Tocantins	0.731	0.684	0.732	0.779	3	5	13	4	12
Central‐West									
Federal District	0.814	0.821	0.817	0.803	20	1	1	—	—
Goiás	0.737	0.714	0.778	0.721	17	16	16	14	10
Mato Grosso do Sul	0.742	0.733	0.741	0.751	9	15	22	8	1
Mato Grosso	0.736	0.720	0.758	0.730	12	17	18	9	3
South									
Paraná	0.769	0.744	0.780	0.785	4	6	9	17	6
Rio Grande do Sul	0.771	0.767	0.750	0.797	8	2	4	18	7
Santa Catarina	0.792	0.759	0.790	0.827	1	9	2	10	4

^a^ The human development index values were calculated by the Atlas of Human Development in Brazil [35]. The following human development index classification ranges were considered: (very low) 0–0.499, (low) 0.500–0.599, (moderate) 0.600–0.699, (high) 0.700–0.799, and (very high) 0.800–1.000.

^b^ The quality of health services was evaluated using indicators obtained from the Institute for Health Policy Studies Portal. The indicators were divided into thematic blocks: (a) basic care, (b) resources, (c) mortality and morbidity, and (d) expenditures (municipal and state) (IEPS Data Portal—iepsdata.org.br) [36]. The thematic blocks were described by the position occupied by each State or Federal District according to the evaluated indicators, i.e. the first position is indicative of better indices in the indicators and, from there, up to position 27, we have a decrease in the evaluated indices (drop in the quality of health according to the evaluated indicators in the thematic block).

With regard to the HDI, the best indices were associated with the Federal District (0.814) and the States of São Paulo (0.806) and Minas Gerais (0.774). On the other hand, the lowest indices were associated with the States of Maranhão (0.676), Alagoas (0.684), and Amapá (0.688). In the income dimension, the best indices were described for the Federal District (0.821), the State of São Paulo (0.771), and the State of Rio Grande do Sul (0.767); and the worst indices, respectively, for the States of Maranhão (0.603), Alagoas (0.630), and Amazonas (0.641). In the education dimension, the best indices were described for the State of São Paulo (0.839), the Federal District (0.817), and the State of Santa Catarina (0.790); and the worst indices, respectively, for the States of Amapá (0.647), Bahia (0.659), and Paraíba (0.669). Finally, in the longevity (life expectancy) dimension, the best indices were described for the States of Espírito Santo (0.864), Minas Gerais (0.846), and Santa Catarina (0.827); and the worst indices, respectively, for the States of Maranhão (0.715), Goiás (0.721), and Amazonas (0.726).

With regard to health quality indicators, five thematic blocks were assessed. In the thematic block associated with primary care, the States with the best positions were Santa Catarina, Minas Gerais, and Tocantins; and those with the worst indices were the States of Amapá, Pará, and Rio de Janeiro. In the thematic block associated with the availability of resources for health, the Federal District had the best position, followed by the States of Rio Grande do Sul and Rio de Janeiro; and those with the worst indices were the States of Pará, Amapá, and Amazonas. In the thematic block associated with mortality and morbidity, the Federal District was also in the best position, followed by the States of Santa Catarina and São Paulo; and the States of Maranhão, Piauí, and Pará had the worst indices. In the thematic block associated with state health spending, the States with the best positions were Amapá, Acre, and São Paulo; and those with the worst indices were the States of Rio de Janeiro, Paraíba, and Maranhão. Lastly, in the thematic block associated with municipal health spending, the States that had the best position were the States of Mato Grosso do Sul, São Paulo, and Mato Grosso; and those with the worst indices were the States of Amapá, Acre, and Pará (Table 3).

### 3.4 Correlation of the Human Development Index and Health Quality Indicators of the Brazilian Federation Units With the Doses of Vaccines Administered in the Quilombola Population of Brazil

In the Spearman correlation between the markers of the HDI and the health quality indicators of Brazil's Federative Units with the doses of vaccines administered in the Quilombola population, the following coefficients were significant:

(a) first dose with municipal health spending [CC = −0.57; 95% CI = −0.79 to −0.24] − moderate CC;
(b) second dose or single‐dose vaccine vial with:
  (i) HDI [CC = 0.58; 95% CI = 0.27 to 0.79] − moderate CC;
  (ii) HDI–income [CC = 0.58; 95% CI = 0.26 to 0.79] − moderate CC;
  (iii) HDI–education [CC = 0.65; 95% CI = 0.36 to 0.83] − moderate CC;
  (iv) municipal spending on health [CC = −0.61; 95% CI = −0.81 to −0.29] − moderate CC.
(c) total number of doses with:
  (i) HDI [CC = 0.58; 95% CI = 0.26 to 0.79] − moderate CC;
  (ii) HDI–income index [CC = 0.56; 95% CI = 0.23 to 0.77] − moderate CC;
  (iii) HDI–education [CC = 0.67; 95% CI = 0.38 to 0.84] − moderate CC;
  (iv) municipal spending on health [CC = −0.55; 95% CI = −0.77 to −0.20] − moderate CC.

In addition, the following data were identified in the exploratory association between the HDI and the markers associated with health quality:

(i) basic care [CC = −0.44; 95% CI = −0.70 to −0.07] − moderate CC;
(ii) health resources [CC = −0.67; 95% CI = −0.84 to −0.39] − moderate CC;
(iii) mortality and morbidity [CC = −0.78; 95% CI = −0.90 to −0.57] − high CC;
(iv) municipal health spending [CC = −0.75; 95% CI = −0.88 to −0.51] − high CC.

**Figure 4:**
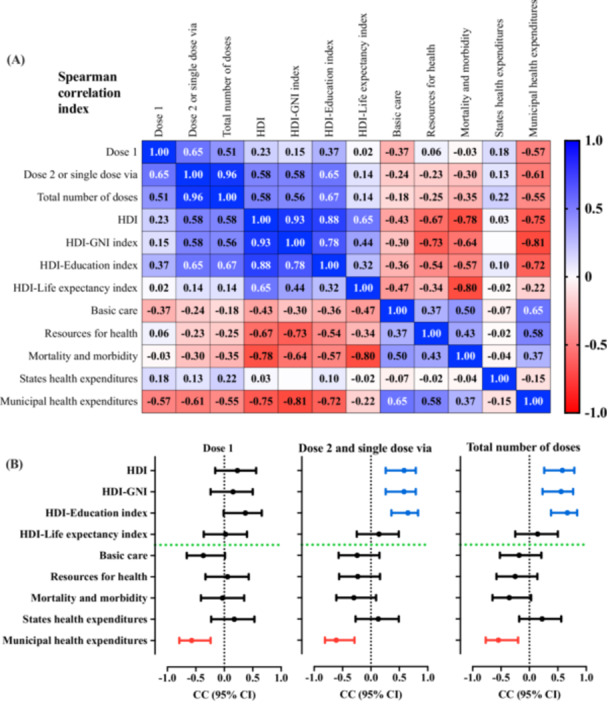
Spearman's correlation coefficient (CC) between the human development index (HDI) and health quality indicators of the Federative Units of Brazil with the doses of vaccines administered in the Quilombola population of Brazil. (A) Correlation matrix for Spearman's CC. (B) Spearman's CC with the description of their respective 95% confidence intervals (95% CI) for the markers associated to the first dose, second dose (or single dose), and for the total number of doses. In (A), the blue markers indicate positive correlation indices, and the red markers indicate negative correlation indices. In (B), the blue markers indicate significant and positive correlation indices, and the red markers indicate significant and negative correlation indices. The Quilombolas' vaccination coverage rate was calculated using the following formula: [(Number of inhabitants/Number of vaccine doses administered) * 100]. In this way, the index shows the number of doses per 100 inhabitants, being representative of the vaccination coverage (%) of the population served in relation to the first and second (or single dose) doses of the vaccines against coronavirus disease (COVID)‐19. The number of doses of vaccines administered was obtained from the National Health Data Network, Ministry of Health of Brazil, updated on 07/31/2024. The HDI values were calculated by the Atlas of Human Development in Brazil [35]. The quality of health services was assessed using indicators obtained on the Institute for Health Policy Studies portal. The indicators were divided into thematic blocks: (a) basic care, (b) resources, (c) mortality and morbidity, and (d) spending (municipal and state) (IEPS Data Portal—iepsdata.org.br) [36]. The thematic blocks were described by the position occupied by each State or Federal District according to the indicators evaluated, that is, the first position is indicative of better indices in the indicators and, from there, up to position 27, we have a decrease in the evaluated indices (drop in the quality of health according to the indicators evaluated in the thematic block). GNI, Gross National Income.

The full overview of the correlations is shown in Table 4 and Figure 4a,b.

**Table 4:** Spearman's correlation coefficient between the human development index (HDI) and health quality indicators of the Brazilian Federation Units with the doses of vaccines administered in the Quilombola population in Brazil.^a^ ^,^ ^b^ ^,^ ^c^

Markers		Dose 1	Dose 2 or single dose via	Total number of doses	HDI	HDI–GNI index	HDI–education index	HDI–life expectancy index	Basic care	Resources for health	Mortality and morbidity	States health expenditures
Dose 2 or single dose via	CC	0.654	1									
	95% CI	0.36 to 0.83	—									
	*p*	< 0.001	—									
Total number of doses	CC	0.508	0.956	1								
	95% CI	0.16 to 0.74	0.90 to 0.98	—								
	*p*	0.007	< 0.001	—								
HDI	CC	0.231	0.584	0.580	1							
	95% CI	−0.16 to 0.56	0.26 to 0.79	0.26 to 0.79	—							
	*p*	0.246	0.001	0.002	—							
HDI–GNI index	CC	0.154	0.582	0.558	0.930	1						
	95% CI	−0.24 to 0.50	0.26 to 0.79	0.23 to 0.77	0.85 to 0.97	—						
	*p*	0.442	0.001	0.003	< 0.001	—						
HDI–education index	CC	0.371	0.650	0.667	0.880	0.781	1					
	95% CI	−0.01 to 0.66	0.36 to 0.83	0.38 to 0.84	0.75 to 0.94	0.57 to 0.90	—					
	*p*	0.057	< 0.001	< 0.001	< 0.001	< 0.001	—					
HDI–life expectancy index	CC	0.021	0.141	0.144	0.646	0.445	0.318	1				
	95% CI	−0.36 to 0.40	−0.25 to 0.49	−0.25 to 0.50	0.35 to 0.82	0.08 to 0.71	−0.07 to 0.62	—				
	*p*	0.919	0.484	0.473	< 0.001	0.020	0.106	—				
Basic care	CC	−0.368	−0.243	−0.181	−0.435	−0.296	−0.357	−0.469	1			
	95% CI	−0.66 to 0.013	−0.57 to 0.15	−0.52 to 0.21	−0.70 to −0.07	−0.61 to 0.09	−0.65 to 0.03	−0.72 to −0.11	—			
	*p*	0.056	0.221	0.367	0.023	0.133	0.067	0.013	—			
Resources for health	CC	0.057	−0.232	−0.251	−0.670	−0.726	−0.539	−0.342	0.373	1		
	95% CI	−0.33 to 0.43	−0.56 to 0.16	−0.58 to 0.14	−0.84 to −0.39	−0.87 to −0.48	−0.76 to −0.20	−0.64 to 0.044	−0.01 to 0.66	—		
	*p*	0.778	0.244	0.206	< 0.001	< 0.001	0.004	0.081	0.055	—		
Mortality and morbidity	CC	−0.032	−0.300	−0.355	−0.781	−0.642	−0.566	−0.802	0.498	0.429	1	
	95% CI	−0.41 to 0.35	−0.61 to 0.09	−0.65 to 0.03	−0.90 to −0.57	−0.82 to −0.35	−0.78 to −0.24	−0.91 to −0.61	0.14 to 0.74	0.06 to 0.70	—	
	*p*	0.876	0.128	0.069	< 0.001	< 0.001	0.002	< 0.001	0.008	0.025	—	
States health expenditures	CC	0.176	0.129	0.224	0.035	0.004	0.096	−0.017	−0.067	−0.019	−0.040	1
	95% CI	−0.23 to 0.53	−0.27 to 0.49	−0.18 to 0.56	−0.36 to 0.42	−0.38 to 0.39	−0.30 to 0.47	−0.40 to 0.37	−0.44 to 0.33	−0.40 to 0.37	−0.42 to 0.35	—
	*p*	0.390	0.529	0.270	0.866	0.986	0.641	0.934	0.744	0.925	0.846	—
Municipal health expenditures	CC	−0.573	−0.611	−0.546	−0.749	−0.809	−0.718	−0.220	0.652	0.584	0.367	−0.146
	95% CI	−0.79 to −0.24	−0.81 to −0.29	−0.77 to −0.20	−0.88 to −0.51	−0.91 to −0.61	−0.86 to −0.46	−0.56 to 0.18	0.35 to 0.83	0.25 to 0.79	−0.02 to 0.66	−0.50 to 0.26
	*p*	0.002	< 0.001	0.004	< 0.001	< 0.001	< 0.001	0.280	< 0.001	0.002	0.065	0.477

*Note:* The grey shaded area indicates the correlations that were found to be statistically significant.

^a^ The correlation between the markers was carried out using the Spearman correlation test. The following cut‐off points were considered for Spearman's correlation: (i) ±0.90–1.0 [very high positive–negative correlation index], (ii) ±0.70–0.89 [high positive–negative correlation index], (iii) ±0.40–0.69 [moderate positive–negative correlation index], (iv) ±0.10–0.39 [low positive–negative correlation index], and (v) 0.00–0.10 [insignificant positive‐negative correlation index] [37]. An alpha error of 0.05 was used in the statistical analysis. In addition, the correlation values are presented by the correlation coefficient (CC) and its respective 95% confidence interval (95% CI).

^b^ The human development index values were calculated by the Atlas of Human Development in Brazil [35]. The following Human Development Index classification ranges were considered: (very low) 0–0.499, (low) 0.500–0.599, (moderate) 0.600–0.699, (high) 0.700–0.799, and (very high) 0.800–1.000.

^c^ The quality of health services was assessed using indicators obtained from the Institute for Health Policy Studies Portal. The indicators were divided into thematic blocks: (a) basic care, (b) resources, (c) mortality and morbidity, and (d) spending (municipal and state) (IEPS Data Portal—iepsdata.org.br) [36]. The thematic blocks were described by the position occupied by each State or Federal District according to the indicators evaluated, i.e. the first position is indicative of better indices in the indicators and, from there, up to position 27, we have a decrease in the indices evaluated (drop in the quality of health according to the indicators evaluated in the thematic block). GNI, Gross National Income.

### 3.5 Complete Overview of the Association Between the Assessed Markers

**Figure 5:**
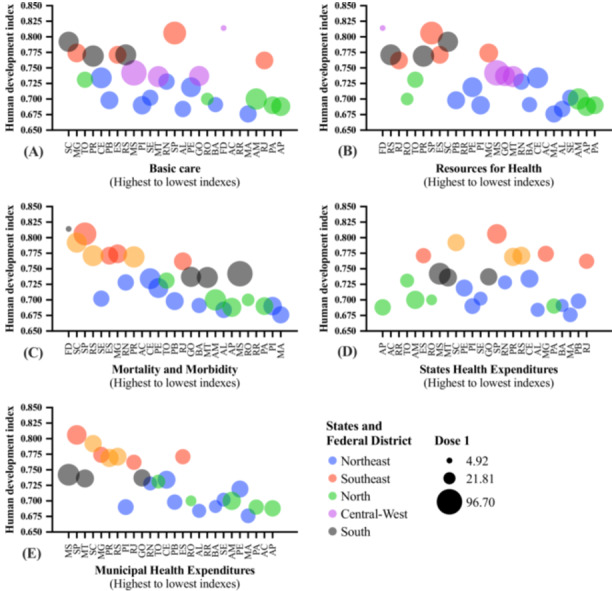
Overview of the association between vaccination coverage for the first dose of the coronavirus disease (COVID)‐19 vaccine in the Quilombola population of Brazil according to the human development index, quality of health services, Federation Units, and macroregions (South, Central‐West, North, Southeast, and Northeast). The vaccination coverage rate for Quilombolas was calculated using the following formula: [(Number of inhabitants/Number of vaccine doses administered) * 100]. In this way, the index shows the number of doses per 100 inhabitants and is representative of the vaccination coverage (%) of the population served in relation to the first dose of COVID‐19 vaccines. The number of doses of vaccines administered was obtained from the National Health Data Network, Brazilian Ministry of Health, updated on 07/31/2024. The human development index values were calculated by the Atlas of Human Development in Brazil [35]. The quality of health services was assessed using indicators obtained from the Institute for Health Policy Studies portal. The indicators were divided into thematic blocks: (A) basic care, (B) resources, (C) mortality and morbidity, (D) state health spending, and (E) municipal health spending (IEPS Data Portal—iepsdata.org.br) [36]. The thematic blocks were described by the position occupied by each State or Federal District according to the indicators assessed, ‐that is, the first position is indicative of better indices in the indicators and, from there, up to position 27, we have a decrease in the indices assessed (drop in the quality of health according to the indicators assessed in the thematic block). [Northeast] AL, Alagoas; BA, Bahia; CE, Ceará; MA, Maranhão; PB, Paraíba; PE, Pernambuco; PI, Piauí; RN, Rio Grande do Norte; SE, Sergipe; [Southeast] ES, Espírito Santo; MG, Minas Gerais; RJ, Rio de Janeiro; SP, São Paulo; [North] AC, Acre; AM, Amazonas; AP, Amapá; PA, Pará; RO, Rondônia; RR, Roraima; TO, Tocantins; [Central‐West] FD, Federal District; GO, Goiás; MS, Mato Grosso do Sul; MT, Mato Grosso; [South] PR, Paraná; RS, Rio Grande do Sul; SC, Santa Catarina.

**Figure 6:**
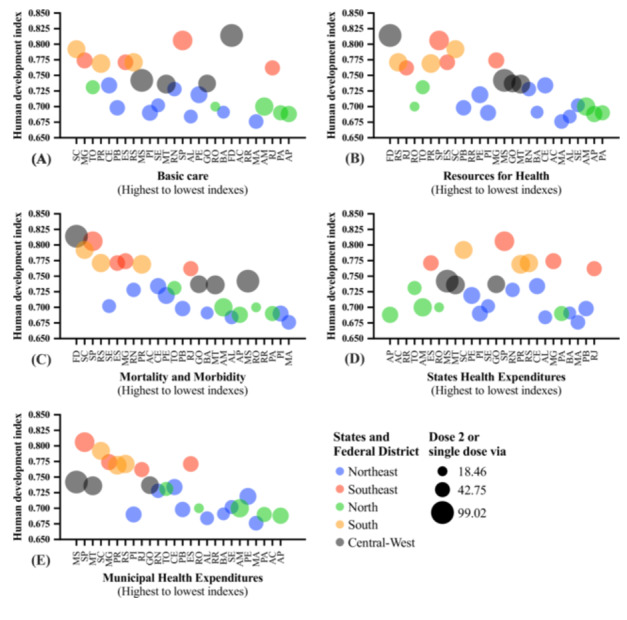
Overview of the association between vaccination coverage for the second dose of the coronavirus disease (COVID)‐19 vaccine in the Quilombola population of Brazil according to the human development index, quality of health services, Federation Units, and macroregions (South, Central‐West, North, Southeast, and Northeast). The vaccination coverage rate for Quilombolas was calculated using the following formula: [(Number of inhabitants/Number of vaccine doses administered) * 100]. In this way, the index shows the number of doses per 100 inhabitants and is representative of the vaccination coverage (%) of the population served in relation to the second dose of COVID‐19 vaccines. The number of doses of vaccines administered was obtained from the National Health Data Network, Brazilian Ministry of Health, updated on 07/31/2024. The human development index values were calculated by the Atlas of Human Development in Brazil [35]. The quality of health services was assessed using indicators obtained from the Institute for Health Policy Studies portal. The indicators were divided into thematic blocks: (A) basic care, (B) resources, (C) mortality and morbidity, (D) state health spending, and (E) municipal health spending (IEPS Data Portal—iepsdata.org.br) [36]. The thematic blocks were described by the position occupied by each State or Federal District according to the assessed indicators, that is, the first position is indicative of better indices in the indicators and, from there, up to position 27, we have a decrease in the assessed indices (drop in the quality of health according to the indicators assessed in the thematic block). [Northeast] AL, Alagoas; BA, Bahia; CE, Ceará; MA, Maranhão; PB, Paraíba; PE, Pernambuco; PI, Piauí; RN, Rio Grande do Norte; SE, Sergipe; [Southeast] ES, Espírito Santo; MG, Minas Gerais; RJ, Rio de Janeiro; SP, São Paulo; [North] AC, Acre; AM, Amazonas; AP, Amapá; PA, Pará; RO, Rondônia; RR, Roraima; TO, Tocantins; [Central‐West] FD, Federal District; GO, Goiás; MS, Mato Grosso do Sul; MT, Mato Grosso; [South] PR, Paraná; RS, Rio Grande do Sul; SC, Santa Catarina.

**Figure 7:**
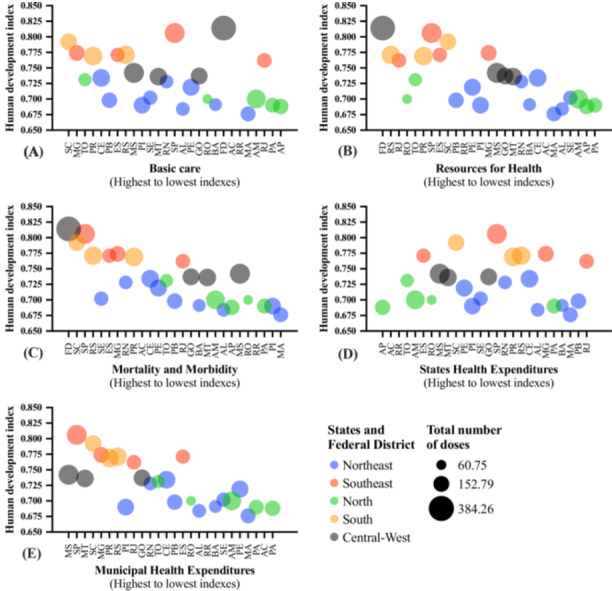
Overview of the association between vaccination coverage for the total number of doses of the coronavirus disease (COVID)‐19 vaccine in the Quilombola population of Brazil according to the human development index, quality of health services, Federation Units, and macroregions (South, Central‐West, North, Southeast, and Northeast). The vaccination coverage rate for Quilombolas was calculated using the following formula: [(Number of inhabitants/Number of vaccine doses administered) * 100]. Thus, the index shows the number of doses per 100 inhabitants. The number of doses of vaccines administered was obtained from the National Health Data Network, Brazilian Ministry of Health, updated on 07/31/2024. The human development index values were calculated by the Atlas of Human Development in Brazil [35]. The quality of health services was assessed using indicators obtained from the Institute for Health Policy Studies Portal. The indicators were divided into thematic blocks: (A) basic care, (B) resources, (C) mortality and morbidity, (D) state health spending, and (E) municipal health spending (IEPS Data Portal—iepsdata.org.br) [36]. The thematic blocks were described by the position occupied by each State or Federal District according to the indicators assessed, i.e. the first position is indicative of better indices in the indicators and, from there, up to position 27, we have a decrease in the indices assessed (drop in the quality of health according to the indicators assessed in the thematic block). [Northeast] AL, Alagoas; BA, Bahia; CE, Ceará; MA, Maranhão; PB, Paraíba; PE, Pernambuco; PI, Piauí; RN, Rio Grande do Norte; SE, Sergipe; [Southeast] ES, Espírito Santo; MG, Minas Gerais; RJ, Rio de Janeiro; SP, São Paulo; [North] AC, Acre; AM, Amazonas; AP, Amapá; PA, Pará; RO, Rondônia; RR, Roraima; TO, Tocantins; [Central‐West] FD, Federal District; GO, Goiás; MS, Mato Grosso do Sul; MT, Mato Grosso; [South] PR, Paraná; RS, Rio Grande do Sul; SC, Santa Catarina.

The presentation of the association between the HDI and markers associated with health quality with Brazil's macroregions and vaccination coverage for the first dose of the COVID‐19 vaccine administered in the Quilombola population is shown in Figure 5a–e. The presentation of the association between the HDI and markers associated with health quality with Brazil's macroregions and vaccination coverage for the second dose or single‐dose vaccine vial of the COVID‐19 vaccine administered in the Quilombola population is shown in Figure 6a–e. The presentation of the association between the HDI and markers associated with health quality with Brazil's macroregions and the total number of doses of the COVID‐19 vaccine administered in the Quilombola population is shown in Figure 7a–e. An overview of the study is presented in Supporting Figure S1.

## 4 Discussion

In Brazil, a total of 1 848 494 doses of COVID‐19 vaccines were administered in the Quilombola population, mostly in the Northeast and Southeast regions, with the ChAdOx‐1 nCov‐19 vaccine (Fiocruz/AstraZeneca) being the most utilized. There was a high degree of variability between Brazil's States and the Federal District in vaccination coverage for the first and second doses of the vaccines, as well as in the number of doses administered per 100 inhabitants. Among the markers associated with this variability, the study found an association between the HDI and the quality of health services offered to the population with vaccination coverage. In terms of age group, the adult population was associated with the highest number of doses administered.

Historically, when it comes to vaccination coverage, the so‐called high‐income countries hold most of the inputs needed to produce vaccines and, therefore, to carry out mass vaccination [38, 39, 40]. This is due to the discrepancies in research infrastructure and health facilities [40], which condition low‐ and middle‐income countries with little chance of dealing effectively with health crises [38, 39]. This is a major concern given that mass vaccination is essential for treatment efficacy being the only way to concomitantly mitigate mortality rates, pathogen transmission, hospitalizations, and long‐term sequelae [38, 41], as well as protecting those who cannot be vaccinated for medical reasons [38]. This discrepancy has been evident at various times in history, such as the pandemics caused by the H1N1 and SARS‐CoV‐2 viruses [42]. For example, in March 2023, more than 70% of the population of developed countries had been vaccinated with the first dose of the COVID‐19 vaccine, to the detriment of around 30% of the population of underdeveloped countries [38]. Given the visible need for equitable access to vaccination, COVID‐19 Vaccines Global Access (COVAX) was created at the beginning of the pandemic, with the intention of distributing vaccines to the most disadvantaged countries [39, 43]. However, despite good progress, there are still considerable differences to be resolved [43].

Despite its many social inequalities, Brazil is known and respected worldwide for being a benchmark in vaccination campaigns. This achievement was consolidated mostly after the creation of the National Immunization Program in 1975, which is currently part of the Unified Health System [41, 44]. However, in line with the described global scenario, Brazil has made up the list of countries in which vaccination against COVID‐19 has been unequal between the richest and poorest [38, 44]. Proof of this is the higher vaccination coverage in municipalities with a high HDI compared to those with medium or low indices [44]. In addition to the above, another factor that contributed to maintaining disparities in relation to vaccination was vaccine hesitancy [41, 45], which occurred especially among supporters of the antivaccine movement [41]. In view of this, it is clear that in order to achieve equity in vaccination coverage among the most vulnerable, it is also necessary to consider their socioeconomic conditions [45].

The COVID‐19 pandemic has substantially worsened the socioeconomic situation of vulnerable populations, defined as individuals who face systemic exclusion and discrimination based on age, disability, race, ethnicity, income level, religion, caste or creed, gender identity, sexual orientation, immigrants, refugees, prisoners, individuals with chronic health conditions, individuals with low socioeconomic conditions, and workers who have been exposed to the pandemic [46]. During the pandemic, vulnerability worsened in Brazil, especially in groups such as Quilombolas and Indigenous communities who already faced high levels of comorbidities, infectious diseases, and difficulties in accessing health services [16, 17, 19, 20, 47, 48]. These groups were prioritized by the government to receive doses of the COVID‐19 vaccine [49]. However, the territorial recognition of these groups has implied in the still limited access to health services and vaccines [25]. Furthermore, a study has shown that prioritizing the most affected countries and developing immunity in the most susceptible populations, through equitable vaccine allocation strategies, can substantially reduce the spread of new viral strains related to COVID‐19 [39].

Several obstacles have been observed such as socioeconomic vulnerability, access to health services, and distribution of vaccines in vulnerable groups; for example, racially, the COVID‐19 mortality rate was 1.5 times higher among Black people compared to White people [50]. In the literature, one study indicated that African Americans were less likely to receive confirmatory COVID‐19 tests compared to Whites due to limited access to primary care and testing centers, and thus there were additional barriers to diagnosis and potentially, to treatment [51].

Socially and economically, in the South and Southeast regions of Brazil, men, Black people, and low‐income groups were more likely to be partially vaccinated due to the lack or delay of vaccine doses [45]. Additionally, municipalities in Brazil with a lower proportion of Black population, higher educational level, and higher per capita income had a significantly higher vaccination coverage compared to municipalities with opposite characteristics [38, 39]. Communication inequality was also evident, demonstrated by the population's difficulty in interpreting scientific information about the pandemic and vaccines, as well as the dissemination of false information on social media [52].

The budget used by the Brazilian government to subsidize health was around 8% in the first quarters of 2019, however, in 2020 this figure increased to 30% more than previously, yet only 8% was spent to tackle the pandemic. However, this did not prevent Brazil from obtaining one of the highest numbers of confirmed cases and deaths in the world, especially due to the second wave of the pandemic that affected more remote and vulnerable regions, such as the States in the North and Northeast regions of the country [49]. Consequently, it has been indicated that prioritizing vaccination in countries with large populations can reduce the overall prevalence in the long term [39], showing that the decisions made by the State and federal governments to invest only 8% in health were irresponsible toward the population of Brazil, mostly the vulnerable, as well as reinforcing a second wave of COVID‐19.

In countries with a high risk of new COVID‐19 cases, diagnostic tests, pharmaceutical products, and vaccines have been developed more quickly than in low‐ and middle‐income countries [40]. Thus, vaccines have been acquired through multilateral agreements, donations, and mechanisms such as COVAX and AVaTAR (Accelerated Vaccine Triage and Response), with 2.5% of all vaccines acquired globally being through donations [43]. In relation to countries with low mortality, there has been a greater benefit from the allocation of vaccines because donations by high‐income countries can protect high‐income countries as well as low‐income countries and middle‐income countries, reducing the risk of future waves of infection [39]. However, some limitations can be described, such as the COVAX initiative which accelerated vaccine development, but without sufficient adherence from low‐ and middle‐income countries due to vaccine nationalism [42].

The COVID‐19 pandemic has brought to light the social and health inequalities that affect the routine of the Quilombola population in Brazil. The prevalence of chronic diseases such as systemic arterial hypertension and diabetes mellitus is higher among the Black population, which increases the risk of serious complications and deaths from COVID‐19 [4, 5]. Moreover, institutional racism perpetuates these inequalities, hindering access to adequate and quality health services for these communities [5]. Low income and less schooling are also factors that increase the vulnerability of Quilombolas, since many do not work and depend on government benefits [8].

As a result, the death of Quilombola individuals, especially the elderly, has had a psychosocial and cultural impact, representing not only a physical loss but also a cultural, political, and religious extermination. They are guardians of history and collective memory whose loss weakens the identity and social cohesion of Quilombola communities [5]. The psychosocial repercussions of the pandemic are profound, aggravated by the historical awareness of marginalization and racial prejudice. Among the territorial challenges, the lack of regularization of Quilombola territories has also contributed to the difficulty of access to vaccination, testing, and medical care, further exposing these populations to pandemic risks [25].

Less than 7% of Quilombola territories have been regularized by the National Institute for Colonization and Agrarian Reform, making it difficult to implement effective health and protection measures during the COVID‐19 pandemic [25]. Of the 23 States in Brazil that have made vaccination plans available, only 12 included Quilombolas among the priority groups, and many did not inform at what stage the vaccine should be administered to this group [25]. Furthermore, there have been disparities in the health of the Quilombola population due to significant barriers in terms of access to primary care and vaccination coverage.

The World Health Organization has recommended global vaccination coverage of at least 90% to contain the pandemic, but Brazil has only achieved 52.8% [28]. This gap becomes even more pronounced among the Quilombola and Indigenous populations who have the lowest vaccination rates in the country, with a national rate of only 0.62/100 inhabitants [25]. In the South and Southeast of Brazil, vaccination coverage was higher, while in the Northeast, there was a relative prioritization, with 40.6% of Quilombolas vaccinated. However, in Brazil as a whole, most vaccination schedules were incomplete [45, 53].

The spatial distribution of COVID‐19 cases in Quilombola communities has not been homogeneous, with clusters of cases in areas close to municipal centers or highways. This may be related to subsistence work activities such as commercialization, agriculture, extractivism, and handicrafts carried out mostly by the women responsible for family maintenance, who did not interrupt their work activities, even if this posed risks to their health [27].

The socioeconomic disparities and low or medium human development have been associated with lower vaccination rates [44]. However, primary health care coverage can play a crucial role in mitigating these disparities as it promotes improved vaccination rates in municipalities with a low HDI. Thus, territorial recognition of Quilombola communities, massive investment in primary care in these regions, and social, educational, artistic, and political development are alternatives that can change the current scenario in Brazil [44].

In this regard, international experiences demonstrate the positive impact of innovative strategies in expanding vaccination coverage, especially when multiple vaccination sites and local stakeholders are involved. For example, in Italy, the inclusion of pharmacies as vaccination points has significantly increased uptake, as seen in the Lombardy region where 46% of COVID‐19 vaccines were administered in pharmacies during the 2023 campaign, highlighting the importance of expanding access points for vulnerable populations [30]. In other Italian regions, practices such as school‐based vaccination, involvement of family pediatricians, and hospitals focusing on vulnerable groups have also led to improved immunization rates, demonstrating that diversifying vaccination venues and professionals is effective in increasing coverage [31]. Furthermore, the pandemic underscored the importance of resilient health systems, effective communication, and community engagement for successful vaccination campaigns. Partnerships with local leaders and the integration of digital tools were essential in overcoming vaccine hesitancy and promoting equitable access—lessons that are crucial for vulnerable contexts like Quilombola communities [32].

These findings suggest that public policies in Brazil could be enhanced by adopting innovative and context‐specific territorial, educational, and social practices to promote inclusion and health equity for Quilombolas and other historically marginalized groups. International cooperation could play a key role in this process, like the approach adopted by the United Kingdom's National Institute for Health Research during the pandemic, in which studies, especially in the area of surgery, were conducted with the aim of generating rapid and effective evidence on the impacts of COVID‐19 [54, 55, 56, 57, 58]. Furthermore, there is a need to expand research to include other infectious agents of emerging relevance in Brazil, such as influenza, monkeypox, *Bordetella pertussis* (whooping cough), the Oropouche virus, and the dengue virus [59, 60, 61, 62], considering their potential effects on vulnerable populations.

In view of the results found, it is noticeable that the numerous socioeconomic inequalities related to Quilombola people continually affect their lives, affecting even on their access to vaccination. It is therefore imperative that the particularities of these people are understood and respected, so that they can be effectively included in society, reducing their process of social invisibility. It is hoped that the health, education, and infrastructure aspects of the Quilombola people will improve, since basic living conditions, including vaccination, should be made available to all.

### 4.1 Limitations

This study has some limitations that may affect the interpretation of the results. The main constraints identified are detailed below:

*Lack of disaggregation by sex:* The data set does not differentiate by sex regarding the number and type of doses administered to the Quilombola population by region and Federation Unit, which limits more precise stratified analyses.

*Ambiguity in dose type per Federation Unit:* The type of dose received is not specified per Federation Unit, leading to ambiguity. It is unclear whether the reported doses reflect a large number of individuals receiving fewer types of doses or a smaller group receiving a wider variety of doses.

*Age group data limitations:* Although the data report the total number of doses administered per age group, the number of Quilombola individuals in each group is unavailable. This prevents calculating the ratio of doses administered relative to the population size in each age stratum.

*Discrepancies in population estimates:* The number of Quilombola individuals provided by the National Health Data Network differs from that reported by IBGE. Despite a 2‐year difference between the data sources, this discrepancy is unlikely due to natural demographic changes and probably reflects differences in the timing of data collection.

*Outdated vaccination registry data:* The vaccination registry relies solely on values from the National Health Data Network, which are not updated in line with the current Quilombola population. In contrast, the IBGE census offers a more accurate and up‐to‐date count.

*Lack of specific COVID‐19 mortality data:* The available data do not provide disaggregated COVID‐19 mortality counts for the Quilombola population, as this group is included within the broader category of the Black population in Brazil, limiting specific mortality analysis.

## 5 Conclusions

The epidemiological profile of vaccination against COVID‐19 in the Quilombola population in Brazil was associated with wide variability in relation to macroregions and Federative Units, with few Federative Units vaccinating more than 50% of Quilombola individuals. Among the markers evaluated, the best HDI and the best quality of health services were associated with a better vaccination coverage for the first dose, second dose, single‐dose vaccine vial, and total number of doses administered in the Quilombola population, demonstrating that there is a relation between social and economic characteristics and the management of vaccines with a potential influence on the outcomes associated with the COVID‐19 pandemic.

## Author Contributions

Lucas Silva Mello and Fernando Augusto Lima Marson collected and tabulated the data. Patrícia Teixeira Costa, Lucas Silva Mello, Luiz Felipe Azevedo Marques, Vinícius Santiago dos Santos, and Fernando Augusto Lima Marson interpreted the study findings. Patrícia Teixeira Costa, Lucas Silva Mello, Luiz Felipe Azevedo Marques, Vinícius Santiago dos Santos, and Fernando Augusto Lima Marson wrote and revised the text thoroughly before submitting the manuscript to the scientific journal. Patrícia Teixeira Costa, Lucas Silva Mello, Luiz Felipe Azevedo Marques, Vinícius Santiago dos Santos, and Fernando Augusto Lima Marson approved the manuscript and agreed with its submission to the scientific journal.

## Ethics Statement

The authors have nothing to report.

## Conflicts of Interest

The authors declare no conflicts of interest.

## Supporting information


**Figure S1:** Graphical abstract showing COVID‐19 vaccination coverage among Quilombola communities in Brazil, highlighting its distribution, correlation with the Human Development Index, municipal health expenditures, and quality of the health system.
**Table S1:** Distribution of vaccines against coronavirus disease (COVID)‐19 applied in the Quilombola population of Brazil according to date of vaccination and type of dose.
**Table S2:** Description of the distribution of doses of vaccines against coronavirus disease (COVID)‐19 in the Quilombola population of Brazil according to Federation Units (States and Federal District) and vaccination coverage index (VCI).
**Table S3:** Distribution of the Quilombola population by macro‐region and Federative Unit of Brazil according to the Demographic Census of Brazil (2022) published by the Brazilian Institute of Geography and Statistics (IBGE of the Portuguese *Instituto Brasileiro de Geografia e Estatística*) and the National Health Data Network.
**Table S4:** Distribution of doses administered according to age in the Quilombola population and type of dose.

## Data Availability

The material used to carry out the study will be available upon request to the corresponding researcher and on the Brazilian government platform from the Brazilian Health Ministry entitled Coronavirus Disease (COVID‐19) Vaccines Database (https://infoms.saude.gov.br/).
