# Supplementary material for: Vaccination Coverage Against Coronavirus Disease 2019 in People Living on Quilombos in Brazil and Its Association With the Human Development Index and the Quality of the Health System
Source: J Med Virol. 2025 Aug 5;97(8):e70533. doi: 10.1002/jmv.70533 (PMC12322944; doi:10.1002/jmv.70533)
Supplement: Supplementary file 1 — Figure S1: Graphical abstract showing COVID‐19 vaccination coverage among Quilombola communities in Brazil, highlighting its distribution, correlation with the Human Development Index, municipal health expenditures, and quality of the health system. Table S1: Distribution of vaccines against coronavirus disease (COVID)‐19 applied in the Quilombola population of Brazil according to date of vaccination and type of dose. Table S2: Description of the distribution of doses of vaccines against coronavirus disease (COVID)‐19 in the Quilombola population of Brazil according to Federation Units (States and Federal District) and vaccination coverage index (VCI). Table S3: Distribution of the Quilombola population by macro‐region and Federative Unit of Brazil according to the Demographic Census of Brazil (2022) published by the Brazilian Institute of Geography and Statistics (IBGE of the Portuguese Instituto Brasileiro de Geografia e Estatística) and the National Health Data Network. Table S4: Distribution of doses administered according to age in the Quilombola population and type of dose. [file JMV-97-e70533-s001.docx]

**Supplementary Material**

**Title:** Vaccination Coverage Against Coronavirus Disease 2019 in People Living on Quilombos in Brazil and Its Association With the Human Development Index and the Quality of the Health System

**Short title:** Quilombos and Vaccination

**Authors:** Patrícia Teixeira Costa; Lucas Silva Mello; Luiz Felipe Azevedo Marques; Vinícius Santiago dos Santos; Fernando Augusto Lima Marson

**Affiliations:**

^1^ Laboratory of Molecular Biology and Genetics, Health Sciences Postgraduate Program, São Francisco University, Bragança Paulista, São Paulo, Brazil.

^2^ Laboratory of Clinical and Molecular Microbiology, Health Sciences Postgraduate Program, São Francisco University, Bragança Paulista, São Paulo, Brazil.

^3^ LunGuardian Research Group – Epidemiology of Respiratory and Infectious Diseases, Health Sciences Postgraduate Program, São Francisco University, Bragança Paulista, São Paulo, Brazil.

*** Corresponding Author:** [FALM] Fernando Augusto Lima Marson, BSc, MSc, PhD.

Laboratory of Molecular Biology and Genetics, Laboratory of Clinical and Molecular Microbiology, LunGuardian Research Group – Epidemiology of Respiratory and Infectious Diseases, Health Sciences Postgraduate Program, São Francisco University. Avenida São Francisco de Assis, 218. Jardim São José, Bragança Paulista 12916-900, São Paulo, Brasil. Phone number: +55-19-999752911.

**E-mail:** fernandolimamarson@hotmail.com and fernando.marson@usf.edu.br.

**Study authors’ e-mails and ORCIDs:**

**PTC:** patricia.costa@usf.edu.br

ORCID: 0009-0002-8179-5644

**LSM:** lucas.silva.mello@mail.usf.edu.br

ORCID: 0009-0006-9920-5058

**LFAM:** luiz.azevedo@mail.usf.edu.br

ORCID: 0009-0008-5494-2171

**VSS:** vinicius.santiago.santos@mail.usf.edu.br

ORCID: 0009-0000-9549-6069

**FALM**: fernandolimamarson@hotmail.com and fernando.marson@usf.edu.br

ORCID: 0000-0003-4955-4234


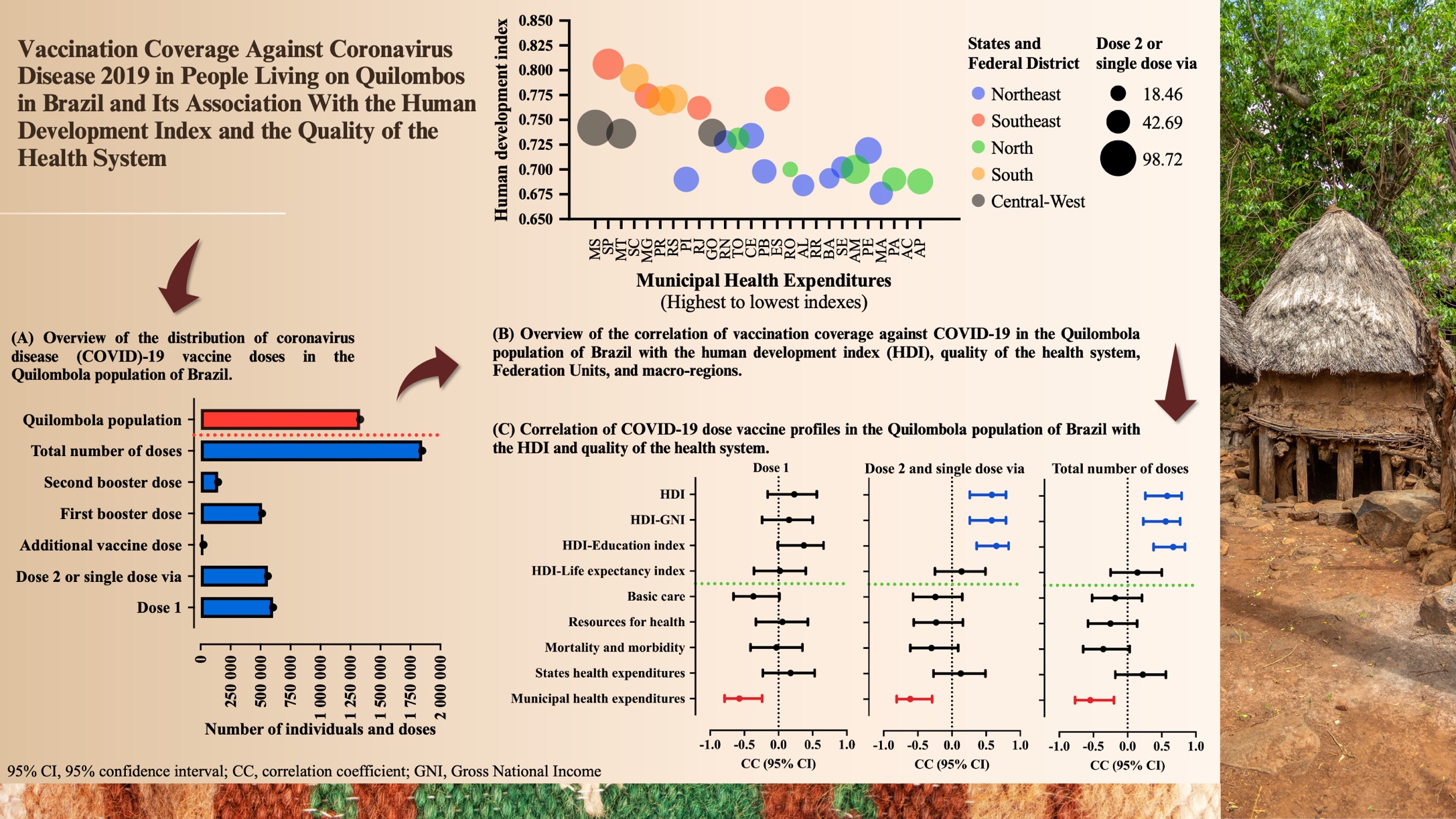


**Figure S1.** Graphical abstract showing COVID-19 vaccination coverage among Quilombola communities in Brazil, highlighting its distribution, correlation with the Human Development Index, municipal health expenditures, and quality of the health system. [Northeast] AL, Alagoas; BA, Bahia; CE, Ceará; MA, Maranhão; PB, Paraíba; PE, Pernambuco; PI, Piauí; RN, Rio Grande do Norte; SE, Sergipe; [Southeast] ES, Espírito Santo; MG, Minas Gerais; RJ, Rio de Janeiro; SP, São Paulo; [North] AC, Acre; AM, Amazonas; AP, Amapá; PA, Pará; RO, Rondônia; RR, Roraima; TO, Tocantins; [Central-West] FD, Federal District; GO, Goiás; MS, Mato Grosso do Sul; MT, Mato Grosso; [South] PR, Paraná; RS, Rio Grande do Sul; SC, Santa Catarina.

| **Supplementary Table 1:** Distribution of vaccines against coronavirus disease (COVID)-19 applied in the Quilombola population of Brazil according to date of vaccination and type of dose. | | | | | | | | | | | | |
| --- | --- | --- | --- | --- | --- | --- | --- | --- | --- | --- | --- | --- |
| **Date** | **Number of doses** | | | | **Cumulative number of doses** | | | | **Cumulative percentage (%)^a^** | | | |
|  | **Total of doses** | **Dose 1** | **Dose 2^b^** | **Booster** | **Total of doses** | **Dose 1** | **Dose 2^b^** | **Booster** | **Total of doses** | **Dose 1** | **Dose 2^b^** | **Booster** |
| 1/17/2021 | 9 | 2 | 1 | 4 | 9 | 2 | 1 | 4 | 0 | 0 | 0 | 0 |
| 1/19/2021 | 19 | 19 | 0 | 0 | 28 | 21 | 1 | 4 | 0 | 0 | 0 | 0 |
| 1/20/2021 | 132 | 129 | 0 | 2 | 160 | 150 | 1 | 6 | 0.01 | 0.02 | 0 | 0 |
| 1/21/2021 | 319 | 314 | 2 | 3 | 479 | 464 | 3 | 9 | 0.03 | 0.08 | 0 | 0 |
| 1/22/2021 | 181 | 179 | 1 | 1 | 660 | 643 | 4 | 10 | 0.04 | 0.11 | 0 | 0 |
| 1/23/2021 | 220 | 219 | 1 | 0 | 880 | 862 | 5 | 10 | 0.05 | 0.14 | 0 | 0 |
| 1/24/2021 | 16 | 14 | 0 | 1 | 896 | 876 | 5 | 11 | 0.05 | 0.14 | 0 | 0 |
| 1/25/2021 | 214 | 214 | 0 | 0 | 1110 | 1090 | 5 | 11 | 0.06 | 0.18 | 0 | 0 |
| 1/26/2021 | 138 | 137 | 1 | 0 | 1248 | 1227 | 6 | 11 | 0.07 | 0.20 | 0 | 0 |
| 1/27/2021 | 201 | 198 | 2 | 1 | 1449 | 1425 | 8 | 12 | 0.08 | 0.24 | 0 | 0 |
| 1/28/2021 | 525 | 524 | 0 | 0 | 1974 | 1949 | 8 | 12 | 0.11 | 0.32 | 0 | 0 |
| 1/29/2021 | 430 | 428 | 2 | 0 | 2404 | 2377 | 10 | 12 | 0.13 | 0.39 | 0 | 0 |
| 1/30/2021 | 330 | 329 | 1 | 0 | 2734 | 2706 | 11 | 12 | 0.15 | 0.45 | 0 | 0 |
| 1/31/2021 | 15 | 14 | 1 | 0 | 2749 | 2720 | 12 | 12 | 0.15 | 0.45 | 0 | 0 |
| 2/1/2021 | 833 | 828 | 5 | 0 | 3582 | 3548 | 17 | 12 | 0.19 | 0.59 | 0 | 0 |
| 2/2/2021 | 900 | 879 | 20 | 1 | 4482 | 4427 | 37 | 13 | 0.24 | 0.73 | 0.01 | 0 |
| 2/3/2021 | 493 | 450 | 43 | 0 | 4975 | 4877 | 80 | 13 | 0.27 | 0.81 | 0.01 | 0 |
| 2/4/2021 | 461 | 455 | 6 | 0 | 5436 | 5332 | 86 | 13 | 0.29 | 0.88 | 0.02 | 0 |
| 2/5/2021 | 185 | 159 | 25 | 0 | 5621 | 5491 | 111 | 13 | 0.30 | 0.91 | 0.02 | 0 |
| 2/6/2021 | 114 | 110 | 4 | 0 | 5735 | 5601 | 115 | 13 | 0.31 | 0.93 | 0.02 | 0 |
| 2/7/2021 | 9 | 8 | 1 | 0 | 5744 | 5609 | 116 | 13 | 0.31 | 0.93 | 0.02 | 0 |
| 2/8/2021 | 882 | 876 | 6 | 0 | 6626 | 6485 | 122 | 13 | 0.36 | 1.07 | 0.02 | 0 |
| 2/9/2021 | 130 | 130 | 0 | 0 | 6756 | 6615 | 122 | 13 | 0.37 | 1.09 | 0.02 | 0 |
| 2/10/2021 | 299 | 281 | 18 | 0 | 7055 | 6896 | 140 | 13 | 0.38 | 1.14 | 0.03 | 0 |
| 2/11/2021 | 367 | 309 | 57 | 1 | 7422 | 7205 | 197 | 14 | 0.40 | 1.19 | 0.04 | 0 |
| 2/12/2021 | 243 | 162 | 81 | 0 | 7665 | 7367 | 278 | 14 | 0.41 | 1.22 | 0.05 | 0 |
| 2/13/2021 | 37 | 14 | 22 | 0 | 7702 | 7381 | 300 | 14 | 0.42 | 1.22 | 0.05 | 0 |
| 2/14/2021 | 47 | 4 | 40 | 2 | 7749 | 7385 | 340 | 16 | 0.42 | 1.22 | 0.06 | 0 |
| 2/15/2021 | 149 | 59 | 90 | 0 | 7898 | 7444 | 430 | 16 | 0.43 | 1.23 | 0.08 | 0 |
| 2/16/2021 | 128 | 52 | 76 | 0 | 8026 | 7496 | 506 | 16 | 0.43 | 1.24 | 0.09 | 0 |
| 2/17/2021 | 369 | 189 | 179 | 0 | 8395 | 7685 | 685 | 16 | 0.45 | 1.27 | 0.12 | 0 |
| 2/18/2021 | 295 | 70 | 225 | 0 | 8690 | 7755 | 910 | 16 | 0.47 | 1.28 | 0.16 | 0 |
| 2/19/2021 | 460 | 70 | 390 | 0 | 9150 | 7825 | 1300 | 16 | 0.49 | 1.29 | 0.23 | 0 |
| 2/20/2021 | 130 | 16 | 114 | 0 | 9280 | 7841 | 1414 | 16 | 0.50 | 1.30 | 0.25 | 0 |
| 2/21/2021 | 15 | 5 | 10 | 0 | 9295 | 7846 | 1424 | 16 | 0.50 | 1.30 | 0.25 | 0 |
| 2/22/2021 | 464 | 67 | 397 | 0 | 9759 | 7913 | 1821 | 16 | 0.53 | 1.31 | 0.33 | 0 |
| 2/23/2021 | 335 | 44 | 291 | 0 | 10 094 | 7957 | 2112 | 16 | 0.55 | 1.32 | 0.38 | 0 |
| 2/24/2021 | 309 | 18 | 291 | 0 | 10 403 | 7975 | 2403 | 16 | 0.56 | 1.32 | 0.43 | 0 |
| 2/25/2021 | 470 | 74 | 390 | 4 | 10 873 | 8049 | 2793 | 20 | 0.59 | 1.33 | 0.50 | 0 |
| 2/26/2021 | 880 | 43 | 837 | 0 | 11 753 | 8092 | 3630 | 20 | 0.64 | 1.34 | 0.65 | 0 |
| 2/27/2021 | 144 | 30 | 114 | 0 | 11 897 | 8122 | 3744 | 20 | 0.64 | 1.34 | 0.67 | 0 |
| 2/28/2021 | 6 | 3 | 2 | 0 | 11 903 | 8125 | 3746 | 20 | 0.64 | 1.34 | 0.67 | 0 |
| 3/1/2021 | 266 | 71 | 195 | 0 | 12 169 | 8196 | 3941 | 20 | 0.66 | 1.36 | 0.70 | 0 |
| 3/2/2021 | 1397 | 96 | 1301 | 0 | 13 566 | 8292 | 5242 | 20 | 0.73 | 1.37 | 0.94 | 0 |
| 3/3/2021 | 469 | 172 | 277 | 20 | 14 035 | 8464 | 5519 | 40 | 0.76 | 1.40 | 0.99 | 0.01 |
| 3/4/2021 | 314 | 116 | 198 | 0 | 14 349 | 8580 | 5717 | 40 | 0.78 | 1.42 | 1.02 | 0.01 |
| 3/5/2021 | 347 | 103 | 236 | 0 | 14 696 | 8683 | 5953 | 40 | 0.80 | 1.44 | 1.06 | 0.01 |
| 3/6/2021 | 83 | 78 | 5 | 0 | 14 779 | 8761 | 5958 | 40 | 0.80 | 1.45 | 1.07 | 0.01 |
| 3/7/2021 | 36 | 36 | 0 | 0 | 14 815 | 8797 | 5958 | 40 | 0.80 | 1.46 | 1.07 | 0.01 |
| 3/8/2021 | 320 | 277 | 42 | 0 | 15 135 | 9074 | 6000 | 40 | 0.82 | 1.50 | 1.07 | 0.01 |
| 3/9/2021 | 326 | 309 | 17 | 0 | 15 461 | 9383 | 6017 | 40 | 0.84 | 1.55 | 1.08 | 0.01 |
| 3/10/2021 | 453 | 350 | 103 | 0 | 15 914 | 9733 | 6120 | 40 | 0.86 | 1.61 | 1.09 | 0.01 |
| 3/11/2021 | 368 | 352 | 16 | 0 | 16 282 | 10 085 | 6136 | 40 | 0.88 | 1.67 | 1.10 | 0.01 |
| 3/12/2021 | 424 | 316 | 108 | 0 | 16 706 | 10 401 | 6244 | 40 | 0.90 | 1.72 | 1.12 | 0.01 |
| 3/13/2021 | 59 | 57 | 2 | 0 | 16 765 | 10 458 | 6246 | 40 | 0.91 | 1.73 | 1.12 | 0.01 |
| 3/14/2021 | 79 | 77 | 0 | 2 | 16 844 | 10 535 | 6246 | 42 | 0.91 | 1.74 | 1.12 | 0.01 |
| 3/15/2021 | 246 | 218 | 28 | 0 | 17 090 | 10 753 | 6274 | 42 | 0.92 | 1.78 | 1.12 | 0.01 |
| 3/16/2021 | 241 | 224 | 17 | 0 | 17 331 | 10 977 | 6291 | 42 | 0.94 | 1.82 | 1.12 | 0.01 |
| 3/17/2021 | 288 | 224 | 64 | 0 | 17 619 | 11 201 | 6355 | 42 | 0.95 | 1.85 | 1.14 | 0.01 |
| 3/18/2021 | 197 | 158 | 38 | 1 | 17 816 | 11 359 | 6393 | 43 | 0.96 | 1.88 | 1.14 | 0.01 |
| 3/19/2021 | 394 | 202 | 192 | 0 | 18 210 | 11 561 | 6585 | 43 | 0.99 | 1.91 | 1.18 | 0.01 |
| 3/20/2021 | 378 | 374 | 4 | 0 | 18 588 | 11 935 | 6589 | 43 | 1.01 | 1.97 | 1.18 | 0.01 |
| 3/21/2021 | 222 | 216 | 5 | 1 | 18 810 | 12 151 | 6594 | 44 | 1.02 | 2.01 | 1.18 | 0.01 |
| 3/22/2021 | 1650 | 1606 | 42 | 0 | 20 460 | 13 757 | 6636 | 44 | 1.11 | 2.28 | 1.19 | 0.01 |
| 3/23/2021 | 4293 | 4278 | 15 | 0 | 24 753 | 18 035 | 6651 | 44 | 1.34 | 2.98 | 1.19 | 0.01 |
| 3/24/2021 | 6958 | 6925 | 33 | 0 | 31 711 | 24 960 | 6684 | 44 | 1.72 | 4.13 | 1.20 | 0.01 |
| 3/25/2021 | 9111 | 9060 | 51 | 0 | 40 822 | 34 020 | 6735 | 44 | 2.21 | 5.63 | 1.20 | 0.01 |
| 3/26/2021 | 10 217 | 10 187 | 29 | 1 | 51 039 | 44 207 | 6764 | 45 | 2.76 | 7.32 | 1.21 | 0.01 |
| 3/27/2021 | 7072 | 6993 | 79 | 0 | 58 111 | 51 200 | 6843 | 45 | 3.14 | 8.47 | 1.22 | 0.01 |
| 3/28/2021 | 634 | 632 | 2 | 0 | 58 745 | 51 832 | 6845 | 45 | 3.18 | 8.58 | 1.22 | 0.01 |
| 3/29/2021 | 12 429 | 12 392 | 36 | 0 | 71 174 | 64 224 | 6881 | 45 | 3.85 | 10.63 | 1.23 | 0.01 |
| 3/30/2021 | 28 311 | 28 267 | 44 | 0 | 99 485 | 92 491 | 6925 | 45 | 5.38 | 15.30 | 1.24 | 0.01 |
| 3/31/2021 | 28 225 | 28 185 | 40 | 0 | 127 710 | 120 676 | 6965 | 45 | 6.91 | 19.97 | 1.25 | 0.01 |
| 4/1/2021 | 16 384 | 16 357 | 26 | 1 | 144 094 | 137 033 | 6991 | 46 | 7.80 | 22.68 | 1.25 | 0.01 |
| 4/2/2021 | 728 | 719 | 9 | 0 | 144 822 | 137 752 | 7000 | 46 | 7.83 | 22.79 | 1.25 | 0.01 |
| 4/3/2021 | 5101 | 5094 | 7 | 0 | 149 923 | 142 846 | 7007 | 46 | 8.11 | 23.64 | 1.25 | 0.01 |
| 4/4/2021 | 1855 | 1849 | 5 | 1 | 151 778 | 144 695 | 7012 | 47 | 8.21 | 23.94 | 1.25 | 0.01 |
| 4/5/2021 | 16 987 | 16 584 | 403 | 0 | 168 765 | 161 279 | 7415 | 47 | 9.13 | 26.69 | 1.33 | 0.01 |
| 4/6/2021 | 21 605 | 21 292 | 313 | 0 | 190 370 | 182 571 | 7728 | 47 | 10.30 | 30.21 | 1.38 | 0.01 |
| 4/7/2021 | 21 215 | 21 017 | 198 | 0 | 211 585 | 203 588 | 7926 | 47 | 11.45 | 33.69 | 1.42 | 0.01 |
| 4/8/2021 | 21 197 | 20 866 | 330 | 0 | 232 782 | 224 454 | 8256 | 47 | 12.59 | 37.14 | 1.48 | 0.01 |
| 4/9/2021 | 17 265 | 16 916 | 349 | 0 | 250 047 | 241 370 | 8605 | 47 | 13.53 | 39.94 | 1.54 | 0.01 |
| 4/10/2021 | 9713 | 9587 | 126 | 0 | 259 760 | 250 957 | 8731 | 47 | 14.05 | 41.53 | 1.56 | 0.01 |
| 4/11/2021 | 1927 | 1923 | 4 | 0 | 261 687 | 252 880 | 8735 | 47 | 14.16 | 41.84 | 1.56 | 0.01 |
| 4/12/2021 | 9977 | 9720 | 257 | 0 | 271 664 | 262 600 | 8992 | 47 | 14.70 | 43.45 | 1.61 | 0.01 |
| 4/13/2021 | 10 986 | 10 752 | 234 | 0 | 282 650 | 273 352 | 9226 | 47 | 15.29 | 45.23 | 1.65 | 0.01 |
| 4/14/2021 | 11 610 | 11 384 | 226 | 0 | 294 260 | 284 736 | 9452 | 47 | 15.92 | 47.12 | 1.69 | 0.01 |
| 4/15/2021 | 9179 | 8846 | 333 | 0 | 303 439 | 293 582 | 9785 | 47 | 16.42 | 48.58 | 1.75 | 0.01 |
| 4/16/2021 | 9549 | 9101 | 448 | 0 | 312 988 | 302 683 | 10 233 | 47 | 16.93 | 50.09 | 1.83 | 0.01 |
| 4/17/2021 | 6209 | 6000 | 209 | 0 | 319 197 | 308 683 | 10 442 | 47 | 17.27 | 51.08 | 1.87 | 0.01 |
| 4/18/2021 | 1258 | 682 | 576 | 0 | 320 455 | 309 365 | 11 018 | 47 | 17.34 | 51.19 | 1.97 | 0.01 |
| 4/19/2021 | 8467 | 8194 | 273 | 0 | 328 922 | 317 559 | 11 291 | 47 | 17.79 | 52.55 | 2.02 | 0.01 |
| 4/20/2021 | 10 724 | 9904 | 818 | 2 | 339 646 | 327 463 | 12 109 | 49 | 18.37 | 54.19 | 2.17 | 0.01 |
| 4/21/2021 | 9245 | 8588 | 657 | 0 | 348 891 | 336 051 | 12 766 | 49 | 18.87 | 55.61 | 2.28 | 0.01 |
| 4/22/2021 | 13 354 | 11 923 | 1431 | 0 | 362 245 | 347 974 | 14 197 | 49 | 19.60 | 57.58 | 2.54 | 0.01 |
| 4/23/2021 | 9770 | 9026 | 744 | 0 | 372 015 | 357 000 | 14 941 | 49 | 20.13 | 59.07 | 2.67 | 0.01 |
| 4/24/2021 | 4465 | 4428 | 37 | 0 | 376 480 | 361 428 | 14 978 | 49 | 20.37 | 59.81 | 2.68 | 0.01 |
| 4/25/2021 | 2818 | 2657 | 161 | 0 | 379 298 | 364 085 | 15 139 | 49 | 20.52 | 60.25 | 2.71 | 0.01 |
| 4/26/2021 | 10 707 | 9697 | 1010 | 0 | 390 005 | 373 782 | 16 149 | 49 | 21.10 | 61.85 | 2.89 | 0.01 |
| 4/27/2021 | 11 930 | 10 463 | 1466 | 1 | 401 935 | 384 245 | 17 615 | 50 | 21.74 | 63.58 | 3.15 | 0.01 |
| 4/28/2021 | 13 174 | 11 345 | 1829 | 0 | 415 109 | 395 590 | 19 444 | 50 | 22.46 | 65.46 | 3.48 | 0.01 |
| 4/29/2021 | 10 086 | 9165 | 921 | 0 | 425 195 | 404 755 | 20 365 | 50 | 23.00 | 66.98 | 3.64 | 0.01 |
| 4/30/2021 | 8471 | 7577 | 894 | 0 | 433 666 | 412 332 | 21 259 | 50 | 23.46 | 68.23 | 3.80 | 0.01 |
| 5/1/2021 | 1877 | 1765 | 112 | 0 | 435 543 | 414 097 | 21 371 | 50 | 23.56 | 68.52 | 3.82 | 0.01 |
| 5/2/2021 | 397 | 396 | 1 | 0 | 435 940 | 414 493 | 21 372 | 50 | 23.58 | 68.59 | 3.82 | 0.01 |
| 5/3/2021 | 7872 | 7270 | 602 | 0 | 443 812 | 421 763 | 21 974 | 50 | 24.01 | 69.79 | 3.93 | 0.01 |
| 5/4/2021 | 10 505 | 9665 | 840 | 0 | 454 317 | 431 428 | 22 814 | 50 | 24.58 | 71.39 | 4.08 | 0.01 |
| 5/5/2021 | 11 264 | 9861 | 1403 | 0 | 465 581 | 441 289 | 24 217 | 50 | 25.19 | 73.02 | 4.33 | 0.01 |
| 5/6/2021 | 9202 | 7846 | 1356 | 0 | 474 783 | 449 135 | 25 573 | 50 | 25.68 | 74.32 | 4.57 | 0.01 |
| 5/7/2021 | 8031 | 6890 | 1141 | 0 | 482 814 | 456 025 | 26 714 | 50 | 26.12 | 75.46 | 4.78 | 0.01 |
| 5/8/2021 | 3277 | 3090 | 187 | 0 | 486 091 | 459 115 | 26 901 | 50 | 26.30 | 75.97 | 4.81 | 0.01 |
| 5/9/2021 | 102 | 97 | 5 | 0 | 486 193 | 459 212 | 26 906 | 50 | 26.30 | 75.99 | 4.81 | 0.01 |
| 5/10/2021 | 5185 | 4559 | 626 | 0 | 491 378 | 463 771 | 27 532 | 50 | 26.58 | 76.74 | 4.92 | 0.01 |
| 5/11/2021 | 6296 | 5653 | 643 | 0 | 497 674 | 469 424 | 28 175 | 50 | 26.92 | 77.68 | 5.04 | 0.01 |
| 5/12/2021 | 6159 | 5446 | 713 | 0 | 503 833 | 474 870 | 28 888 | 50 | 27.26 | 78.58 | 5.17 | 0.01 |
| 5/13/2021 | 5803 | 5161 | 642 | 0 | 509 636 | 480 031 | 29 530 | 50 | 27.57 | 79.43 | 5.28 | 0.01 |
| 5/14/2021 | 6410 | 5604 | 806 | 0 | 516 046 | 485 635 | 30 336 | 50 | 27.92 | 80.36 | 5.42 | 0.01 |
| 5/15/2021 | 2760 | 1929 | 831 | 0 | 518 806 | 487 564 | 31 167 | 50 | 28.07 | 80.68 | 5.57 | 0.01 |
| 5/16/2021 | 1591 | 1552 | 39 | 0 | 520 397 | 489 116 | 31 206 | 50 | 28.15 | 80.94 | 5.58 | 0.01 |
| 5/17/2021 | 4202 | 3322 | 880 | 0 | 524 599 | 492 438 | 32 086 | 50 | 28.38 | 81.49 | 5.74 | 0.01 |
| 5/18/2021 | 5868 | 4183 | 1684 | 0 | 530 467 | 496 621 | 33 770 | 50 | 28.70 | 82.18 | 6.04 | 0.01 |
| 5/19/2021 | 3479 | 3015 | 464 | 0 | 533 946 | 499 636 | 34 234 | 50 | 28.89 | 82.68 | 6.12 | 0.01 |
| 5/20/2021 | 6949 | 5890 | 1059 | 0 | 540 895 | 505 526 | 35 293 | 50 | 29.26 | 83.65 | 6.31 | 0.01 |
| 5/21/2021 | 4960 | 3861 | 1099 | 0 | 545 855 | 509 387 | 36 392 | 50 | 29.53 | 84.29 | 6.51 | 0.01 |
| 5/22/2021 | 1102 | 890 | 212 | 0 | 546 957 | 510 277 | 36 604 | 50 | 29.59 | 84.44 | 6.55 | 0.01 |
| 5/23/2021 | 451 | 217 | 233 | 1 | 547 408 | 510 494 | 36 837 | 51 | 29.61 | 84.47 | 6.59 | 0.01 |
| 5/24/2021 | 2864 | 2324 | 540 | 0 | 550 272 | 512 818 | 37 377 | 51 | 29.77 | 84.86 | 6.68 | 0.01 |
| 5/25/2021 | 2783 | 2066 | 717 | 0 | 553 055 | 514 884 | 38 094 | 51 | 29.92 | 85.20 | 6.81 | 0.01 |
| 5/26/2021 | 3803 | 2880 | 923 | 0 | 556 858 | 517 764 | 39 017 | 51 | 30.12 | 85.68 | 6.98 | 0.01 |
| 5/27/2021 | 4023 | 3217 | 806 | 0 | 560 881 | 520 981 | 39 823 | 51 | 30.34 | 86.21 | 7.12 | 0.01 |
| 5/28/2021 | 3521 | 2678 | 843 | 0 | 564 402 | 523 659 | 40 666 | 51 | 30.53 | 86.65 | 7.27 | 0.01 |
| 5/29/2021 | 995 | 623 | 372 | 0 | 565 397 | 524 282 | 41 038 | 51 | 30.59 | 86.75 | 7.34 | 0.01 |
| 5/30/2021 | 193 | 180 | 13 | 0 | 565 590 | 524 462 | 41 051 | 51 | 30.60 | 86.78 | 7.34 | 0.01 |
| 5/31/2021 | 3106 | 2157 | 948 | 1 | 568 696 | 526 619 | 41 999 | 52 | 30.77 | 87.14 | 7.51 | 0.01 |
| 6/1/2021 | 2571 | 1713 | 858 | 0 | 571 267 | 528 332 | 42 857 | 52 | 30.90 | 87.42 | 7.66 | 0.01 |
| 6/2/2021 | 3724 | 2713 | 1010 | 1 | 574 991 | 531 045 | 43 867 | 53 | 31.11 | 87.87 | 7.84 | 0.01 |
| 6/3/2021 | 1039 | 112 | 927 | 0 | 576 030 | 531 157 | 44 794 | 53 | 31.16 | 87.89 | 8.01 | 0.01 |
| 6/4/2021 | 1190 | 938 | 252 | 0 | 577 220 | 532 095 | 45 046 | 53 | 31.23 | 88.05 | 8.06 | 0.01 |
| 6/5/2021 | 304 | 186 | 118 | 0 | 577 524 | 532 281 | 45 164 | 53 | 31.24 | 88.08 | 8.08 | 0.01 |
| 6/6/2021 | 162 | 153 | 9 | 0 | 577 686 | 532 434 | 45 173 | 53 | 31.25 | 88.10 | 8.08 | 0.01 |
| 6/7/2021 | 1840 | 1255 | 585 | 0 | 579 526 | 533 689 | 45 758 | 53 | 31.35 | 88.31 | 8.18 | 0.01 |
| 6/8/2021 | 2504 | 1362 | 1142 | 0 | 582 030 | 535 051 | 46 900 | 53 | 31.49 | 88.54 | 8.39 | 0.01 |
| 6/9/2021 | 3227 | 2007 | 1220 | 0 | 585 257 | 537 058 | 48 120 | 53 | 31.66 | 88.87 | 8.61 | 0.01 |
| 6/10/2021 | 4558 | 1923 | 2635 | 0 | 589 815 | 538 981 | 50 755 | 53 | 31.91 | 89.19 | 9.08 | 0.01 |
| 6/11/2021 | 4013 | 1394 | 2619 | 0 | 593 828 | 540 375 | 53 374 | 53 | 32.12 | 89.42 | 9.54 | 0.01 |
| 6/12/2021 | 2020 | 408 | 1612 | 0 | 595 848 | 540 783 | 54 986 | 53 | 32.23 | 89.48 | 9.83 | 0.01 |
| 6/13/2021 | 1043 | 137 | 906 | 0 | 596 891 | 540 920 | 55 892 | 53 | 32.29 | 89.51 | 9.99 | 0.01 |
| 6/14/2021 | 4173 | 1120 | 3053 | 0 | 601 064 | 542 040 | 58 945 | 53 | 32.52 | 89.69 | 10.54 | 0.01 |
| 6/15/2021 | 7729 | 1607 | 6122 | 0 | 608 793 | 543 647 | 65 067 | 53 | 32.93 | 89.96 | 11.64 | 0.01 |
| 6/16/2021 | 6906 | 1524 | 5382 | 0 | 615 699 | 545 171 | 70 449 | 53 | 33.31 | 90.21 | 12.60 | 0.01 |
| 6/17/2021 | 7389 | 1506 | 5882 | 1 | 623 088 | 546 677 | 76 331 | 54 | 33.71 | 90.46 | 13.65 | 0.01 |
| 6/18/2021 | 6883 | 1463 | 5420 | 0 | 629 971 | 548 140 | 81 751 | 54 | 34.08 | 90.70 | 14.62 | 0.01 |
| 6/19/2021 | 5637 | 787 | 4850 | 0 | 635 608 | 548 927 | 86 601 | 54 | 34.39 | 90.83 | 15.49 | 0.01 |
| 6/20/2021 | 708 | 122 | 586 | 0 | 636 316 | 549 049 | 87 187 | 54 | 34.42 | 90.85 | 15.59 | 0.01 |
| 6/21/2021 | 9640 | 882 | 8757 | 1 | 645 956 | 549 931 | 95 944 | 55 | 34.94 | 91.00 | 17.16 | 0.01 |
| 6/22/2021 | 13 014 | 746 | 12 268 | 0 | 658 970 | 550 677 | 108 212 | 55 | 35.65 | 91.12 | 19.35 | 0.01 |
| 6/23/2021 | 14 195 | 1486 | 12 709 | 0 | 673 165 | 552 163 | 120 921 | 55 | 36.42 | 91.37 | 21.62 | 0.01 |
| 6/24/2021 | 7173 | 926 | 6247 | 0 | 680 338 | 553 089 | 127 168 | 55 | 36.8 | 91.52 | 22.74 | 0.01 |
| 6/25/2021 | 10 572 | 1723 | 8849 | 0 | 690 910 | 554 812 | 136 017 | 55 | 37.38 | 91.81 | 24.32 | 0.01 |
| 6/26/2021 | 5584 | 535 | 5011 | 0 | 696 494 | 555 347 | 141 028 | 55 | 37.68 | 91.89 | 25.22 | 0.01 |
| 6/27/2021 | 1095 | 89 | 1006 | 0 | 697 589 | 555 436 | 142 034 | 55 | 37.74 | 91.91 | 25.40 | 0.01 |
| 6/28/2021 | 15 242 | 1163 | 14 067 | 0 | 712 831 | 556 599 | 156 101 | 55 | 38.56 | 92.10 | 27.91 | 0.01 |
| 6/29/2021 | 13 834 | 1298 | 12 412 | 0 | 726 665 | 557 897 | 168 513 | 55 | 39.31 | 92.32 | 30.13 | 0.01 |
| 6/30/2021 | 16 700 | 1561 | 15 135 | 0 | 743 365 | 559 458 | 183 648 | 55 | 40.21 | 92.58 | 32.84 | 0.01 |
| 7/1/2021 | 13 873 | 828 | 12 975 | 2 | 757 238 | 560 286 | 196 623 | 57 | 40.97 | 92.71 | 35.16 | 0.01 |
| 7/2/2021 | 9938 | 907 | 9018 | 0 | 767 176 | 561 193 | 205 641 | 57 | 41.50 | 92.86 | 36.77 | 0.01 |
| 7/3/2021 | 5544 | 594 | 4880 | 0 | 772 720 | 561 787 | 210 521 | 57 | 41.80 | 92.96 | 37.65 | 0.01 |
| 7/4/2021 | 736 | 177 | 468 | 2 | 773 456 | 561 964 | 210 989 | 59 | 41.84 | 92.99 | 37.73 | 0.01 |
| 7/5/2021 | 11 259 | 875 | 10 294 | 1 | 784 715 | 562 839 | 221 283 | 60 | 42.45 | 93.13 | 39.57 | 0.01 |
| 7/6/2021 | 11 755 | 767 | 10 972 | 0 | 796 470 | 563 606 | 232 255 | 60 | 43.09 | 93.26 | 41.53 | 0.01 |
| 7/7/2021 | 15 928 | 834 | 15 071 | 1 | 812 398 | 564 440 | 247 326 | 61 | 43.95 | 93.40 | 44.23 | 0.01 |
| 7/8/2021 | 14 296 | 978 | 13 263 | 0 | 826 694 | 565 418 | 260 589 | 61 | 44.72 | 93.56 | 46.60 | 0.01 |
| 7/9/2021 | 12 461 | 1 061 | 11 374 | 0 | 839 155 | 566 479 | 271 963 | 61 | 45.40 | 93.74 | 48.63 | 0.01 |
| 7/10/2021 | 7195 | 405 | 6773 | 1 | 846 350 | 566 884 | 278 736 | 62 | 45.79 | 93.8 | 49.84 | 0.01 |
| 7/11/2021 | 478 | 11 | 467 | 0 | 846 828 | 566 895 | 279 203 | 62 | 45.81 | 93.81 | 49.93 | 0.01 |
| 7/12/2021 | 10 912 | 699 | 10 092 | 3 | 857 740 | 567 594 | 289 295 | 65 | 46.40 | 93.92 | 51.73 | 0.01 |
| 7/13/2021 | 12 393 | 543 | 11 750 | 0 | 870 133 | 568 137 | 301 045 | 65 | 47.07 | 94.01 | 53.83 | 0.01 |
| 7/14/2021 | 12 284 | 628 | 11 627 | 2 | 882 417 | 568 765 | 312 672 | 67 | 47.74 | 94.12 | 55.91 | 0.01 |
| 7/15/2021 | 10 860 | 452 | 10 388 | 1 | 893 277 | 569 217 | 323 060 | 68 | 48.32 | 94.19 | 57.77 | 0.01 |
| 7/16/2021 | 6790 | 680 | 6086 | 0 | 900 067 | 569 897 | 329 146 | 68 | 48.69 | 94.30 | 58.86 | 0.01 |
| 7/17/2021 | 3035 | 245 | 2788 | 1 | 903 102 | 570 142 | 331 934 | 69 | 48.86 | 94.34 | 59.36 | 0.01 |
| 7/18/2021 | 597 | 51 | 472 | 2 | 903 699 | 570 193 | 332 406 | 71 | 48.89 | 94.35 | 59.44 | 0.01 |
| 7/19/2021 | 7732 | 342 | 7378 | 1 | 911 431 | 570 535 | 339 784 | 72 | 49.31 | 94.41 | 60.76 | 0.01 |
| 7/20/2021 | 10 107 | 589 | 9496 | 1 | 921 538 | 571 124 | 349 280 | 73 | 49.85 | 94.51 | 62.46 | 0.01 |
| 7/21/2021 | 8156 | 343 | 7811 | 0 | 929 694 | 571 467 | 357 091 | 73 | 50.29 | 94.56 | 63.86 | 0.01 |
| 7/22/2021 | 9000 | 423 | 8490 | 0 | 938 694 | 571 890 | 365 581 | 73 | 50.78 | 94.63 | 65.37 | 0.01 |
| 7/23/2021 | 7392 | 353 | 7034 | 2 | 946 086 | 572 243 | 372 615 | 75 | 51.18 | 94.69 | 66.63 | 0.01 |
| 7/24/2021 | 1906 | 252 | 1654 | 0 | 947 992 | 572 495 | 374 269 | 75 | 51.28 | 94.73 | 66.93 | 0.01 |
| 7/25/2021 | 125 | 11 | 114 | 0 | 948 117 | 572 506 | 374 383 | 75 | 51.29 | 94.73 | 66.95 | 0.01 |
| 7/26/2021 | 7531 | 327 | 7201 | 2 | 955 648 | 572 833 | 381 584 | 77 | 51.70 | 94.79 | 68.24 | 0.02 |
| 7/27/2021 | 7657 | 337 | 7306 | 3 | 963 305 | 573 170 | 388 890 | 80 | 52.11 | 94.84 | 69.54 | 0.02 |
| 7/28/2021 | 8774 | 493 | 8277 | 0 | 972 079 | 573 663 | 397 167 | 80 | 52.59 | 94.93 | 71.02 | 0.02 |
| 7/29/2021 | 7135 | 426 | 6652 | 1 | 979 214 | 574 089 | 403 819 | 81 | 52.97 | 95.00 | 72.21 | 0.02 |
| 7/30/2021 | 4610 | 432 | 4164 | 0 | 983 824 | 574 521 | 407 983 | 81 | 53.22 | 95.07 | 72.96 | 0.02 |
| 7/31/2021 | 1029 | 120 | 858 | 1 | 984 853 | 574 641 | 408 841 | 82 | 53.28 | 95.09 | 73.11 | 0.02 |
| 8/1/2021 | 141 | 18 | 123 | 0 | 984 994 | 574 659 | 408 964 | 82 | 53.29 | 95.09 | 73.13 | 0.02 |
| 8/2/2021 | 4401 | 451 | 3950 | 0 | 989 395 | 575 110 | 412 914 | 82 | 53.52 | 95.17 | 73.84 | 0.02 |
| 8/3/2021 | 5509 | 380 | 5125 | 0 | 994 904 | 575 490 | 418 039 | 82 | 53.82 | 95.23 | 74.76 | 0.02 |
| 8/4/2021 | 5760 | 574 | 5174 | 1 | 1 000 664 | 576 064 | 423 213 | 83 | 54.13 | 95.32 | 75.68 | 0.02 |
| 8/5/2021 | 4867 | 313 | 4549 | 0 | 1 005 531 | 576 377 | 427 762 | 83 | 54.40 | 95.37 | 76.49 | 0.02 |
| 8/6/2021 | 4329 | 281 | 4048 | 0 | 1 009 860 | 576 658 | 431 810 | 83 | 54.63 | 95.42 | 77.22 | 0.02 |
| 8/7/2021 | 1083 | 153 | 929 | 1 | 1 010 943 | 576 811 | 432 739 | 84 | 54.69 | 95.45 | 77.38 | 0.02 |
| 8/8/2021 | 45 | 14 | 30 | 1 | 1 010 988 | 576 825 | 432 769 | 85 | 54.69 | 95.45 | 77.39 | 0.02 |
| 8/9/2021 | 3379 | 247 | 3132 | 0 | 1 014 367 | 577 072 | 435 901 | 85 | 54.88 | 95.49 | 77.95 | 0.02 |
| 8/10/2021 | 3608 | 188 | 3420 | 0 | 1 017 975 | 577 260 | 439 321 | 85 | 55.07 | 95.52 | 78.56 | 0.02 |
| 8/11/2021 | 4837 | 358 | 4478 | 0 | 1 022 812 | 577 618 | 443 799 | 85 | 55.33 | 95.58 | 79.36 | 0.02 |
| 8/12/2021 | 5010 | 253 | 4757 | 0 | 1 027 822 | 577 871 | 448 556 | 85 | 55.60 | 95.62 | 80.21 | 0.02 |
| 8/13/2021 | 3363 | 232 | 3129 | 1 | 1 031 185 | 578 103 | 451 685 | 86 | 55.79 | 95.66 | 80.77 | 0.02 |
| 8/14/2021 | 408 | 42 | 365 | 0 | 1 031 593 | 578 145 | 452 050 | 86 | 55.81 | 95.67 | 80.84 | 0.02 |
| 8/15/2021 | 25 | 11 | 14 | 0 | 1 031 618 | 578 156 | 452 064 | 86 | 55.81 | 95.67 | 80.84 | 0.02 |
| 8/16/2021 | 2754 | 214 | 2537 | 0 | 1 034 372 | 578 370 | 454 601 | 86 | 55.96 | 95.70 | 81.29 | 0.02 |
| 8/17/2021 | 4392 | 439 | 3951 | 0 | 1 038 764 | 578 809 | 458 552 | 86 | 56.20 | 95.78 | 82.00 | 0.02 |
| 8/18/2021 | 4720 | 644 | 4075 | 0 | 1 043 484 | 579 453 | 462 627 | 86 | 56.45 | 95.88 | 82.73 | 0.02 |
| 8/19/2021 | 2870 | 203 | 2667 | 0 | 1 046 354 | 579 656 | 465 294 | 86 | 56.61 | 95.92 | 83.21 | 0.02 |
| 8/20/2021 | 2426 | 278 | 2147 | 1 | 1 048 780 | 579 934 | 467 441 | 87 | 56.74 | 95.96 | 83.59 | 0.02 |
| 8/21/2021 | 501 | 125 | 376 | 0 | 1 049 281 | 580 059 | 467 817 | 87 | 56.76 | 95.98 | 83.66 | 0.02 |
| 8/22/2021 | 21 | 4 | 16 | 1 | 1 049 302 | 580 063 | 467 833 | 88 | 56.77 | 95.98 | 83.66 | 0.02 |
| 8/23/2021 | 3582 | 102 | 3479 | 1 | 1 052 884 | 580 165 | 471 312 | 89 | 56.96 | 96.00 | 84.28 | 0.02 |
| 8/24/2021 | 2784 | 206 | 2578 | 0 | 1 055 668 | 580 371 | 473 890 | 89 | 57.11 | 96.04 | 84.74 | 0.02 |
| 8/25/2021 | 3154 | 513 | 2641 | 0 | 1 058 822 | 580 884 | 476 531 | 89 | 57.28 | 96.12 | 85.22 | 0.02 |
| 8/26/2021 | 2681 | 113 | 2567 | 0 | 1 061 503 | 580 997 | 479 098 | 89 | 57.43 | 96.14 | 85.67 | 0.02 |
| 8/27/2021 | 2984 | 133 | 2851 | 0 | 1 064 487 | 581 130 | 481 949 | 89 | 57.59 | 96.16 | 86.18 | 0.02 |
| 8/28/2021 | 796 | 23 | 771 | 0 | 1 065 283 | 581 153 | 482 720 | 89 | 57.63 | 96.17 | 86.32 | 0.02 |
| 8/29/2021 | 38 | 4 | 34 | 0 | 1 065 321 | 581 157 | 482 754 | 89 | 57.63 | 96.17 | 86.33 | 0.02 |
| 8/30/2021 | 2402 | 222 | 2180 | 0 | 1 067 723 | 581 379 | 484 934 | 89 | 57.76 | 96.20 | 86.72 | 0.02 |
| 8/31/2021 | 1986 | 115 | 1871 | 0 | 1 069 709 | 581 494 | 486 805 | 89 | 57.87 | 96.22 | 87.05 | 0.02 |
| 9/1/2021 | 2254 | 190 | 2063 | 1 | 1 071 963 | 581 684 | 488 868 | 90 | 57.99 | 96.25 | 87.42 | 0.02 |
| 9/2/2021 | 2280 | 225 | 2055 | 0 | 1 074 243 | 581 909 | 490 923 | 90 | 58.11 | 96.29 | 87.79 | 0.02 |
| 9/3/2021 | 1507 | 306 | 1201 | 0 | 1 075 750 | 582 215 | 492 124 | 90 | 58.20 | 96.34 | 88.00 | 0.02 |
| 9/4/2021 | 301 | 19 | 278 | 0 | 1 076 051 | 582 234 | 492 402 | 90 | 58.21 | 96.34 | 88.05 | 0.02 |
| 9/5/2021 | 99 | 4 | 72 | 0 | 1 076 150 | 582 238 | 492 474 | 90 | 58.22 | 96.34 | 88.07 | 0.02 |
| 9/6/2021 | 558 | 41 | 506 | 1 | 1 076 708 | 582 279 | 492 980 | 91 | 58.25 | 96.35 | 88.16 | 0.02 |
| 9/7/2021 | 151 | 7 | 143 | 1 | 1 076 859 | 582 286 | 493 123 | 92 | 58.26 | 96.35 | 88.18 | 0.02 |
| 9/8/2021 | 2168 | 136 | 2014 | 2 | 1 079 027 | 582 422 | 495 137 | 94 | 58.37 | 96.37 | 88.54 | 0.02 |
| 9/9/2021 | 2646 | 150 | 2475 | 6 | 1 081 673 | 582 572 | 497 612 | 100 | 58.52 | 96.40 | 88.98 | 0.02 |
| 9/10/2021 | 1581 | 121 | 1450 | 1 | 1 083 254 | 582 693 | 499 062 | 101 | 58.60 | 96.42 | 89.24 | 0.02 |
| 9/11/2021 | 292 | 29 | 246 | 3 | 1 083 546 | 582 722 | 499 308 | 104 | 58.62 | 96.42 | 89.29 | 0.02 |
| 9/12/2021 | 22 | 2 | 17 | 2 | 1 083 568 | 582 724 | 499 325 | 106 | 58.62 | 96.42 | 89.29 | 0.02 |
| 9/13/2021 | 1181 | 132 | 1044 | 4 | 1 084 749 | 582 856 | 500 369 | 110 | 58.68 | 96.45 | 89.48 | 0.02 |
| 9/14/2021 | 1468 | 156 | 1306 | 4 | 1 086 217 | 583 012 | 501 675 | 114 | 58.76 | 96.47 | 89.71 | 0.02 |
| 9/15/2021 | 1493 | 141 | 1346 | 4 | 1 087 710 | 583 153 | 503 021 | 118 | 58.84 | 96.50 | 89.95 | 0.02 |
| 9/16/2021 | 1578 | 159 | 1413 | 4 | 1 089 288 | 583 312 | 504 434 | 122 | 58.93 | 96.52 | 90.20 | 0.02 |
| 9/17/2021 | 1243 | 79 | 1161 | 3 | 1 090 531 | 583 391 | 505 595 | 125 | 59.00 | 96.54 | 90.41 | 0.02 |
| 9/18/2021 | 115 | 25 | 83 | 7 | 1 090 646 | 583 416 | 505 678 | 132 | 59.00 | 96.54 | 90.43 | 0.03 |
| 9/19/2021 | 19 | 8 | 11 | 0 | 1 090 665 | 583 424 | 505 689 | 132 | 59.00 | 96.54 | 90.43 | 0.03 |
| 9/20/2021 | 1228 | 211 | 1000 | 8 | 1 091 893 | 583 635 | 506 689 | 140 | 59.07 | 96.58 | 90.61 | 0.03 |
| 9/21/2021 | 1151 | 230 | 912 | 6 | 1 093 044 | 583 865 | 507 601 | 146 | 59.13 | 96.61 | 90.77 | 0.03 |
| 9/22/2021 | 1483 | 257 | 1211 | 11 | 1 094 527 | 584 122 | 508 812 | 157 | 59.21 | 96.66 | 90.99 | 0.03 |
| 9/23/2021 | 1263 | 277 | 955 | 25 | 1 095 790 | 584 399 | 509 767 | 182 | 59.28 | 96.70 | 91.16 | 0.04 |
| 9/24/2021 | 1069 | 211 | 813 | 37 | 1 096 859 | 584 610 | 510 580 | 219 | 59.34 | 96.74 | 91.30 | 0.04 |
| 9/25/2021 | 259 | 39 | 213 | 4 | 1 097 118 | 584 649 | 510 793 | 223 | 59.35 | 96.74 | 91.34 | 0.04 |
| 9/26/2021 | 29 | 16 | 12 | 0 | 1 097 147 | 584 665 | 510 805 | 223 | 59.35 | 96.75 | 91.34 | 0.04 |
| 9/27/2021 | 1265 | 165 | 1017 | 75 | 1 098 412 | 584 830 | 511 822 | 298 | 59.42 | 96.77 | 91.53 | 0.06 |
| 9/28/2021 | 1192 | 244 | 899 | 40 | 1 099 604 | 585 074 | 512 721 | 338 | 59.49 | 96.81 | 91.69 | 0.07 |
| 9/29/2021 | 1536 | 98 | 1352 | 74 | 1 101 140 | 585 172 | 514 073 | 412 | 59.57 | 96.83 | 91.93 | 0.08 |
| 9/30/2021 | 1298 | 294 | 923 | 67 | 1 102 438 | 585 466 | 514 996 | 479 | 59.64 | 96.88 | 92.09 | 0.09 |
| 10/1/2021 | 823 | 96 | 627 | 91 | 1 103 261 | 585 562 | 515 623 | 570 | 59.68 | 96.89 | 92.21 | 0.11 |
| 10/2/2021 | 493 | 68 | 303 | 120 | 1 103 754 | 585 630 | 515 926 | 690 | 59.71 | 96.91 | 92.26 | 0.13 |
| 10/3/2021 | 122 | 16 | 105 | 1 | 1 103 876 | 585 646 | 516 031 | 691 | 59.72 | 96.91 | 92.28 | 0.13 |
| 10/4/2021 | 833 | 57 | 636 | 136 | 1 104 709 | 585 703 | 516 667 | 827 | 59.76 | 96.92 | 92.39 | 0.16 |
| 10/5/2021 | 829 | 99 | 577 | 142 | 1 105 538 | 585 802 | 517 244 | 969 | 59.81 | 96.93 | 92.50 | 0.19 |
| 10/6/2021 | 1070 | 230 | 657 | 179 | 1 106 608 | 586 032 | 517 901 | 1148 | 59.87 | 96.97 | 92.61 | 0.22 |
| 10/7/2021 | 739 | 86 | 530 | 116 | 1 107 347 | 586 118 | 518 431 | 1264 | 59.91 | 96.99 | 92.71 | 0.25 |
| 10/8/2021 | 810 | 239 | 427 | 136 | 1 108 157 | 586 357 | 518 858 | 1400 | 59.95 | 97.03 | 92.78 | 0.27 |
| 10/9/2021 | 100 | 17 | 64 | 18 | 1 108 257 | 586 374 | 518 922 | 1418 | 59.95 | 97.03 | 92.80 | 0.28 |
| 10/10/2021 | 29 | 12 | 14 | 3 | 1 108 286 | 586 386 | 518 936 | 1421 | 59.96 | 97.03 | 92.80 | 0.28 |
| 10/11/2021 | 322 | 42 | 259 | 20 | 1 108 608 | 586 428 | 519 195 | 1441 | 59.97 | 97.04 | 92.84 | 0.28 |
| 10/12/2021 | 55 | 2 | 43 | 9 | 1 108 663 | 586 430 | 519 238 | 1450 | 59.98 | 97.04 | 92.85 | 0.28 |
| 10/13/2021 | 933 | 98 | 732 | 101 | 1 109 596 | 586 528 | 519 970 | 1551 | 60.03 | 97.05 | 92.98 | 0.30 |
| 10/14/2021 | 827 | 87 | 578 | 148 | 1 110 423 | 586 615 | 520 548 | 1699 | 60.07 | 97.07 | 93.09 | 0.33 |
| 10/15/2021 | 634 | 52 | 474 | 103 | 1 111 057 | 586 667 | 521 022 | 1802 | 60.11 | 97.08 | 93.17 | 0.35 |
| 10/16/2021 | 321 | 18 | 275 | 26 | 1 111 378 | 586 685 | 521 297 | 1828 | 60.12 | 97.08 | 93.22 | 0.36 |
| 10/17/2021 | 24 | 1 | 20 | 2 | 1 111 402 | 586 686 | 521 317 | 1830 | 60.12 | 97.08 | 93.22 | 0.36 |
| 10/18/2021 | 617 | 42 | 462 | 107 | 1 112 019 | 586 728 | 521 779 | 1937 | 60.16 | 97.09 | 93.31 | 0.38 |
| 10/19/2021 | 843 | 52 | 660 | 122 | 1 112 862 | 586 780 | 522 439 | 2059 | 60.20 | 97.10 | 93.42 | 0.40 |
| 10/20/2021 | 876 | 118 | 626 | 125 | 1 113 738 | 586 898 | 523 065 | 2184 | 60.25 | 97.12 | 93.54 | 0.43 |
| 10/21/2021 | 680 | 81 | 487 | 106 | 1 114 418 | 586 979 | 523 552 | 2290 | 60.29 | 97.13 | 93.62 | 0.45 |
| 10/22/2021 | 629 | 96 | 397 | 113 | 1 115 047 | 587 075 | 523 949 | 2403 | 60.32 | 97.14 | 93.69 | 0.47 |
| 10/23/2021 | 244 | 20 | 182 | 38 | 1 115 291 | 587 095 | 524 131 | 2441 | 60.34 | 97.15 | 93.73 | 0.48 |
| 10/24/2021 | 10 | 0 | 5 | 5 | 1 115 301 | 587 095 | 524 136 | 2446 | 60.34 | 97.15 | 93.73 | 0.48 |
| 10/25/2021 | 637 | 83 | 430 | 112 | 1 115 938 | 587 178 | 524 566 | 2558 | 60.37 | 97.16 | 93.80 | 0.50 |
| 10/26/2021 | 844 | 124 | 513 | 190 | 1 116 782 | 587 302 | 525 079 | 2748 | 60.42 | 97.18 | 93.90 | 0.54 |
| 10/27/2021 | 913 | 75 | 638 | 182 | 1 117 695 | 587 377 | 525 717 | 2930 | 60.47 | 97.19 | 94.01 | 0.57 |
| 10/28/2021 | 697 | 102 | 390 | 199 | 1 118 392 | 587 479 | 526 107 | 3129 | 60.50 | 97.21 | 94.08 | 0.61 |
| 10/29/2021 | 472 | 56 | 284 | 128 | 1 118 864 | 587 535 | 526 391 | 3257 | 60.53 | 97.22 | 94.13 | 0.63 |
| 10/30/2021 | 103 | 2 | 72 | 27 | 1 118 967 | 587 537 | 526 463 | 3284 | 60.53 | 97.22 | 94.14 | 0.64 |
| 10/31/2021 | 20 | 0 | 15 | 5 | 1 118 987 | 587 537 | 526 478 | 3289 | 60.54 | 97.22 | 94.15 | 0.64 |
| 11/1/2021 | 61 | 0 | 49 | 12 | 1 119 048 | 587 537 | 526 527 | 3301 | 60.54 | 97.22 | 94.16 | 0.64 |
| 11/2/2021 | 18 | 1 | 16 | 1 | 1 119 066 | 587 538 | 526 543 | 3302 | 60.54 | 97.22 | 94.16 | 0.64 |
| 11/3/2021 | 735 | 56 | 475 | 198 | 1 119 801 | 587 594 | 527 018 | 3500 | 60.58 | 97.23 | 94.24 | 0.68 |
| 11/4/2021 | 553 | 41 | 362 | 139 | 1 120 354 | 587 635 | 527 380 | 3639 | 60.61 | 97.24 | 94.31 | 0.71 |
| 11/5/2021 | 576 | 106 | 313 | 148 | 1 120 930 | 587 741 | 527 693 | 3787 | 60.64 | 97.26 | 94.36 | 0.74 |
| 11/6/2021 | 180 | 51 | 95 | 34 | 1 121 110 | 587 792 | 527 788 | 3821 | 60.65 | 97.26 | 94.38 | 0.74 |
| 11/7/2021 | 249 | 4 | 20 | 225 | 1 121 359 | 587 796 | 527 808 | 4046 | 60.66 | 97.26 | 94.38 | 0.79 |
| 11/8/2021 | 484 | 35 | 306 | 139 | 1 121 843 | 587 831 | 528 114 | 4185 | 60.69 | 97.27 | 94.44 | 0.82 |
| 11/9/2021 | 684 | 58 | 384 | 223 | 1 122 527 | 587 889 | 528 498 | 4408 | 60.73 | 97.28 | 94.51 | 0.86 |
| 11/10/2021 | 665 | 69 | 309 | 279 | 1 123 192 | 587 958 | 528 807 | 4687 | 60.76 | 97.29 | 94.56 | 0.91 |
| 11/11/2021 | 806 | 54 | 516 | 228 | 1 123 998 | 588 012 | 529 323 | 4915 | 60.81 | 97.30 | 94.66 | 0.96 |
| 11/12/2021 | 695 | 48 | 313 | 328 | 1 124 693 | 588 060 | 529 636 | 5243 | 60.84 | 97.31 | 94.71 | 1.02 |
| 11/13/2021 | 145 | 36 | 57 | 47 | 1 124 838 | 588 096 | 529 693 | 5290 | 60.85 | 97.31 | 94.72 | 1.03 |
| 11/14/2021 | 18 | 4 | 6 | 8 | 1 124 856 | 588 100 | 529 699 | 5298 | 60.85 | 97.31 | 94.72 | 1.03 |
| 11/15/2021 | 142 | 3 | 70 | 67 | 1 124 998 | 588 103 | 529 769 | 5365 | 60.86 | 97.32 | 94.74 | 1.05 |
| 11/16/2021 | 903 | 50 | 331 | 495 | 1 125 901 | 588 153 | 530 100 | 5860 | 60.91 | 97.32 | 94.79 | 1.14 |
| 11/17/2021 | 1437 | 58 | 431 | 937 | 1 127 338 | 588 211 | 530 531 | 6797 | 60.99 | 97.33 | 94.87 | 1.32 |
| 11/18/2021 | 1210 | 35 | 458 | 647 | 1 128 548 | 588 246 | 530 989 | 7444 | 61.05 | 97.34 | 94.95 | 1.45 |
| 11/19/2021 | 1010 | 16 | 204 | 761 | 1 129 558 | 588 262 | 531 193 | 8205 | 61.11 | 97.34 | 94.99 | 1.60 |
| 11/20/2021 | 314 | 22 | 95 | 186 | 1 129 872 | 588 284 | 531 288 | 8391 | 61.12 | 97.34 | 95.01 | 1.64 |
| 11/21/2021 | 88 | 1 | 14 | 72 | 1 129 960 | 588 285 | 531 302 | 8463 | 61.13 | 97.35 | 95.01 | 1.65 |
| 11/22/2021 | 2392 | 66 | 312 | 1993 | 1 132 352 | 588 351 | 531 614 | 10 456 | 61.26 | 97.36 | 95.07 | 2.04 |
| 11/23/2021 | 2591 | 20 | 370 | 2139 | 1 134 943 | 588 371 | 531 984 | 12 595 | 61.40 | 97.36 | 95.13 | 2.46 |
| 11/24/2021 | 3264 | 47 | 338 | 2694 | 1 138 207 | 588 418 | 532 322 | 15 289 | 61.57 | 97.37 | 95.19 | 2.98 |
| 11/25/2021 | 3162 | 127 | 511 | 2430 | 1 141 369 | 588 545 | 532 833 | 17 719 | 61.75 | 97.39 | 95.28 | 3.45 |
| 11/26/2021 | 2952 | 9 | 252 | 2636 | 1 144 321 | 588 554 | 533 085 | 20 355 | 61.91 | 97.39 | 95.33 | 3.97 |
| 11/27/2021 | 1392 | 20 | 102 | 1106 | 1 145 713 | 588 574 | 533 187 | 21 461 | 61.98 | 97.39 | 95.35 | 4.18 |
| 11/28/2021 | 121 | 0 | 22 | 98 | 1 145 834 | 588 574 | 533 209 | 21 559 | 61.99 | 97.39 | 95.35 | 4.20 |
| 11/29/2021 | 3584 | 35 | 383 | 3031 | 1 149 418 | 588 609 | 533 592 | 24 590 | 62.18 | 97.4 | 95.42 | 4.79 |
| 11/30/2021 | 4357 | 75 | 348 | 3633 | 1 153 775 | 588 684 | 533 940 | 28 223 | 62.42 | 97.41 | 95.48 | 5.50 |
| 12/1/2021 | 5178 | 47 | 284 | 4749 | 1 158 953 | 588 731 | 534 224 | 32 972 | 62.70 | 97.42 | 95.53 | 6.43 |
| 12/2/2021 | 5740 | 54 | 364 | 5231 | 1 164 693 | 588 785 | 534 588 | 38 203 | 63.01 | 97.43 | 95.60 | 7.45 |
| 12/3/2021 | 4396 | 37 | 195 | 4033 | 1 169 089 | 588 822 | 534 783 | 42 236 | 63.25 | 97.43 | 95.63 | 8.23 |
| 12/4/2021 | 1792 | 1 | 75 | 1521 | 1 170 881 | 588 823 | 534 858 | 43 757 | 63.34 | 97.43 | 95.65 | 8.53 |
| 12/5/2021 | 118 | 3 | 17 | 97 | 1 170 999 | 588 826 | 534 875 | 43 854 | 63.35 | 97.43 | 95.65 | 8.55 |
| 12/6/2021 | 5614 | 40 | 371 | 5034 | 1 176 613 | 588 866 | 535 246 | 48 888 | 63.65 | 97.44 | 95.71 | 9.53 |
| 12/7/2021 | 7553 | 41 | 520 | 6546 | 1 184 166 | 588 907 | 535 766 | 55 434 | 64.06 | 97.45 | 95.81 | 10.81 |
| 12/8/2021 | 6719 | 39 | 367 | 6020 | 1 190 885 | 588 946 | 536 133 | 61 454 | 64.42 | 97.45 | 95.87 | 11.98 |
| 12/9/2021 | 8809 | 161 | 584 | 7776 | 1 199 694 | 589 107 | 536 717 | 69 230 | 64.90 | 97.48 | 95.98 | 13.49 |
| 12/10/2021 | 8502 | 111 | 445 | 7672 | 1 208 196 | 589 218 | 537 162 | 76 902 | 65.36 | 97.50 | 96.06 | 14.99 |
| 12/11/2021 | 1582 | 15 | 147 | 1282 | 1 209 778 | 589 233 | 537 309 | 78 184 | 65.45 | 97.50 | 96.08 | 15.24 |
| 12/12/2021 | 175 | 6 | 11 | 155 | 1 209 953 | 589 239 | 537 320 | 78 339 | 65.46 | 97.50 | 96.09 | 15.27 |
| 12/13/2021 | 7381 | 56 | 348 | 6589 | 1 217 334 | 589 295 | 537 668 | 84 928 | 65.86 | 97.51 | 96.15 | 16.55 |
| 12/14/2021 | 9186 | 62 | 371 | 8323 | 1 226 520 | 589 357 | 538 039 | 93 251 | 66.35 | 97.52 | 96.21 | 18.18 |
| 12/15/2021 | 9514 | 105 | 450 | 8511 | 1 236 034 | 589 462 | 538 489 | 101 762 | 66.87 | 97.54 | 96.29 | 19.84 |
| 12/16/2021 | 10 771 | 71 | 303 | 9808 | 1 246 805 | 589 533 | 538 792 | 111 570 | 67.45 | 97.55 | 96.35 | 21.75 |
| 12/17/2021 | 7494 | 32 | 249 | 6737 | 1 254 299 | 589 565 | 539 041 | 118 307 | 67.86 | 97.56 | 96.39 | 23.06 |
| 12/18/2021 | 1380 | 3 | 50 | 1284 | 1 255 679 | 589 568 | 539 091 | 119 591 | 67.93 | 97.56 | 96.40 | 23.31 |
| 12/19/2021 | 116 | 0 | 3 | 113 | 1 255 795 | 589 568 | 539 094 | 119 704 | 67.94 | 97.56 | 96.40 | 23.33 |
| 12/20/2021 | 8700 | 49 | 293 | 8081 | 1 264 495 | 589 617 | 539 387 | 127 785 | 68.41 | 97.57 | 96.46 | 24.91 |
| 12/21/2021 | 7266 | 33 | 232 | 6699 | 1 271 761 | 589 650 | 539 619 | 134 484 | 68.80 | 97.57 | 96.50 | 26.21 |
| 12/22/2021 | 7107 | 22 | 207 | 6611 | 1 278 868 | 589 672 | 539 826 | 141 095 | 69.18 | 97.57 | 96.53 | 27.50 |
| 12/23/2021 | 4652 | 36 | 127 | 4234 | 1 283 520 | 589 708 | 539 953 | 145 329 | 69.44 | 97.58 | 96.56 | 28.33 |
| 12/24/2021 | 225 | 0 | 9 | 214 | 1 283 745 | 589 708 | 539 962 | 145 543 | 69.45 | 97.58 | 96.56 | 28.37 |
| 12/25/2021 | 8 | 0 | 1 | 5 | 1 283 753 | 589 708 | 539 963 | 145 548 | 69.45 | 97.58 | 96.56 | 28.37 |
| 12/26/2021 | 2 | 1 | 0 | 1 | 1 283 755 | 589 709 | 539 963 | 145 549 | 69.45 | 97.58 | 96.56 | 28.37 |
| 12/27/2021 | 3586 | 12 | 75 | 3363 | 1 287 341 | 589 721 | 540 038 | 148 912 | 69.64 | 97.58 | 96.57 | 29.03 |
| 12/28/2021 | 4145 | 23 | 126 | 3852 | 1 291 486 | 589 744 | 540 164 | 152 764 | 69.87 | 97.59 | 96.59 | 29.78 |
| 12/29/2021 | 3841 | 11 | 93 | 3489 | 1 295 327 | 589 755 | 540 257 | 156 253 | 70.07 | 97.59 | 96.61 | 30.46 |
| 12/30/2021 | 1171 | 4 | 38 | 1056 | 1 296 498 | 589 759 | 540 295 | 157 309 | 70.14 | 97.59 | 96.62 | 30.66 |
| 12/31/2021 | 50 | 0 | 2 | 44 | 1 296 548 | 589 759 | 540 297 | 157 353 | 70.14 | 97.59 | 96.62 | 30.67 |
| 1/1/2022 | 20 | 1 | 1 | 16 | 1 296 568 | 589 760 | 540 298 | 157 369 | 70.14 | 97.59 | 96.62 | 30.67 |
| 1/2/2022 | 18 | 0 | 1 | 17 | 1 296 586 | 589 760 | 540 299 | 157 386 | 70.14 | 97.59 | 96.62 | 30.68 |
| 1/3/2022 | 1632 | 10 | 67 | 1506 | 1 298 218 | 589 770 | 540 366 | 158 892 | 70.23 | 97.59 | 96.63 | 30.97 |
| 1/4/2022 | 2914 | 36 | 75 | 2702 | 1 301 132 | 589 806 | 540 441 | 161 594 | 70.39 | 97.60 | 96.64 | 31.50 |
| 1/5/2022 | 4263 | 9 | 126 | 3988 | 1 305 395 | 589 815 | 540 567 | 165 582 | 70.62 | 97.60 | 96.67 | 32.28 |
| 1/6/2022 | 4785 | 17 | 115 | 4403 | 1 310 180 | 589 832 | 540 682 | 169 985 | 70.88 | 97.60 | 96.69 | 33.13 |
| 1/7/2022 | 3539 | 7 | 95 | 3202 | 1 313 719 | 589 839 | 540 777 | 173 187 | 71.07 | 97.60 | 96.70 | 33.76 |
| 1/8/2022 | 415 | 3 | 18 | 374 | 1 314 134 | 589 842 | 540 795 | 173 561 | 71.09 | 97.60 | 96.71 | 33.83 |
| 1/9/2022 | 165 | 0 | 0 | 165 | 1 314 299 | 589 842 | 540 795 | 173 726 | 71.10 | 97.60 | 96.71 | 33.86 |
| 1/10/2022 | 4059 | 42 | 135 | 3656 | 1 318 358 | 589 884 | 540 930 | 177 382 | 71.32 | 97.61 | 96.73 | 34.58 |
| 1/11/2022 | 5191 | 50 | 135 | 4699 | 1 323 549 | 589 934 | 541 065 | 182 081 | 71.60 | 97.62 | 96.76 | 35.49 |
| 1/12/2022 | 5510 | 52 | 169 | 5015 | 1 329 059 | 589 986 | 541 234 | 187 096 | 71.90 | 97.63 | 96.79 | 36.47 |
| 1/13/2022 | 6051 | 39 | 196 | 5472 | 1 335 110 | 590 025 | 541 430 | 192 568 | 72.23 | 97.63 | 96.82 | 37.54 |
| 1/14/2022 | 3795 | 16 | 128 | 3427 | 1 338 905 | 590 041 | 541 558 | 195 995 | 72.43 | 97.64 | 96.84 | 38.20 |
| 1/15/2022 | 567 | 1 | 12 | 535 | 1 339 472 | 590 042 | 541 570 | 196 530 | 72.46 | 97.64 | 96.85 | 38.31 |
| 1/16/2022 | 107 | 0 | 1 | 106 | 1 339 579 | 590 042 | 541 571 | 196 636 | 72.47 | 97.64 | 96.85 | 38.33 |
| 1/17/2022 | 4202 | 151 | 133 | 3744 | 1 343 781 | 590 193 | 541 704 | 200 380 | 72.70 | 97.66 | 96.87 | 39.06 |
| 1/18/2022 | 5403 | 232 | 163 | 4757 | 1 349 184 | 590 425 | 541 867 | 205 137 | 72.99 | 97.70 | 96.90 | 39.99 |
| 1/19/2022 | 5688 | 452 | 170 | 4829 | 1 354 872 | 590 877 | 542 037 | 209 966 | 73.30 | 97.77 | 96.93 | 40.93 |
| 1/20/2022 | 5192 | 488 | 222 | 4203 | 1 360 064 | 591 365 | 542 259 | 214 169 | 73.58 | 97.85 | 96.97 | 41.75 |
| 1/21/2022 | 3745 | 493 | 81 | 3016 | 1 363 809 | 591 858 | 542 340 | 217 185 | 73.78 | 97.94 | 96.98 | 42.33 |
| 1/22/2022 | 699 | 114 | 18 | 499 | 1 364 508 | 591 972 | 542 358 | 217 684 | 73.82 | 97.96 | 96.99 | 42.43 |
| 1/23/2022 | 70 | 11 | 17 | 41 | 1 364 578 | 591 983 | 542 375 | 217 725 | 73.82 | 97.96 | 96.99 | 42.44 |
| 1/24/2022 | 4115 | 520 | 137 | 3306 | 1 368 693 | 592 503 | 542 512 | 221 031 | 74.04 | 98.04 | 97.01 | 43.08 |
| 1/25/2022 | 4556 | 560 | 108 | 3804 | 1 373 249 | 593 063 | 542 620 | 224 835 | 74.29 | 98.14 | 97.03 | 43.82 |
| 1/26/2022 | 5216 | 710 | 99 | 4231 | 1 378 465 | 593 773 | 542 719 | 229 066 | 74.57 | 98.25 | 97.05 | 44.65 |
| 1/27/2022 | 4278 | 383 | 102 | 3550 | 1 382 743 | 594 156 | 542 821 | 232 616 | 74.80 | 98.32 | 97.07 | 45.34 |
| 1/28/2022 | 3151 | 504 | 156 | 2375 | 1 385 894 | 594 660 | 542 977 | 234 991 | 74.97 | 98.40 | 97.10 | 45.80 |
| 1/29/2022 | 700 | 13 | 10 | 665 | 1 386 594 | 594 673 | 542 987 | 235 656 | 75.01 | 98.40 | 97.10 | 45.93 |
| 1/30/2022 | 143 | 0 | 15 | 126 | 1 386 737 | 594 673 | 543 002 | 235 782 | 75.02 | 98.40 | 97.10 | 45.96 |
| 1/31/2022 | 3437 | 284 | 139 | 2934 | 1 390 174 | 594 957 | 543 141 | 238 716 | 75.21 | 98.45 | 97.13 | 46.53 |
| 2/1/2022 | 4057 | 617 | 120 | 3195 | 1 394 231 | 595 574 | 543 261 | 241 911 | 75.43 | 98.55 | 97.15 | 47.15 |
| 2/2/2022 | 4161 | 353 | 166 | 3502 | 1 398 392 | 595 927 | 543 427 | 245 413 | 75.65 | 98.61 | 97.18 | 47.84 |
| 2/3/2022 | 4900 | 427 | 155 | 3847 | 1 403 292 | 596 354 | 543 582 | 249 260 | 75.92 | 98.68 | 97.21 | 48.59 |
| 2/4/2022 | 3330 | 475 | 59 | 2623 | 1 406 622 | 596 829 | 543 641 | 251 883 | 76.10 | 98.76 | 97.22 | 49.10 |
| 2/5/2022 | 1221 | 130 | 21 | 1062 | 1 407 843 | 596 959 | 543 662 | 252 945 | 76.16 | 98.78 | 97.22 | 49.30 |
| 2/6/2022 | 345 | 14 | 4 | 325 | 1 408 188 | 596 973 | 543 666 | 253 270 | 76.18 | 98.78 | 97.22 | 49.37 |
| 2/7/2022 | 3362 | 326 | 106 | 2860 | 1 411 550 | 597 299 | 543 772 | 256 130 | 76.36 | 98.84 | 97.24 | 49.92 |
| 2/8/2022 | 4197 | 400 | 104 | 3510 | 1 415 747 | 597 699 | 543 876 | 259 640 | 76.59 | 98.90 | 97.26 | 50.61 |
| 2/9/2022 | 4736 | 336 | 130 | 4173 | 1 420 483 | 598 035 | 544 006 | 263 813 | 76.85 | 98.96 | 97.28 | 51.42 |
| 2/10/2022 | 4504 | 256 | 140 | 3939 | 1 424 987 | 598 291 | 544 146 | 267 752 | 77.09 | 99.00 | 97.31 | 52.19 |
| 2/11/2022 | 3085 | 231 | 178 | 2518 | 1 428 072 | 598 522 | 544 324 | 270 270 | 77.26 | 99.04 | 97.34 | 52.68 |
| 2/12/2022 | 1276 | 21 | 32 | 1209 | 1 429 348 | 598 543 | 544 356 | 271 479 | 77.33 | 99.04 | 97.34 | 52.92 |
| 2/13/2022 | 425 | 4 | 7 | 404 | 1 429 773 | 598 547 | 544 363 | 271 883 | 77.35 | 99.04 | 97.35 | 53.00 |
| 2/14/2022 | 3858 | 124 | 112 | 3519 | 1 433 631 | 598 671 | 544 475 | 275 402 | 77.56 | 99.06 | 97.37 | 53.68 |
| 2/15/2022 | 3663 | 212 | 127 | 3183 | 1 437 294 | 598 883 | 544 602 | 278 585 | 77.75 | 99.10 | 97.39 | 54.30 |
| 2/16/2022 | 3443 | 125 | 129 | 2926 | 1 440 737 | 599 008 | 544 731 | 281 511 | 77.94 | 99.12 | 97.41 | 54.87 |
| 2/17/2022 | 3543 | 391 | 104 | 2781 | 1 444 280 | 599 399 | 544 835 | 284 292 | 78.13 | 99.18 | 97.43 | 55.41 |
| 2/18/2022 | 2279 | 85 | 74 | 2048 | 1 446 559 | 599 484 | 544 909 | 286 340 | 78.26 | 99.20 | 97.44 | 55.81 |
| 2/19/2022 | 834 | 240 | 4 | 549 | 1 447 393 | 599 724 | 544 913 | 286 889 | 78.30 | 99.24 | 97.44 | 55.92 |
| 2/20/2022 | 148 | 1 | 1 | 141 | 1 447 541 | 599 725 | 544 914 | 287 030 | 78.31 | 99.24 | 97.44 | 55.95 |
| 2/21/2022 | 2400 | 96 | 91 | 2065 | 1 449 941 | 599 821 | 545 005 | 289 095 | 78.44 | 99.25 | 97.46 | 56.35 |
| 2/22/2022 | 3092 | 116 | 132 | 2686 | 1 453 033 | 599 937 | 545 137 | 291 781 | 78.61 | 99.27 | 97.48 | 56.87 |
| 2/23/2022 | 3454 | 157 | 122 | 3026 | 1 456 487 | 600 094 | 545 259 | 294 807 | 78.79 | 99.30 | 97.51 | 57.46 |
| 2/24/2022 | 2758 | 120 | 113 | 2339 | 1 459 245 | 600 214 | 545 372 | 297 146 | 78.94 | 99.32 | 97.53 | 57.92 |
| 2/25/2022 | 2266 | 73 | 111 | 1943 | 1 461 511 | 600 287 | 545 483 | 299 089 | 79.06 | 99.33 | 97.55 | 58.30 |
| 2/26/2022 | 166 | 4 | 18 | 137 | 1 461 677 | 600 291 | 545 501 | 299 226 | 79.07 | 99.33 | 97.55 | 58.32 |
| 2/27/2022 | 142 | 60 | 15 | 60 | 1 461 819 | 600 351 | 545 516 | 299 286 | 79.08 | 99.34 | 97.55 | 58.34 |
| 2/28/2022 | 375 | 30 | 12 | 320 | 1 462 194 | 600 381 | 545 528 | 299 606 | 79.10 | 99.35 | 97.55 | 58.40 |
| 3/1/2022 | 432 | 36 | 31 | 359 | 1 462 626 | 600 417 | 545 559 | 299 965 | 79.13 | 99.35 | 97.56 | 58.47 |
| 3/2/2022 | 677 | 54 | 14 | 554 | 1 463 303 | 600 471 | 545 573 | 300 519 | 79.16 | 99.36 | 97.56 | 58.58 |
| 3/3/2022 | 2057 | 32 | 112 | 1736 | 1 465 360 | 600 503 | 545 685 | 302 255 | 79.27 | 99.37 | 97.58 | 58.92 |
| 3/4/2022 | 1320 | 29 | 83 | 1149 | 1 466 680 | 600 532 | 545 768 | 303 404 | 79.34 | 99.37 | 97.60 | 59.14 |
| 3/5/2022 | 702 | 10 | 43 | 403 | 1 467 382 | 600 542 | 545 811 | 303 807 | 79.38 | 99.37 | 97.60 | 59.22 |
| 3/6/2022 | 17 | 0 | 0 | 17 | 1 467 399 | 600 542 | 545 811 | 303 824 | 79.38 | 99.37 | 97.60 | 59.22 |
| 3/7/2022 | 1702 | 29 | 156 | 1435 | 1 469 101 | 600 571 | 545 967 | 305 259 | 79.48 | 99.38 | 97.63 | 59.50 |
| 3/8/2022 | 2029 | 78 | 159 | 1705 | 1 471 130 | 600 649 | 546 126 | 306 964 | 79.59 | 99.39 | 97.66 | 59.83 |
| 3/9/2022 | 2320 | 96 | 106 | 1989 | 1 473 450 | 600 745 | 546 232 | 308 953 | 79.71 | 99.41 | 97.68 | 60.22 |
| 3/10/2022 | 2317 | 93 | 180 | 1911 | 1 475 767 | 600 838 | 546 412 | 310 864 | 79.84 | 99.42 | 97.71 | 60.59 |
| 3/11/2022 | 1421 | 36 | 104 | 1173 | 1 477 188 | 600 874 | 546 516 | 312 037 | 79.91 | 99.43 | 97.73 | 60.82 |
| 3/12/2022 | 262 | 4 | 10 | 237 | 1 477 450 | 600 878 | 546 526 | 312 274 | 79.93 | 99.43 | 97.73 | 60.87 |
| 3/13/2022 | 86 | 0 | 2 | 81 | 1 477 536 | 600 878 | 546 528 | 312 355 | 79.93 | 99.43 | 97.73 | 60.88 |
| 3/14/2022 | 1422 | 31 | 78 | 1267 | 1 478 958 | 600 909 | 546 606 | 313 622 | 80.01 | 99.43 | 97.75 | 61.13 |
| 3/15/2022 | 2167 | 70 | 157 | 1878 | 1 481 125 | 600 979 | 546 763 | 315 500 | 80.13 | 99.45 | 97.77 | 61.50 |
| 3/16/2022 | 2205 | 31 | 164 | 1768 | 1 483 330 | 601 010 | 546 927 | 317 268 | 80.25 | 99.45 | 97.80 | 61.84 |
| 3/17/2022 | 1823 | 20 | 145 | 1566 | 1 485 153 | 601 030 | 547 072 | 318 834 | 80.34 | 99.45 | 97.83 | 62.15 |
| 3/18/2022 | 1666 | 37 | 187 | 1340 | 1 486 819 | 601 067 | 547 259 | 320 174 | 80.43 | 99.46 | 97.86 | 62.41 |
| 3/19/2022 | 427 | 7 | 49 | 362 | 1 487 246 | 601 074 | 547 308 | 320 536 | 80.46 | 99.46 | 97.87 | 62.48 |
| 3/20/2022 | 79 | 0 | 3 | 58 | 1 487 325 | 601 074 | 547 311 | 320 594 | 80.46 | 99.46 | 97.87 | 62.49 |
| 3/21/2022 | 1887 | 25 | 297 | 1433 | 1 489 212 | 601 099 | 547 608 | 322 027 | 80.56 | 99.47 | 97.93 | 62.77 |
| 3/22/2022 | 2291 | 67 | 407 | 1697 | 1 491 503 | 601 166 | 548 015 | 323 724 | 80.69 | 99.48 | 98.00 | 63.10 |
| 3/23/2022 | 2555 | 26 | 355 | 2021 | 1 494 058 | 601 192 | 548 370 | 325 745 | 80.83 | 99.48 | 98.06 | 63.49 |
| 3/24/2022 | 1915 | 55 | 367 | 1437 | 1 495 973 | 601 247 | 548 737 | 327 182 | 80.93 | 99.49 | 98.13 | 63.77 |
| 3/25/2022 | 1631 | 29 | 207 | 1257 | 1 497 604 | 601 276 | 548 944 | 328 439 | 81.02 | 99.49 | 98.16 | 64.02 |
| 3/26/2022 | 222 | 3 | 54 | 149 | 1 497 826 | 601 279 | 548 998 | 328 588 | 81.03 | 99.50 | 98.17 | 64.05 |
| 3/27/2022 | 24 | 0 | 5 | 15 | 1 497 850 | 601 279 | 549 003 | 328 603 | 81.03 | 99.50 | 98.17 | 64.05 |
| 3/28/2022 | 1625 | 25 | 375 | 1143 | 1 499 475 | 601 304 | 549 378 | 329 746 | 81.12 | 99.50 | 98.24 | 64.27 |
| 3/29/2022 | 1825 | 65 | 318 | 1375 | 1 501 300 | 601 369 | 549 696 | 331 121 | 81.22 | 99.51 | 98.30 | 64.54 |
| 3/30/2022 | 1957 | 54 | 344 | 1458 | 1 503 257 | 601 423 | 550 040 | 332 579 | 81.32 | 99.52 | 98.36 | 64.83 |
| 3/31/2022 | 1546 | 42 | 267 | 1135 | 1 504 803 | 601 465 | 550 307 | 333 714 | 81.41 | 99.53 | 98.41 | 65.05 |
| 4/1/2022 | 959 | 41 | 253 | 600 | 1 505 762 | 601 506 | 550 560 | 334 314 | 81.46 | 99.53 | 98.45 | 65.16 |
| 4/2/2022 | 171 | 2 | 35 | 102 | 1 505 933 | 601 508 | 550 595 | 334 416 | 81.47 | 99.53 | 98.46 | 65.18 |
| 4/3/2022 | 22 | 0 | 2 | 11 | 1 505 955 | 601 508 | 550 597 | 334 427 | 81.47 | 99.53 | 98.46 | 65.19 |
| 4/4/2022 | 880 | 9 | 128 | 660 | 1 506 835 | 601 517 | 550 725 | 335 087 | 81.52 | 99.53 | 98.48 | 65.31 |
| 4/5/2022 | 1294 | 60 | 168 | 913 | 1 508 129 | 601 577 | 550 893 | 336 000 | 81.59 | 99.54 | 98.51 | 65.49 |
| 4/6/2022 | 1207 | 47 | 223 | 757 | 1 509 336 | 601 624 | 551 116 | 336 757 | 81.65 | 99.55 | 98.55 | 65.64 |
| 4/7/2022 | 951 | 13 | 112 | 681 | 1 510 287 | 601 637 | 551 228 | 337 438 | 81.70 | 99.55 | 98.57 | 65.77 |
| 4/8/2022 | 918 | 8 | 160 | 554 | 1 511 205 | 601 645 | 551 388 | 337 992 | 81.75 | 99.56 | 98.60 | 65.88 |
| 4/9/2022 | 140 | 3 | 13 | 107 | 1 511 345 | 601 648 | 551 401 | 338 099 | 81.76 | 99.56 | 98.60 | 65.90 |
| 4/10/2022 | 22 | 0 | 3 | 17 | 1 511 367 | 601 648 | 551 404 | 338 116 | 81.76 | 99.56 | 98.60 | 65.91 |
| 4/11/2022 | 954 | 15 | 134 | 672 | 1 512 321 | 601 663 | 551 538 | 338 788 | 81.81 | 99.56 | 98.63 | 66.04 |
| 4/12/2022 | 1097 | 12 | 176 | 703 | 1 513 418 | 601 675 | 551 714 | 339 491 | 81.87 | 99.56 | 98.66 | 66.17 |
| 4/13/2022 | 807 | 16 | 117 | 500 | 1 514 225 | 601 691 | 551 831 | 339 991 | 81.92 | 99.56 | 98.68 | 66.27 |
| 4/14/2022 | 237 | 10 | 41 | 122 | 1 514 462 | 601 701 | 551 872 | 340 113 | 81.93 | 99.57 | 98.69 | 66.29 |
| 4/15/2022 | 13 | 0 | 0 | 0 | 1 514 475 | 601 701 | 551 872 | 340 113 | 81.93 | 99.57 | 98.69 | 66.29 |
| 4/16/2022 | 7 | 0 | 0 | 6 | 1 514 482 | 601 701 | 551 872 | 340 119 | 81.93 | 99.57 | 98.69 | 66.30 |
| 4/17/2022 | 4 | 0 | 0 | 4 | 1 514 486 | 601 701 | 551 872 | 340 123 | 81.93 | 99.57 | 98.69 | 66.30 |
| 4/18/2022 | 853 | 6 | 150 | 526 | 1 515 339 | 601 707 | 552 022 | 340 649 | 81.98 | 99.57 | 98.71 | 66.40 |
| 4/19/2022 | 927 | 6 | 196 | 519 | 1 516 266 | 601 713 | 552 218 | 341 168 | 82.03 | 99.57 | 98.75 | 66.50 |
| 4/20/2022 | 1158 | 11 | 189 | 708 | 1 517 424 | 601 724 | 552 407 | 341 876 | 82.09 | 99.57 | 98.78 | 66.64 |
| 4/21/2022 | 111 | 0 | 7 | 87 | 1 517 535 | 601 724 | 552 414 | 341 963 | 82.10 | 99.57 | 98.78 | 66.66 |
| 4/22/2022 | 234 | 0 | 23 | 170 | 1 517 769 | 601 724 | 552 437 | 342 133 | 82.11 | 99.57 | 98.79 | 66.69 |
| 4/23/2022 | 248 | 0 | 3 | 58 | 1 518 017 | 601 724 | 552 440 | 342 191 | 82.12 | 99.57 | 98.79 | 66.70 |
| 4/24/2022 | 30 | 1 | 1 | 25 | 1 518 047 | 601 725 | 552 441 | 342 216 | 82.12 | 99.57 | 98.79 | 66.70 |
| 4/25/2022 | 911 | 1 | 129 | 568 | 1 518 958 | 601 726 | 552 570 | 342 784 | 82.17 | 99.57 | 98.81 | 66.82 |
| 4/26/2022 | 1218 | 74 | 147 | 791 | 1 520 176 | 601 800 | 552 717 | 343 575 | 82.24 | 99.58 | 98.84 | 66.97 |
| 4/27/2022 | 1125 | 5 | 223 | 702 | 1 521 301 | 601 805 | 552 940 | 344 277 | 82.30 | 99.58 | 98.88 | 67.11 |
| 4/28/2022 | 1130 | 16 | 175 | 714 | 1 522 431 | 601 821 | 553 115 | 344 991 | 82.36 | 99.58 | 98.91 | 67.25 |
| 4/29/2022 | 536 | 3 | 60 | 283 | 1 522 967 | 601 824 | 553 175 | 345 274 | 82.39 | 99.59 | 98.92 | 67.30 |
| 4/30/2022 | 664 | 3 | 79 | 254 | 1 523 631 | 601 827 | 553 254 | 345 528 | 82.43 | 99.59 | 98.94 | 67.35 |
| 5/1/2022 | 6 | 0 | 0 | 3 | 1 523 637 | 601 827 | 553 254 | 345 531 | 82.43 | 99.59 | 98.94 | 67.35 |
| 5/2/2022 | 616 | 5 | 78 | 314 | 1 524 253 | 601 832 | 553 332 | 345 845 | 82.46 | 99.59 | 98.95 | 67.41 |
| 5/3/2022 | 750 | 8 | 51 | 431 | 1 525 003 | 601 840 | 553 383 | 346 276 | 82.50 | 99.59 | 98.96 | 67.50 |
| 5/4/2022 | 1269 | 10 | 70 | 600 | 1 526 272 | 601 850 | 553 453 | 346 876 | 82.57 | 99.59 | 98.97 | 67.61 |
| 5/5/2022 | 1025 | 11 | 103 | 491 | 1 527 297 | 601 861 | 553 556 | 347 367 | 82.62 | 99.59 | 98.99 | 67.71 |
| 5/6/2022 | 643 | 11 | 34 | 334 | 1 527 940 | 601 872 | 553 590 | 347 701 | 82.66 | 99.59 | 99.00 | 67.77 |
| 5/7/2022 | 53 | 0 | 8 | 20 | 1 527 993 | 601 872 | 553 598 | 347 721 | 82.66 | 99.59 | 99.00 | 67.78 |
| 5/8/2022 | 9 | 0 | 0 | 4 | 1 528 002 | 601 872 | 553 598 | 347 725 | 82.66 | 99.59 | 99.00 | 67.78 |
| 5/9/2022 | 675 | 8 | 50 | 344 | 1 528 677 | 601 880 | 553 648 | 348 069 | 82.70 | 99.59 | 99.01 | 67.85 |
| 5/10/2022 | 963 | 7 | 65 | 364 | 1 529 640 | 601 887 | 553 713 | 348 433 | 82.75 | 99.60 | 99.02 | 67.92 |
| 5/11/2022 | 1113 | 14 | 210 | 382 | 1 530 753 | 601 901 | 553 923 | 348 815 | 82.81 | 99.60 | 99.05 | 67.99 |
| 5/12/2022 | 1044 | 6 | 83 | 418 | 1 531 797 | 601 907 | 554 006 | 349 233 | 82.87 | 99.60 | 99.07 | 68.07 |
| 5/13/2022 | 628 | 1 | 54 | 264 | 1 532 425 | 601 908 | 554 060 | 349 497 | 82.90 | 99.60 | 99.08 | 68.12 |
| 5/14/2022 | 94 | 0 | 28 | 22 | 1 532 519 | 601 908 | 554 088 | 349 519 | 82.91 | 99.60 | 99.08 | 68.13 |
| 5/15/2022 | 18 | 0 | 2 | 6 | 1 532 537 | 601 908 | 554 090 | 349 525 | 82.91 | 99.60 | 99.08 | 68.13 |
| 5/16/2022 | 591 | 3 | 51 | 283 | 1 533 128 | 601 911 | 554 141 | 349 808 | 82.94 | 99.60 | 99.09 | 68.18 |
| 5/17/2022 | 773 | 4 | 50 | 347 | 1 533 901 | 601 915 | 554 191 | 350 155 | 82.98 | 99.60 | 99.10 | 68.25 |
| 5/18/2022 | 901 | 13 | 44 | 327 | 1 534 802 | 601 928 | 554 235 | 350 482 | 83.03 | 99.60 | 99.11 | 68.32 |
| 5/19/2022 | 965 | 6 | 63 | 386 | 1 535 767 | 601 934 | 554 298 | 350 868 | 83.08 | 99.60 | 99.12 | 68.39 |
| 5/20/2022 | 597 | 2 | 50 | 248 | 1 536 364 | 601 936 | 554 348 | 351 116 | 83.11 | 99.60 | 99.13 | 68.44 |
| 5/21/2022 | 102 | 0 | 1 | 42 | 1 536 466 | 601 936 | 554 349 | 351 158 | 83.12 | 99.60 | 99.13 | 68.45 |
| 5/22/2022 | 1 | 0 | 0 | 0 | 1 536 467 | 601 936 | 554 349 | 351 158 | 83.12 | 99.60 | 99.13 | 68.45 |
| 5/23/2022 | 654 | 3 | 36 | 304 | 1 537 121 | 601 939 | 554 385 | 351 462 | 83.16 | 99.60 | 99.14 | 68.51 |
| 5/24/2022 | 1021 | 10 | 63 | 452 | 1 538 142 | 601 949 | 554 448 | 351 914 | 83.21 | 99.61 | 99.15 | 68.59 |
| 5/25/2022 | 1076 | 5 | 60 | 417 | 1 539 218 | 601 954 | 554 508 | 352 331 | 83.27 | 99.61 | 99.16 | 68.68 |
| 5/26/2022 | 943 | 7 | 120 | 336 | 1 540 161 | 601 961 | 554 628 | 352 667 | 83.32 | 99.61 | 99.18 | 68.74 |
| 5/27/2022 | 464 | 1 | 39 | 187 | 1 540 625 | 601 962 | 554 667 | 352 854 | 83.34 | 99.61 | 99.19 | 68.78 |
| 5/28/2022 | 290 | 2 | 14 | 55 | 1 540 915 | 601 964 | 554 681 | 352 909 | 83.36 | 99.61 | 99.19 | 68.79 |
| 5/29/2022 | 25 | 0 | 0 | 13 | 1 540 940 | 601 964 | 554 681 | 352 922 | 83.36 | 99.61 | 99.19 | 68.79 |
| 5/30/2022 | 697 | 5 | 56 | 272 | 1 541 637 | 601 969 | 554 737 | 353 194 | 83.40 | 99.61 | 99.20 | 68.84 |
| 5/31/2022 | 775 | 6 | 42 | 319 | 1 542 412 | 601 975 | 554 779 | 353 513 | 83.44 | 99.61 | 99.21 | 68.91 |
| 6/1/2022 | 989 | 4 | 56 | 378 | 1 543 401 | 601 979 | 554 835 | 353 891 | 83.50 | 99.61 | 99.22 | 68.98 |
| 6/2/2022 | 1027 | 8 | 27 | 397 | 1 544 428 | 601 987 | 554 862 | 354 288 | 83.55 | 99.61 | 99.22 | 69.06 |
| 6/3/2022 | 760 | 1 | 27 | 249 | 1 545 188 | 601 988 | 554 889 | 354 537 | 83.59 | 99.61 | 99.23 | 69.11 |
| 6/4/2022 | 189 | 2 | 21 | 69 | 1 545 377 | 601 990 | 554 910 | 354 606 | 83.60 | 99.61 | 99.23 | 69.12 |
| 6/5/2022 | 17 | 0 | 1 | 7 | 1 545 394 | 601 990 | 554 911 | 354 613 | 83.60 | 99.61 | 99.23 | 69.12 |
| 6/6/2022 | 876 | 3 | 42 | 304 | 1 546 270 | 601 993 | 554 953 | 354 917 | 83.65 | 99.61 | 99.24 | 69.18 |
| 6/7/2022 | 1225 | 8 | 46 | 335 | 1 547 495 | 602 001 | 554 999 | 355 252 | 83.72 | 99.61 | 99.25 | 69.25 |
| 6/8/2022 | 1557 | 11 | 55 | 543 | 1 549 052 | 602 012 | 555 054 | 355 795 | 83.80 | 99.62 | 99.26 | 69.35 |
| 6/9/2022 | 1609 | 11 | 42 | 386 | 1 550 661 | 602 023 | 555 096 | 356 181 | 83.89 | 99.62 | 99.26 | 69.43 |
| 6/10/2022 | 1369 | 0 | 30 | 307 | 1 552 030 | 602 023 | 555 126 | 356 488 | 83.96 | 99.62 | 99.27 | 69.49 |
| 6/11/2022 | 1125 | 0 | 2 | 65 | 1 553 155 | 602 023 | 555 128 | 356 553 | 84.02 | 99.62 | 99.27 | 69.50 |
| 6/12/2022 | 9 | 0 | 1 | 2 | 1 553 164 | 602 023 | 555 129 | 356 555 | 84.02 | 99.62 | 99.27 | 69.50 |
| 6/13/2022 | 1303 | 6 | 32 | 395 | 1 554 467 | 602 029 | 555 161 | 356 950 | 84.09 | 99.62 | 99.28 | 69.58 |
| 6/14/2022 | 1837 | 2 | 33 | 526 | 1 556 304 | 602 031 | 555 194 | 357 476 | 84.19 | 99.62 | 99.28 | 69.68 |
| 6/15/2022 | 2317 | 14 | 42 | 596 | 1 558 621 | 602 045 | 555 236 | 358 072 | 84.32 | 99.62 | 99.29 | 69.80 |
| 6/16/2022 | 279 | 0 | 0 | 32 | 1 558 900 | 602 045 | 555 236 | 358 104 | 84.33 | 99.62 | 99.29 | 69.80 |
| 6/17/2022 | 580 | 1 | 19 | 163 | 1 559 480 | 602 046 | 555 255 | 358 267 | 84.36 | 99.62 | 99.29 | 69.83 |
| 6/18/2022 | 173 | 0 | 3 | 39 | 1 559 653 | 602 046 | 555 258 | 358 306 | 84.37 | 99.62 | 99.29 | 69.84 |
| 6/19/2022 | 11 | 0 | 0 | 4 | 1 559 664 | 602 046 | 555 258 | 358 310 | 84.37 | 99.62 | 99.29 | 69.84 |
| 6/20/2022 | 1508 | 9 | 30 | 385 | 1 561 172 | 602 055 | 555 288 | 358 695 | 84.46 | 99.62 | 99.30 | 69.92 |
| 6/21/2022 | 2377 | 11 | 41 | 581 | 1 563 549 | 602 066 | 555 329 | 359 276 | 84.59 | 99.63 | 99.31 | 70.03 |
| 6/22/2022 | 2233 | 22 | 69 | 475 | 1 565 782 | 602 088 | 555 398 | 359 751 | 84.71 | 99.63 | 99.32 | 70.12 |
| 6/23/2022 | 1729 | 3 | 33 | 293 | 1 567 511 | 602 091 | 555 431 | 360 044 | 84.80 | 99.63 | 99.32 | 70.18 |
| 6/24/2022 | 1063 | 2 | 9 | 208 | 1 568 574 | 602 093 | 555 440 | 360 252 | 84.86 | 99.63 | 99.33 | 70.22 |
| 6/25/2022 | 442 | 0 | 7 | 100 | 1 569 016 | 602 093 | 555 447 | 360 352 | 84.88 | 99.63 | 99.33 | 70.24 |
| 6/26/2022 | 66 | 0 | 1 | 9 | 1 569 082 | 602 093 | 555 448 | 360 361 | 84.88 | 99.63 | 99.33 | 70.24 |
| 6/27/2022 | 1809 | 3 | 28 | 372 | 1 570 891 | 602 096 | 555 476 | 360 733 | 84.98 | 99.63 | 99.33 | 70.31 |
| 6/28/2022 | 2390 | 10 | 43 | 459 | 1 573 281 | 602 106 | 555 519 | 361 192 | 85.11 | 99.63 | 99.34 | 70.40 |
| 6/29/2022 | 1933 | 13 | 34 | 338 | 1 575 214 | 602 119 | 555 553 | 361 530 | 85.22 | 99.63 | 99.35 | 70.47 |
| 6/30/2022 | 2197 | 10 | 34 | 411 | 1 577 411 | 602 129 | 555 587 | 361 941 | 85.33 | 99.64 | 99.35 | 70.55 |
| 7/1/2022 | 1900 | 11 | 42 | 332 | 1 579 311 | 602 140 | 555 629 | 362 273 | 85.44 | 99.64 | 99.36 | 70.61 |
| 7/2/2022 | 347 | 2 | 5 | 45 | 1 579 658 | 602 142 | 555 634 | 362 318 | 85.46 | 99.64 | 99.36 | 70.62 |
| 7/3/2022 | 70 | 1 | 3 | 12 | 1 579 728 | 602 143 | 555 637 | 362 330 | 85.46 | 99.64 | 99.36 | 70.63 |
| 7/4/2022 | 1972 | 4 | 45 | 401 | 1 581 700 | 602 147 | 555 682 | 362 731 | 85.57 | 99.64 | 99.37 | 70.70 |
| 7/5/2022 | 2525 | 4 | 36 | 424 | 1 584 225 | 602 151 | 555 718 | 363 155 | 85.70 | 99.64 | 99.38 | 70.79 |
| 7/6/2022 | 3159 | 3 | 58 | 567 | 1 587 384 | 602 154 | 555 776 | 363 722 | 85.87 | 99.64 | 99.39 | 70.90 |
| 7/7/2022 | 3021 | 5 | 32 | 651 | 1 590 405 | 602 159 | 555 808 | 364 373 | 86.04 | 99.64 | 99.39 | 71.02 |
| 7/8/2022 | 2030 | 2 | 16 | 418 | 1 592 435 | 602 161 | 555 824 | 364 791 | 86.15 | 99.64 | 99.39 | 71.10 |
| 7/9/2022 | 199 | 0 | 1 | 22 | 1 592 634 | 602 161 | 555 825 | 364 813 | 86.16 | 99.64 | 99.39 | 71.11 |
| 7/10/2022 | 38 | 0 | 3 | 14 | 1 592 672 | 602 161 | 555 828 | 364 827 | 86.16 | 99.64 | 99.40 | 71.11 |
| 7/11/2022 | 2133 | 2 | 35 | 413 | 1 594 805 | 602 163 | 555 863 | 365 240 | 86.28 | 99.64 | 99.40 | 71.19 |
| 7/12/2022 | 3105 | 10 | 46 | 613 | 1 597 910 | 602 173 | 555 909 | 365 853 | 86.44 | 99.64 | 99.41 | 71.31 |
| 7/13/2022 | 3235 | 5 | 54 | 564 | 1 601 145 | 602 178 | 555 963 | 366 417 | 86.62 | 99.64 | 99.42 | 71.42 |
| 7/14/2022 | 2813 | 1 | 34 | 429 | 1 603 958 | 602 179 | 555 997 | 366 846 | 86.77 | 99.64 | 99.43 | 71.51 |
| 7/15/2022 | 2105 | 4 | 20 | 312 | 1 606 063 | 602 183 | 556 017 | 367 158 | 86.88 | 99.64 | 99.43 | 71.57 |
| 7/16/2022 | 206 | 1 | 5 | 36 | 1 606 269 | 602 184 | 556 022 | 367 194 | 86.90 | 99.65 | 99.43 | 71.57 |
| 7/17/2022 | 15 | 0 | 1 | 3 | 1 606 284 | 602 184 | 556 023 | 367 197 | 86.90 | 99.65 | 99.43 | 71.57 |
| 7/18/2022 | 2026 | 3 | 31 | 341 | 1 608 310 | 602 187 | 556 054 | 367 538 | 87.01 | 99.65 | 99.44 | 71.64 |
| 7/19/2022 | 3144 | 8 | 47 | 614 | 1 611 454 | 602 195 | 556 101 | 368 152 | 87.18 | 99.65 | 99.44 | 71.76 |
| 7/20/2022 | 3440 | 6 | 39 | 651 | 1 614 894 | 602 201 | 556 140 | 368 803 | 87.36 | 99.65 | 99.45 | 71.89 |
| 7/21/2022 | 2637 | 13 | 48 | 570 | 1 617 531 | 602 214 | 556 188 | 369 373 | 87.51 | 99.65 | 99.46 | 72.00 |
| 7/22/2022 | 1635 | 3 | 27 | 270 | 1 619 166 | 602 217 | 556 215 | 369 643 | 87.59 | 99.65 | 99.46 | 72.05 |
| 7/23/2022 | 403 | 2 | 4 | 73 | 1 619 569 | 602 219 | 556 219 | 369 716 | 87.62 | 99.65 | 99.47 | 72.06 |
| 7/24/2022 | 94 | 0 | 19 | 12 | 1 619 663 | 602 219 | 556 238 | 369 728 | 87.62 | 99.65 | 99.47 | 72.07 |
| 7/25/2022 | 2531 | 18 | 38 | 424 | 1 622 194 | 602 237 | 556 276 | 370 152 | 87.76 | 99.65 | 99.48 | 72.15 |
| 7/26/2022 | 3031 | 16 | 41 | 707 | 1 625 225 | 602 253 | 556 317 | 370 859 | 87.92 | 99.66 | 99.48 | 72.29 |
| 7/27/2022 | 2923 | 31 | 43 | 462 | 1 628 148 | 602 284 | 556 360 | 371 321 | 88.08 | 99.66 | 99.49 | 72.38 |
| 7/28/2022 | 2387 | 13 | 34 | 368 | 1 630 535 | 602 297 | 556 394 | 371 689 | 88.21 | 99.66 | 99.50 | 72.45 |
| 7/29/2022 | 1458 | 3 | 21 | 320 | 1 631 993 | 602 300 | 556 415 | 372 009 | 88.29 | 99.66 | 99.50 | 72.51 |
| 7/30/2022 | 246 | 2 | 8 | 35 | 1 632 239 | 602 302 | 556 423 | 372 044 | 88.30 | 99.66 | 99.50 | 72.52 |
| 7/31/2022 | 21 | 0 | 0 | 10 | 1 632 260 | 602 302 | 556 423 | 372 054 | 88.30 | 99.66 | 99.50 | 72.52 |
| 8/1/2022 | 1862 | 15 | 31 | 270 | 1 634 122 | 602 317 | 556 454 | 372 324 | 88.40 | 99.67 | 99.51 | 72.57 |
| 8/2/2022 | 2546 | 27 | 41 | 348 | 1 636 668 | 602 344 | 556 495 | 372 672 | 88.54 | 99.67 | 99.51 | 72.64 |
| 8/3/2022 | 2066 | 13 | 33 | 368 | 1 638 734 | 602 357 | 556 528 | 373 040 | 88.65 | 99.67 | 99.52 | 72.71 |
| 8/4/2022 | 2008 | 2 | 30 | 286 | 1 640 742 | 602 359 | 556 558 | 373 326 | 88.76 | 99.67 | 99.53 | 72.77 |
| 8/5/2022 | 1583 | 9 | 20 | 317 | 1 642 325 | 602 368 | 556 578 | 373 643 | 88.85 | 99.68 | 99.53 | 72.83 |
| 8/6/2022 | 372 | 2 | 1 | 98 | 1 642 697 | 602 370 | 556 579 | 373 741 | 88.87 | 99.68 | 99.53 | 72.85 |
| 8/7/2022 | 20 | 2 | 0 | 5 | 1 642 717 | 602 372 | 556 579 | 373 746 | 88.87 | 99.68 | 99.53 | 72.85 |
| 8/8/2022 | 1097 | 6 | 19 | 209 | 1 643 814 | 602 378 | 556 598 | 373 955 | 88.93 | 99.68 | 99.53 | 72.89 |
| 8/9/2022 | 1845 | 8 | 13 | 281 | 1 645 659 | 602 386 | 556 611 | 374 236 | 89.03 | 99.68 | 99.54 | 72.95 |
| 8/10/2022 | 1877 | 8 | 14 | 303 | 1 647 536 | 602 394 | 556 625 | 374 539 | 89.13 | 99.68 | 99.54 | 73.00 |
| 8/11/2022 | 2355 | 2 | 17 | 455 | 1 649 891 | 602 396 | 556 642 | 374 994 | 89.26 | 99.68 | 99.54 | 73.09 |
| 8/12/2022 | 1231 | 9 | 11 | 166 | 1 651 122 | 602 405 | 556 653 | 375 160 | 89.32 | 99.68 | 99.54 | 73.13 |
| 8/13/2022 | 80 | 5 | 1 | 9 | 1 651 202 | 602 410 | 556 654 | 375 169 | 89.33 | 99.68 | 99.54 | 73.13 |
| 8/14/2022 | 11 | 0 | 1 | 2 | 1 651 213 | 602 410 | 556 655 | 375 171 | 89.33 | 99.68 | 99.54 | 73.13 |
| 8/15/2022 | 1020 | 2 | 17 | 185 | 1 652 233 | 602 412 | 556 672 | 375 356 | 89.38 | 99.68 | 99.55 | 73.16 |
| 8/16/2022 | 1527 | 12 | 27 | 275 | 1 653 760 | 602 424 | 556 699 | 375 631 | 89.47 | 99.68 | 99.55 | 73.22 |
| 8/17/2022 | 1468 | 8 | 32 | 233 | 1 655 228 | 602 432 | 556 731 | 375 864 | 89.54 | 99.69 | 99.56 | 73.26 |
| 8/18/2022 | 1781 | 7 | 15 | 290 | 1 657 009 | 602 439 | 556 746 | 376 154 | 89.64 | 99.69 | 99.56 | 73.32 |
| 8/19/2022 | 1253 | 2 | 16 | 222 | 1 658 262 | 602 441 | 556 762 | 376 376 | 89.71 | 99.69 | 99.56 | 73.36 |
| 8/20/2022 | 520 | 8 | 28 | 86 | 1 658 782 | 602 449 | 556 790 | 376 462 | 89.74 | 99.69 | 99.57 | 73.38 |
| 8/21/2022 | 33 | 0 | 1 | 5 | 1 658 815 | 602 449 | 556 791 | 376 467 | 89.74 | 99.69 | 99.57 | 73.38 |
| 8/22/2022 | 1189 | 4 | 33 | 160 | 1 660 004 | 602 453 | 556 824 | 376 627 | 89.80 | 99.69 | 99.57 | 73.41 |
| 8/23/2022 | 1863 | 38 | 45 | 258 | 1 661 867 | 602 491 | 556 869 | 376 885 | 89.90 | 99.70 | 99.58 | 73.46 |
| 8/24/2022 | 1334 | 17 | 14 | 200 | 1 663 201 | 602 508 | 556 883 | 377 085 | 89.98 | 99.70 | 99.58 | 73.50 |
| 8/25/2022 | 1392 | 6 | 26 | 208 | 1 664 593 | 602 514 | 556 909 | 377 293 | 90.05 | 99.70 | 99.59 | 73.54 |
| 8/26/2022 | 1182 | 6 | 18 | 161 | 1 665 775 | 602 520 | 556 927 | 377 454 | 90.12 | 99.70 | 99.59 | 73.57 |
| 8/27/2022 | 167 | 0 | 5 | 20 | 1 665 942 | 602 520 | 556 932 | 377 474 | 90.12 | 99.70 | 99.59 | 73.58 |
| 8/28/2022 | 59 | 0 | 1 | 12 | 1 666 001 | 602 520 | 556 933 | 377 486 | 90.13 | 99.70 | 99.59 | 73.58 |
| 8/29/2022 | 858 | 6 | 27 | 146 | 1 666 859 | 602 526 | 556 960 | 377 632 | 90.17 | 99.70 | 99.60 | 73.61 |
| 8/30/2022 | 1157 | 0 | 28 | 187 | 1 668 016 | 602 526 | 556 988 | 377 819 | 90.24 | 99.70 | 99.60 | 73.64 |
| 8/31/2022 | 1250 | 5 | 29 | 161 | 1 669 266 | 602 531 | 557 017 | 377 980 | 90.30 | 99.70 | 99.61 | 73.68 |
| 9/1/2022 | 1062 | 10 | 21 | 153 | 1 670 328 | 602 541 | 557 038 | 378 133 | 90.36 | 99.70 | 99.61 | 73.71 |
| 9/2/2022 | 947 | 4 | 20 | 290 | 1 671 275 | 602 545 | 557 058 | 378 423 | 90.41 | 99.70 | 99.62 | 73.76 |
| 9/3/2022 | 80 | 5 | 8 | 13 | 1 671 355 | 602 550 | 557 066 | 378 436 | 90.42 | 99.71 | 99.62 | 73.76 |
| 9/4/2022 | 10 | 0 | 0 | 3 | 1 671 365 | 602 550 | 557 066 | 378 439 | 90.42 | 99.71 | 99.62 | 73.77 |
| 9/5/2022 | 531 | 3 | 10 | 76 | 1 671 896 | 602 553 | 557 076 | 378 515 | 90.45 | 99.71 | 99.62 | 73.78 |
| 9/6/2022 | 722 | 6 | 15 | 137 | 1 672 618 | 602 559 | 557 091 | 378 652 | 90.49 | 99.71 | 99.62 | 73.81 |
| 9/7/2022 | 48 | 0 | 1 | 4 | 1 672 666 | 602 559 | 557 092 | 378 656 | 90.49 | 99.71 | 99.62 | 73.81 |
| 9/8/2022 | 851 | 0 | 7 | 100 | 1 673 517 | 602 559 | 557 099 | 378 756 | 90.53 | 99.71 | 99.62 | 73.83 |
| 9/9/2022 | 469 | 3 | 12 | 65 | 1 673 986 | 602 562 | 557 111 | 378 821 | 90.56 | 99.71 | 99.62 | 73.84 |
| 9/10/2022 | 152 | 0 | 2 | 14 | 1 674 138 | 602 562 | 557 113 | 378 835 | 90.57 | 99.71 | 99.63 | 73.84 |
| 9/11/2022 | 1 | 0 | 0 | 1 | 1 674 139 | 602 562 | 557 113 | 378 836 | 90.57 | 99.71 | 99.63 | 73.84 |
| 9/12/2022 | 407 | 1 | 15 | 78 | 1 674 546 | 602 563 | 557 128 | 378 914 | 90.59 | 99.71 | 99.63 | 73.86 |
| 9/13/2022 | 787 | 2 | 5 | 112 | 1 675 333 | 602 565 | 557 133 | 379 026 | 90.63 | 99.71 | 99.63 | 73.88 |
| 9/14/2022 | 798 | 2 | 11 | 108 | 1 676 131 | 602 567 | 557 144 | 379 134 | 90.68 | 99.71 | 99.63 | 73.90 |
| 9/15/2022 | 615 | 9 | 17 | 108 | 1 676 746 | 602 576 | 557 161 | 379 242 | 90.71 | 99.71 | 99.63 | 73.92 |
| 9/16/2022 | 617 | 13 | 11 | 71 | 1 677 363 | 602 589 | 557 172 | 379 313 | 90.74 | 99.71 | 99.64 | 73.94 |
| 9/17/2022 | 100 | 1 | 1 | 14 | 1 677 463 | 602 590 | 557 173 | 379 327 | 90.75 | 99.71 | 99.64 | 73.94 |
| 9/18/2022 | 11 | 0 | 1 | 2 | 1 677 474 | 602 590 | 557 174 | 379 329 | 90.75 | 99.71 | 99.64 | 73.94 |
| 9/19/2022 | 646 | 7 | 16 | 67 | 1 678 120 | 602 597 | 557 190 | 379 396 | 90.78 | 99.71 | 99.64 | 73.95 |
| 9/20/2022 | 743 | 1 | 13 | 91 | 1 678 863 | 602 598 | 557 203 | 379 487 | 90.82 | 99.71 | 99.64 | 73.97 |
| 9/21/2022 | 667 | 1 | 13 | 93 | 1 679 530 | 602 599 | 557 216 | 379 580 | 90.86 | 99.71 | 99.64 | 73.99 |
| 9/22/2022 | 966 | 1 | 14 | 143 | 1 680 496 | 602 600 | 557 230 | 379 723 | 90.91 | 99.71 | 99.65 | 74.02 |
| 9/23/2022 | 507 | 3 | 12 | 92 | 1 681 003 | 602 603 | 557 242 | 379 815 | 90.94 | 99.71 | 99.65 | 74.03 |
| 9/24/2022 | 64 | 1 | 6 | 15 | 1 681 067 | 602 604 | 557 248 | 379 830 | 90.94 | 99.71 | 99.65 | 74.04 |
| 9/25/2022 | 13 | 0 | 1 | 4 | 1 681 080 | 602 604 | 557 249 | 379 834 | 90.94 | 99.71 | 99.65 | 74.04 |
| 9/26/2022 | 417 | 2 | 9 | 86 | 1 681 497 | 602 606 | 557 258 | 379 920 | 90.97 | 99.71 | 99.65 | 74.05 |
| 9/27/2022 | 521 | 2 | 16 | 111 | 1 682 018 | 602 608 | 557 274 | 380 031 | 90.99 | 99.72 | 99.65 | 74.08 |
| 9/28/2022 | 607 | 4 | 11 | 103 | 1 682 625 | 602 612 | 557 285 | 380 134 | 91.03 | 99.72 | 99.66 | 74.10 |
| 9/29/2022 | 648 | 3 | 13 | 104 | 1 683 273 | 602 615 | 557 298 | 380 238 | 91.06 | 99.72 | 99.66 | 74.12 |
| 9/30/2022 | 291 | 6 | 9 | 52 | 1 683 564 | 602 621 | 557 307 | 380 290 | 91.08 | 99.72 | 99.66 | 74.13 |
| 10/1/2022 | 21 | 0 | 1 | 2 | 1 683 585 | 602 621 | 557 308 | 380 292 | 91.08 | 99.72 | 99.66 | 74.13 |
| 10/2/2022 | 2 | 0 | 0 | 0 | 1 683 587 | 602 621 | 557 308 | 380 292 | 91.08 | 99.72 | 99.66 | 74.13 |
| 10/3/2022 | 138 | 0 | 7 | 19 | 1 683 725 | 602 621 | 557 315 | 380 311 | 91.09 | 99.72 | 99.66 | 74.13 |
| 10/4/2022 | 294 | 1 | 31 | 48 | 1 684 019 | 602 622 | 557 346 | 380 359 | 91.10 | 99.72 | 99.67 | 74.14 |
| 10/5/2022 | 506 | 1 | 19 | 83 | 1 684 525 | 602 623 | 557 365 | 380 442 | 91.13 | 99.72 | 99.67 | 74.16 |
| 10/6/2022 | 437 | 17 | 12 | 51 | 1 684 962 | 602 640 | 557 377 | 380 493 | 91.15 | 99.72 | 99.67 | 74.17 |
| 10/7/2022 | 223 | 0 | 12 | 32 | 1 685 185 | 602 640 | 557 389 | 380 525 | 91.17 | 99.72 | 99.67 | 74.17 |
| 10/8/2022 | 46 | 0 | 1 | 4 | 1 685 231 | 602 640 | 557 390 | 380 529 | 91.17 | 99.72 | 99.67 | 74.17 |
| 10/9/2022 | 41 | 0 | 0 | 1 | 1 685 272 | 602 640 | 557 390 | 380 530 | 91.17 | 99.72 | 99.67 | 74.17 |
| 10/10/2022 | 319 | 0 | 6 | 52 | 1 685 591 | 602 640 | 557 396 | 380 582 | 91.19 | 99.72 | 99.68 | 74.18 |
| 10/11/2022 | 418 | 1 | 3 | 70 | 1 686 009 | 602 641 | 557 399 | 380 652 | 91.21 | 99.72 | 99.68 | 74.20 |
| 10/12/2022 | 31 | 0 | 0 | 4 | 1 686 040 | 602 641 | 557 399 | 380 656 | 91.21 | 99.72 | 99.68 | 74.20 |
| 10/13/2022 | 515 | 24 | 7 | 79 | 1 686 555 | 602 665 | 557 406 | 380 735 | 91.24 | 99.72 | 99.68 | 74.21 |
| 10/14/2022 | 228 | 0 | 3 | 41 | 1 686 783 | 602 665 | 557 409 | 380 776 | 91.25 | 99.72 | 99.68 | 74.22 |
| 10/15/2022 | 58 | 1 | 0 | 12 | 1 686 841 | 602 666 | 557 409 | 380 788 | 91.25 | 99.72 | 99.68 | 74.22 |
| 10/16/2022 | 4 | 0 | 2 | 1 | 1 686 845 | 602 666 | 557 411 | 380 789 | 91.26 | 99.72 | 99.68 | 74.22 |
| 10/17/2022 | 284 | 0 | 4 | 54 | 1 687 129 | 602 666 | 557 415 | 380 843 | 91.27 | 99.72 | 99.68 | 74.23 |
| 10/18/2022 | 428 | 0 | 13 | 58 | 1 687 557 | 602 666 | 557 428 | 380 901 | 91.29 | 99.72 | 99.68 | 74.24 |
| 10/19/2022 | 493 | 3 | 19 | 97 | 1 688 050 | 602 669 | 557 447 | 380 998 | 91.32 | 99.73 | 99.68 | 74.26 |
| 10/20/2022 | 387 | 1 | 14 | 63 | 1 688 437 | 602 670 | 557 461 | 381 061 | 91.34 | 99.73 | 99.69 | 74.28 |
| 10/21/2022 | 204 | 0 | 19 | 38 | 1 688 641 | 602 670 | 557 480 | 381 099 | 91.35 | 99.73 | 99.69 | 74.28 |
| 10/22/2022 | 71 | 0 | 1 | 11 | 1 688 712 | 602 670 | 557 481 | 381 110 | 91.36 | 99.73 | 99.69 | 74.29 |
| 10/23/2022 | 12 | 0 | 0 | 2 | 1 688 724 | 602 670 | 557 481 | 381 112 | 91.36 | 99.73 | 99.69 | 74.29 |
| 10/24/2022 | 228 | 0 | 4 | 50 | 1 688 952 | 602 670 | 557 485 | 381 162 | 91.37 | 99.73 | 99.69 | 74.30 |
| 10/25/2022 | 352 | 0 | 6 | 66 | 1 689 304 | 602 670 | 557 491 | 381 228 | 91.39 | 99.73 | 99.69 | 74.31 |
| 10/26/2022 | 577 | 1 | 12 | 109 | 1 689 881 | 602 671 | 557 503 | 381 337 | 91.42 | 99.73 | 99.69 | 74.33 |
| 10/27/2022 | 356 | 4 | 6 | 73 | 1 690 237 | 602 675 | 557 509 | 381 410 | 91.44 | 99.73 | 99.70 | 74.34 |
| 10/28/2022 | 148 | 1 | 1 | 25 | 1 690 385 | 602 676 | 557 510 | 381 435 | 91.45 | 99.73 | 99.70 | 74.35 |
| 10/29/2022 | 6 | 0 | 1 | 1 | 1 690 391 | 602 676 | 557 511 | 381 436 | 91.45 | 99.73 | 99.70 | 74.35 |
| 10/30/2022 | 1 | 0 | 0 | 0 | 1 690 392 | 602 676 | 557 511 | 381 436 | 91.45 | 99.73 | 99.70 | 74.35 |
| 10/31/2022 | 117 | 0 | 8 | 22 | 1 690 509 | 602 676 | 557 519 | 381 458 | 91.45 | 99.73 | 99.70 | 74.35 |
| 11/1/2022 | 191 | 0 | 5 | 30 | 1 690 700 | 602 676 | 557 524 | 381 488 | 91.46 | 99.73 | 99.70 | 74.36 |
| 11/2/2022 | 12 | 0 | 0 | 4 | 1 690 712 | 602 676 | 557 524 | 381 492 | 91.46 | 99.73 | 99.70 | 74.36 |
| 11/3/2022 | 218 | 1 | 7 | 44 | 1 690 930 | 602 677 | 557 531 | 381 536 | 91.48 | 99.73 | 99.70 | 74.37 |
| 11/4/2022 | 164 | 0 | 4 | 37 | 1 691 094 | 602 677 | 557 535 | 381 573 | 91.48 | 99.73 | 99.70 | 74.38 |
| 11/5/2022 | 21 | 0 | 0 | 4 | 1 691 115 | 602 677 | 557 535 | 381 577 | 91.49 | 99.73 | 99.70 | 74.38 |
| 11/6/2022 | 7 | 0 | 0 | 3 | 1 691 122 | 602 677 | 557 535 | 381 580 | 91.49 | 99.73 | 99.70 | 74.38 |
| 11/7/2022 | 151 | 1 | 4 | 36 | 1 691 273 | 602 678 | 557 539 | 381 616 | 91.49 | 99.73 | 99.70 | 74.38 |
| 11/8/2022 | 219 | 0 | 3 | 34 | 1 691 492 | 602 678 | 557 542 | 381 650 | 91.51 | 99.73 | 99.70 | 74.39 |
| 11/9/2022 | 299 | 1 | 5 | 57 | 1 691 791 | 602 679 | 557 547 | 381 707 | 91.52 | 99.73 | 99.70 | 74.40 |
| 11/10/2022 | 361 | 4 | 11 | 68 | 1 692 152 | 602 683 | 557 558 | 381 775 | 91.54 | 99.73 | 99.70 | 74.42 |
| 11/11/2022 | 283 | 0 | 4 | 39 | 1 692 435 | 602 683 | 557 562 | 381 814 | 91.56 | 99.73 | 99.71 | 74.42 |
| 11/12/2022 | 19 | 0 | 0 | 1 | 1 692 454 | 602 683 | 557 562 | 381 815 | 91.56 | 99.73 | 99.71 | 74.42 |
| 11/13/2022 | 2 | 0 | 0 | 0 | 1 692 456 | 602 683 | 557 562 | 381 815 | 91.56 | 99.73 | 99.71 | 74.42 |
| 11/14/2022 | 103 | 0 | 1 | 21 | 1 692 559 | 602 683 | 557 563 | 381 836 | 91.56 | 99.73 | 99.71 | 74.43 |
| 11/15/2022 | 21 | 1 | 0 | 7 | 1 692 580 | 602 684 | 557 563 | 381 843 | 91.57 | 99.73 | 99.71 | 74.43 |
| 11/16/2022 | 519 | 2 | 15 | 101 | 1 693 099 | 602 686 | 557 578 | 381 944 | 91.59 | 99.73 | 99.71 | 74.45 |
| 11/17/2022 | 730 | 0 | 31 | 130 | 1 693 829 | 602 686 | 557 609 | 382 074 | 91.63 | 99.73 | 99.71 | 74.47 |
| 11/18/2022 | 514 | 7 | 19 | 105 | 1 694 343 | 602 693 | 557 628 | 382 179 | 91.66 | 99.73 | 99.72 | 74.49 |
| 11/19/2022 | 97 | 0 | 1 | 21 | 1 694 440 | 602 693 | 557 629 | 382 200 | 91.67 | 99.73 | 99.72 | 74.50 |
| 11/20/2022 | 16 | 0 | 0 | 4 | 1 694 456 | 602 693 | 557 629 | 382 204 | 91.67 | 99.73 | 99.72 | 74.50 |
| 11/21/2022 | 597 | 2 | 9 | 108 | 1 695 053 | 602 695 | 557 638 | 382 312 | 91.70 | 99.73 | 99.72 | 74.52 |
| 11/22/2022 | 828 | 1 | 13 | 140 | 1 695 881 | 602 696 | 557 651 | 382 452 | 91.74 | 99.73 | 99.72 | 74.55 |
| 11/23/2022 | 838 | 3 | 15 | 154 | 1 696 719 | 602 699 | 557 666 | 382 606 | 91.79 | 99.73 | 99.72 | 74.58 |
| 11/24/2022 | 736 | 0 | 21 | 124 | 1 697 455 | 602 699 | 557 687 | 382 730 | 91.83 | 99.73 | 99.73 | 74.60 |
| 11/25/2022 | 484 | 6 | 10 | 80 | 1 697 939 | 602 705 | 557 697 | 382 810 | 91.86 | 99.73 | 99.73 | 74.62 |
| 11/26/2022 | 247 | 11 | 3 | 56 | 1 698 186 | 602 716 | 557 700 | 382 866 | 91.87 | 99.73 | 99.73 | 74.63 |
| 11/27/2022 | 5 | 0 | 0 | 1 | 1 698 191 | 602 716 | 557 700 | 382 867 | 91.87 | 99.73 | 99.73 | 74.63 |
| 11/28/2022 | 399 | 2 | 9 | 64 | 1 698 590 | 602 718 | 557 709 | 382 931 | 91.89 | 99.73 | 99.73 | 74.64 |
| 11/29/2022 | 737 | 6 | 13 | 145 | 1 699 327 | 602 724 | 557 722 | 383 076 | 91.93 | 99.73 | 99.73 | 74.67 |
| 11/30/2022 | 926 | 3 | 4 | 144 | 1 700 253 | 602 727 | 557 726 | 383 220 | 91.98 | 99.73 | 99.73 | 74.70 |
| 12/1/2022 | 722 | 2 | 16 | 114 | 1 700 975 | 602 729 | 557 742 | 383 334 | 92.02 | 99.74 | 99.74 | 74.72 |
| 12/2/2022 | 294 | 3 | 10 | 54 | 1 701 269 | 602 732 | 557 752 | 383 388 | 92.04 | 99.74 | 99.74 | 74.73 |
| 12/3/2022 | 158 | 1 | 2 | 28 | 1 701 427 | 602 733 | 557 754 | 383 416 | 92.04 | 99.74 | 99.74 | 74.74 |
| 12/4/2022 | 4 | 0 | 0 | 1 | 1 701 431 | 602 733 | 557 754 | 383 417 | 92.04 | 99.74 | 99.74 | 74.74 |
| 12/5/2022 | 454 | 12 | 11 | 79 | 1 701 885 | 602 745 | 557 765 | 383 496 | 92.07 | 99.74 | 99.74 | 74.75 |
| 12/6/2022 | 614 | 1 | 7 | 118 | 1 702 499 | 602 746 | 557 772 | 383 614 | 92.10 | 99.74 | 99.74 | 74.77 |
| 12/7/2022 | 847 | 6 | 22 | 120 | 1 703 346 | 602 752 | 557 794 | 383 734 | 92.15 | 99.74 | 99.75 | 74.80 |
| 12/8/2022 | 452 | 1 | 4 | 64 | 1 703 798 | 602 753 | 557 798 | 383 798 | 92.17 | 99.74 | 99.75 | 74.81 |
| 12/9/2022 | 165 | 1 | 1 | 46 | 1 703 963 | 602 754 | 557 799 | 383 844 | 92.18 | 99.74 | 99.75 | 74.82 |
| 12/10/2022 | 24 | 0 | 1 | 6 | 1 703 987 | 602 754 | 557 800 | 383 850 | 92.18 | 99.74 | 99.75 | 74.82 |
| 12/11/2022 | 4 | 0 | 0 | 1 | 1 703 991 | 602 754 | 557 800 | 383 851 | 92.18 | 99.74 | 99.75 | 74.82 |
| 12/12/2022 | 535 | 7 | 13 | 89 | 1 704 526 | 602 761 | 557 813 | 383 940 | 92.21 | 99.74 | 99.75 | 74.84 |
| 12/13/2022 | 706 | 4 | 13 | 145 | 1 705 232 | 602 765 | 557 826 | 384 085 | 92.25 | 99.74 | 99.75 | 74.87 |
| 12/14/2022 | 799 | 5 | 20 | 138 | 1 706 031 | 602 770 | 557 846 | 384 223 | 92.29 | 99.74 | 99.76 | 74.89 |
| 12/15/2022 | 588 | 22 | 9 | 119 | 1 706 619 | 602 792 | 557 855 | 384 342 | 92.32 | 99.75 | 99.76 | 74.92 |
| 12/16/2022 | 370 | 3 | 7 | 64 | 1 706 989 | 602 795 | 557 862 | 384 406 | 92.34 | 99.75 | 99.76 | 74.93 |
| 12/17/2022 | 55 | 1 | 2 | 14 | 1 707 044 | 602 796 | 557 864 | 384 420 | 92.35 | 99.75 | 99.76 | 74.93 |
| 12/18/2022 | 6 | 0 | 0 | 3 | 1 707 050 | 602 796 | 557 864 | 384 423 | 92.35 | 99.75 | 99.76 | 74.93 |
| 12/19/2022 | 446 | 4 | 4 | 75 | 1 707 496 | 602 800 | 557 868 | 384 498 | 92.37 | 99.75 | 99.76 | 74.95 |
| 12/20/2022 | 479 | 5 | 4 | 103 | 1 707 975 | 602 805 | 557 872 | 384 601 | 92.40 | 99.75 | 99.76 | 74.97 |
| 12/21/2022 | 411 | 14 | 15 | 68 | 1 708 386 | 602 819 | 557 887 | 384 669 | 92.42 | 99.75 | 99.76 | 74.98 |
| 12/22/2022 | 366 | 1 | 1 | 75 | 1 708 752 | 602 820 | 557 888 | 384 744 | 92.44 | 99.75 | 99.76 | 74.99 |
| 12/23/2022 | 124 | 0 | 1 | 18 | 1 708 876 | 602 820 | 557 889 | 384 762 | 92.45 | 99.75 | 99.76 | 75.00 |
| 12/24/2022 | 3 | 0 | 0 | 1 | 1 708 879 | 602 820 | 557 889 | 384 763 | 92.45 | 99.75 | 99.76 | 75.00 |
| 12/25/2022 | 2 | 0 | 0 | 2 | 1 708 881 | 602 820 | 557 889 | 384 765 | 92.45 | 99.75 | 99.76 | 75.00 |
| 12/26/2022 | 119 | 0 | 2 | 30 | 1 709 000 | 602 820 | 557 891 | 384 795 | 92.45 | 99.75 | 99.76 | 75.00 |
| 12/27/2022 | 190 | 0 | 6 | 42 | 1 709 190 | 602 820 | 557 897 | 384 837 | 92.46 | 99.75 | 99.77 | 75.01 |
| 12/28/2022 | 263 | 5 | 7 | 38 | 1 709 453 | 602 825 | 557 904 | 384 875 | 92.48 | 99.75 | 99.77 | 75.02 |
| 12/29/2022 | 177 | 1 | 4 | 33 | 1 709 630 | 602 826 | 557 908 | 384 908 | 92.49 | 99.75 | 99.77 | 75.03 |
| 12/30/2022 | 43 | 0 | 1 | 11 | 1 709 673 | 602 826 | 557 909 | 384 919 | 92.49 | 99.75 | 99.77 | 75.03 |
| 12/31/2022 | 2 | 0 | 0 | 0 | 1 709 675 | 602 826 | 557 909 | 384 919 | 92.49 | 99.75 | 99.77 | 75.03 |
| 1/1/2023 | 21 | 0 | 0 | 1 | 1 709 696 | 602 826 | 557 909 | 384 920 | 92.49 | 99.75 | 99.77 | 75.03 |
| 1/2/2023 | 87 | 10 | 5 | 8 | 1 709 783 | 602 836 | 557 914 | 384 928 | 92.50 | 99.75 | 99.77 | 75.03 |
| 1/3/2023 | 192 | 12 | 5 | 47 | 1 709 975 | 602 848 | 557 919 | 384 975 | 92.51 | 99.75 | 99.77 | 75.04 |
| 1/4/2023 | 260 | 9 | 4 | 51 | 1 710 235 | 602 857 | 557 923 | 385 026 | 92.52 | 99.76 | 99.77 | 75.05 |
| 1/5/2023 | 251 | 0 | 3 | 58 | 1 710 486 | 602 857 | 557 926 | 385 084 | 92.53 | 99.76 | 99.77 | 75.06 |
| 1/6/2023 | 214 | 1 | 5 | 50 | 1 710 700 | 602 858 | 557 931 | 385 134 | 92.55 | 99.76 | 99.77 | 75.07 |
| 1/7/2023 | 9 | 0 | 0 | 2 | 1 710 709 | 602 858 | 557 931 | 385 136 | 92.55 | 99.76 | 99.77 | 75.07 |
| 1/8/2023 | 2 | 0 | 0 | 1 | 1 710 711 | 602 858 | 557 931 | 385 137 | 92.55 | 99.76 | 99.77 | 75.07 |
| 1/9/2023 | 272 | 16 | 7 | 62 | 1 710 983 | 602 874 | 557 938 | 385 199 | 92.56 | 99.76 | 99.77 | 75.08 |
| 1/10/2023 | 325 | 1 | 4 | 61 | 1 711 308 | 602 875 | 557 942 | 385 260 | 92.58 | 99.76 | 99.77 | 75.09 |
| 1/11/2023 | 278 | 1 | 6 | 66 | 1 711 586 | 602 876 | 557 948 | 385 326 | 92.59 | 99.76 | 99.77 | 75.11 |
| 1/12/2023 | 421 | 9 | 14 | 91 | 1 712 007 | 602 885 | 557 962 | 385 417 | 92.62 | 99.76 | 99.78 | 75.13 |
| 1/13/2023 | 337 | 10 | 5 | 52 | 1 712 344 | 602 895 | 557 967 | 385 469 | 92.63 | 99.76 | 99.78 | 75.14 |
| 1/14/2023 | 20 | 0 | 1 | 2 | 1 712 364 | 602 895 | 557 968 | 385 471 | 92.64 | 99.76 | 99.78 | 75.14 |
| 1/15/2023 | 15 | 0 | 0 | 6 | 1 712 379 | 602 895 | 557 968 | 385 477 | 92.64 | 99.76 | 99.78 | 75.14 |
| 1/16/2023 | 229 | 4 | 12 | 53 | 1 712 608 | 602 899 | 557 980 | 385 530 | 92.65 | 99.76 | 99.78 | 75.15 |
| 1/17/2023 | 276 | 16 | 6 | 68 | 1 712 884 | 602 915 | 557 986 | 385 598 | 92.66 | 99.77 | 99.78 | 75.16 |
| 1/18/2023 | 464 | 2 | 6 | 159 | 1 713 348 | 602 917 | 557 992 | 385 757 | 92.69 | 99.77 | 99.78 | 75.19 |
| 1/19/2023 | 310 | 1 | 15 | 68 | 1 713 658 | 602 918 | 558 007 | 385 825 | 92.71 | 99.77 | 99.79 | 75.20 |
| 1/20/2023 | 214 | 0 | 12 | 57 | 1 713 872 | 602 918 | 558 019 | 385 882 | 92.72 | 99.77 | 99.79 | 75.22 |
| 1/21/2023 | 23 | 0 | 1 | 1 | 1 713 895 | 602 918 | 558 020 | 385 883 | 92.72 | 99.77 | 99.79 | 75.22 |
| 1/22/2023 | 7 | 0 | 0 | 2 | 1 713 902 | 602 918 | 558 020 | 385 885 | 92.72 | 99.77 | 99.79 | 75.22 |
| 1/23/2023 | 216 | 3 | 8 | 40 | 1 714 118 | 602 921 | 558 028 | 385 925 | 92.73 | 99.77 | 99.79 | 75.22 |
| 1/24/2023 | 332 | 12 | 16 | 76 | 1 714 450 | 602 933 | 558 044 | 386 001 | 92.75 | 99.77 | 99.79 | 75.24 |
| 1/25/2023 | 309 | 2 | 7 | 76 | 1 714 759 | 602 935 | 558 051 | 386 077 | 92.77 | 99.77 | 99.79 | 75.25 |
| 1/26/2023 | 325 | 6 | 11 | 75 | 1 715 084 | 602 941 | 558 062 | 386 152 | 92.78 | 99.77 | 99.79 | 75.27 |
| 1/27/2023 | 223 | 3 | 4 | 37 | 1 715 307 | 602 944 | 558 066 | 386 189 | 92.79 | 99.77 | 99.80 | 75.28 |
| 1/28/2023 | 15 | 0 | 0 | 11 | 1 715 322 | 602 944 | 558 066 | 386 200 | 92.80 | 99.77 | 99.80 | 75.28 |
| 1/29/2023 | 3 | 0 | 0 | 0 | 1 715 325 | 602 944 | 558 066 | 386 200 | 92.80 | 99.77 | 99.80 | 75.28 |
| 1/30/2023 | 225 | 1 | 14 | 42 | 1 715 550 | 602 945 | 558 080 | 386 242 | 92.81 | 99.77 | 99.80 | 75.29 |
| 1/31/2023 | 239 | 1 | 7 | 53 | 1 715 789 | 602 946 | 558 087 | 386 295 | 92.82 | 99.77 | 99.80 | 75.30 |
| 2/1/2023 | 243 | 3 | 2 | 61 | 1 716 032 | 602 949 | 558 089 | 386 356 | 92.83 | 99.77 | 99.80 | 75.31 |
| 2/2/2023 | 243 | 10 | 9 | 46 | 1 716 275 | 602 959 | 558 098 | 386 402 | 92.85 | 99.77 | 99.80 | 75.32 |
| 2/3/2023 | 180 | 1 | 6 | 37 | 1 716 455 | 602 960 | 558 104 | 386 439 | 92.86 | 99.77 | 99.80 | 75.32 |
| 2/4/2023 | 83 | 10 | 5 | 30 | 1 716 538 | 602 970 | 558 109 | 386 469 | 92.86 | 99.78 | 99.80 | 75.33 |
| 2/5/2023 | 11 | 0 | 0 | 4 | 1 716 549 | 602 970 | 558 109 | 386 473 | 92.86 | 99.78 | 99.80 | 75.33 |
| 2/6/2023 | 171 | 8 | 13 | 40 | 1 716 720 | 602 978 | 558 122 | 386 513 | 92.87 | 99.78 | 99.81 | 75.34 |
| 2/7/2023 | 343 | 27 | 5 | 67 | 1 717 063 | 603 005 | 558 127 | 386 580 | 92.89 | 99.78 | 99.81 | 75.35 |
| 2/8/2023 | 315 | 1 | 7 | 91 | 1 717 378 | 603 006 | 558 134 | 386 671 | 92.91 | 99.78 | 99.81 | 75.37 |
| 2/9/2023 | 462 | 20 | 22 | 137 | 1 717 840 | 603 026 | 558 156 | 386 808 | 92.93 | 99.78 | 99.81 | 75.40 |
| 2/10/2023 | 179 | 0 | 6 | 46 | 1 718 019 | 603 026 | 558 162 | 386 854 | 92.94 | 99.78 | 99.81 | 75.41 |
| 2/11/2023 | 45 | 1 | 4 | 21 | 1 718 064 | 603 027 | 558 166 | 386 875 | 92.94 | 99.78 | 99.81 | 75.41 |
| 2/12/2023 | 2 | 0 | 0 | 0 | 1 718 066 | 603 027 | 558 166 | 386 875 | 92.94 | 99.78 | 99.81 | 75.41 |
| 2/13/2023 | 257 | 36 | 13 | 76 | 1 718 323 | 603 063 | 558 179 | 386 951 | 92.96 | 99.79 | 99.82 | 75.42 |
| 2/14/2023 | 272 | 4 | 15 | 83 | 1 718 595 | 603 067 | 558 194 | 387 034 | 92.97 | 99.79 | 99.82 | 75.44 |
| 2/15/2023 | 321 | 7 | 13 | 84 | 1 718 916 | 603 074 | 558 207 | 387 118 | 92.99 | 99.79 | 99.82 | 75.46 |
| 2/16/2023 | 267 | 9 | 14 | 65 | 1 719 183 | 603 083 | 558 221 | 387 183 | 93.00 | 99.79 | 99.82 | 75.47 |
| 2/17/2023 | 181 | 8 | 2 | 39 | 1 719 364 | 603 091 | 558 223 | 387 222 | 93.01 | 99.80 | 99.82 | 75.48 |
| 2/18/2023 | 2 | 1 | 0 | 0 | 1 719 366 | 603 092 | 558 223 | 387 222 | 93.01 | 99.80 | 99.82 | 75.48 |
| 2/20/2023 | 3 | 1 | 0 | 0 | 1 719 369 | 603 093 | 558 223 | 387 222 | 93.01 | 99.80 | 99.82 | 75.48 |
| 2/21/2023 | 4 | 0 | 0 | 3 | 1 719 373 | 603 093 | 558 223 | 387 225 | 93.01 | 99.80 | 99.82 | 75.48 |
| 2/22/2023 | 40 | 0 | 0 | 31 | 1 719 413 | 603 093 | 558 223 | 387 256 | 93.02 | 99.80 | 99.82 | 75.48 |
| 2/23/2023 | 236 | 11 | 6 | 86 | 1 719 649 | 603 104 | 558 229 | 387 342 | 93.03 | 99.80 | 99.82 | 75.50 |
| 2/24/2023 | 182 | 21 | 5 | 51 | 1 719 831 | 603 125 | 558 234 | 387 393 | 93.04 | 99.80 | 99.83 | 75.51 |
| 2/25/2023 | 40 | 0 | 0 | 30 | 1 719 871 | 603 125 | 558 234 | 387 423 | 93.04 | 99.80 | 99.83 | 75.52 |
| 2/27/2023 | 811 | 12 | 12 | 732 | 1 720 682 | 603 137 | 558 246 | 388 155 | 93.09 | 99.80 | 99.83 | 75.66 |
| 2/28/2023 | 1890 | 48 | 46 | 1727 | 1 722 572 | 603 185 | 558 292 | 389 882 | 93.19 | 99.81 | 99.84 | 76.00 |
| 3/1/2023 | 2905 | 4 | 11 | 2823 | 1 725 477 | 603 189 | 558 303 | 392 705 | 93.35 | 99.81 | 99.84 | 76.55 |
| 3/2/2023 | 4137 | 29 | 7 | 4045 | 1 729 614 | 603 218 | 558 310 | 396 750 | 93.57 | 99.82 | 99.84 | 77.33 |
| 3/3/2023 | 2892 | 17 | 8 | 2838 | 1 732 506 | 603 235 | 558 318 | 399 588 | 93.73 | 99.82 | 99.84 | 77.89 |
| 3/4/2023 | 407 | 5 | 2 | 395 | 1 732 913 | 603 240 | 558 320 | 399 983 | 93.75 | 99.82 | 99.84 | 77.96 |
| 3/5/2023 | 6 | 0 | 0 | 4 | 1 732 919 | 603 240 | 558 320 | 399 987 | 93.75 | 99.82 | 99.84 | 77.97 |
| 3/6/2023 | 2624 | 9 | 7 | 2594 | 1 735 543 | 603 249 | 558 327 | 402 581 | 93.89 | 99.82 | 99.84 | 78.47 |
| 3/7/2023 | 4641 | 44 | 13 | 4550 | 1 740 184 | 603 293 | 558 340 | 407 131 | 94.14 | 99.83 | 99.84 | 79.36 |
| 3/8/2023 | 5035 | 6 | 7 | 4993 | 1 745 219 | 603 299 | 558 347 | 412 124 | 94.41 | 99.83 | 99.85 | 80.33 |
| 3/9/2023 | 4962 | 14 | 9 | 4924 | 1 750 181 | 603 313 | 558 356 | 417 048 | 94.68 | 99.83 | 99.85 | 81.29 |
| 3/10/2023 | 3509 | 35 | 9 | 3437 | 1 753 690 | 603 348 | 558 365 | 420 485 | 94.87 | 99.84 | 99.85 | 81.96 |
| 3/11/2023 | 777 | 14 | 6 | 741 | 1 754 467 | 603 362 | 558 371 | 421 226 | 94.91 | 99.84 | 99.85 | 82.11 |
| 3/12/2023 | 52 | 0 | 0 | 51 | 1 754 519 | 603 362 | 558 371 | 421 277 | 94.92 | 99.84 | 99.85 | 82.11 |
| 3/13/2023 | 3688 | 7 | 27 | 3641 | 1 758 207 | 603 369 | 558 398 | 424 918 | 95.12 | 99.84 | 99.85 | 82.82 |
| 3/14/2023 | 4826 | 22 | 25 | 4757 | 1 763 033 | 603 391 | 558 423 | 429 675 | 95.38 | 99.84 | 99.86 | 83.75 |
| 3/15/2023 | 4544 | 28 | 23 | 4468 | 1 767 577 | 603 419 | 558 446 | 434 143 | 95.62 | 99.85 | 99.86 | 84.62 |
| 3/16/2023 | 4601 | 8 | 5 | 4560 | 1 772 178 | 603 427 | 558 451 | 438 703 | 95.87 | 99.85 | 99.86 | 85.51 |
| 3/17/2023 | 2579 | 21 | 12 | 2536 | 1 774 757 | 603 448 | 558 463 | 441 239 | 96.01 | 99.85 | 99.87 | 86.01 |
| 3/18/2023 | 1726 | 30 | 67 | 1626 | 1 776 483 | 603 478 | 558 530 | 442 865 | 96.10 | 99.86 | 99.88 | 86.32 |
| 3/19/2023 | 3 | 0 | 0 | 3 | 1 776 486 | 603 478 | 558 530 | 442 868 | 96.10 | 99.86 | 99.88 | 86.32 |
| 3/20/2023 | 2876 | 14 | 7 | 2842 | 1 779 362 | 603 492 | 558 537 | 445 710 | 96.26 | 99.86 | 99.88 | 86.88 |
| 3/21/2023 | 3823 | 21 | 5 | 3771 | 1 783 185 | 603 513 | 558 542 | 449 481 | 96.47 | 99.86 | 99.88 | 87.61 |
| 3/22/2023 | 3979 | 51 | 21 | 3861 | 1 787 164 | 603 564 | 558 563 | 453 342 | 96.68 | 99.87 | 99.88 | 88.37 |
| 3/23/2023 | 5211 | 34 | 23 | 5132 | 1 792 375 | 603 598 | 558 586 | 458 474 | 96.96 | 99.88 | 99.89 | 89.37 |
| 3/24/2023 | 2561 | 57 | 31 | 2461 | 1 794 936 | 603 655 | 558 617 | 460 935 | 97.10 | 99.89 | 99.89 | 89.85 |
| 3/25/2023 | 587 | 2 | 7 | 578 | 1 795 523 | 603 657 | 558 624 | 461 513 | 97.13 | 99.89 | 99.90 | 89.96 |
| 3/26/2023 | 6 | 1 | 0 | 5 | 1 795 529 | 603 658 | 558 624 | 461 518 | 97.13 | 99.89 | 99.90 | 89.96 |
| 3/27/2023 | 2024 | 29 | 11 | 1967 | 1 797 553 | 603 687 | 558 635 | 463 485 | 97.24 | 99.89 | 99.90 | 90.34 |
| 3/28/2023 | 3438 | 68 | 36 | 3311 | 1 800 991 | 603 755 | 558 671 | 466 796 | 97.43 | 99.91 | 99.90 | 90.99 |
| 3/29/2023 | 4019 | 67 | 39 | 3899 | 1 805 010 | 603 822 | 558 710 | 470 695 | 97.65 | 99.92 | 99.91 | 91.75 |
| 3/30/2023 | 3423 | 9 | 18 | 3388 | 1 808 433 | 603 831 | 558 728 | 474 083 | 97.83 | 99.92 | 99.91 | 92.41 |
| 3/31/2023 | 1982 | 75 | 33 | 1865 | 1 810 415 | 603 906 | 558 761 | 475 948 | 97.94 | 99.93 | 99.92 | 92.77 |
| 4/1/2023 | 264 | 0 | 0 | 263 | 1 810 679 | 603 906 | 558 761 | 476 211 | 97.95 | 99.93 | 99.92 | 92.82 |
| 4/2/2023 | 48 | 0 | 0 | 48 | 1 810 727 | 603 906 | 558 761 | 476 259 | 97.96 | 99.93 | 99.92 | 92.83 |
| 4/3/2023 | 1536 | 15 | 14 | 1503 | 1 812 263 | 603 921 | 558 775 | 477 762 | 98.04 | 99.93 | 99.92 | 93.13 |
| 4/4/2023 | 2114 | 49 | 64 | 1993 | 1 814 377 | 603 970 | 558 839 | 479 755 | 98.15 | 99.94 | 99.93 | 93.51 |
| 4/5/2023 | 1258 | 12 | 3 | 1238 | 1 815 635 | 603 982 | 558 842 | 480 993 | 98.22 | 99.94 | 99.93 | 93.75 |
| 4/6/2023 | 250 | 0 | 0 | 248 | 1 815 885 | 603 982 | 558 842 | 481 241 | 98.24 | 99.94 | 99.93 | 93.80 |
| 4/7/2023 | 71 | 0 | 0 | 70 | 1 815 956 | 603 982 | 558 842 | 481 311 | 98.24 | 99.94 | 99.93 | 93.82 |
| 4/8/2023 | 2 | 0 | 0 | 2 | 1 815 958 | 603 982 | 558 842 | 481 313 | 98.24 | 99.94 | 99.93 | 93.82 |
| 4/10/2023 | 1149 | 11 | 4 | 1131 | 1 817 107 | 603 993 | 558 846 | 482 444 | 98.30 | 99.94 | 99.94 | 94.04 |
| 4/11/2023 | 1841 | 14 | 1 | 1823 | 1 818 948 | 604 007 | 558 847 | 484 267 | 98.40 | 99.95 | 99.94 | 94.39 |
| 4/12/2023 | 2802 | 20 | 13 | 2759 | 1 821 750 | 604 027 | 558 860 | 487 026 | 98.55 | 99.95 | 99.94 | 94.93 |
| 4/13/2023 | 1962 | 21 | 11 | 1921 | 1 823 712 | 604 048 | 558 871 | 488 947 | 98.66 | 99.95 | 99.94 | 95.31 |
| 4/14/2023 | 1539 | 18 | 8 | 1509 | 1 825 251 | 604 066 | 558 879 | 490 456 | 98.74 | 99.96 | 99.94 | 95.60 |
| 4/15/2023 | 713 | 7 | 9 | 692 | 1 825 964 | 604 073 | 558 888 | 491 148 | 98.78 | 99.96 | 99.94 | 95.73 |
| 4/16/2023 | 11 | 0 | 0 | 11 | 1 825 975 | 604 073 | 558 888 | 491 159 | 98.78 | 99.96 | 99.94 | 95.74 |
| 4/17/2023 | 940 | 16 | 14 | 902 | 1 826 915 | 604 089 | 558 902 | 492 061 | 98.83 | 99.96 | 99.95 | 95.91 |
| 4/18/2023 | 1745 | 17 | 30 | 1687 | 1 828 660 | 604 106 | 558 932 | 493 748 | 98.93 | 99.96 | 99.95 | 96.24 |
| 4/19/2023 | 1939 | 25 | 30 | 1883 | 1 830 599 | 604 131 | 558 962 | 495 631 | 99.03 | 99.97 | 99.96 | 96.61 |
| 4/20/2023 | 1502 | 22 | 17 | 1457 | 1 832 101 | 604 153 | 558 979 | 497 088 | 99.11 | 99.97 | 99.96 | 96.89 |
| 4/21/2023 | 34 | 0 | 2 | 32 | 1 832 135 | 604 153 | 558 981 | 497 120 | 99.12 | 99.97 | 99.96 | 96.90 |
| 4/22/2023 | 147 | 0 | 0 | 147 | 1 832 282 | 604 153 | 558 981 | 497 267 | 99.12 | 99.97 | 99.96 | 96.93 |
| 4/23/2023 | 62 | 1 | 1 | 60 | 1 832 344 | 604 154 | 558 982 | 497 327 | 99.13 | 99.97 | 99.96 | 96.94 |
| 4/24/2023 | 859 | 9 | 13 | 831 | 1 833 203 | 604 163 | 558 995 | 498 158 | 99.17 | 99.97 | 99.96 | 97.10 |
| 4/25/2023 | 1244 | 9 | 29 | 1203 | 1 834 447 | 604 172 | 559 024 | 499 361 | 99.24 | 99.97 | 99.97 | 97.34 |
| 4/26/2023 | 1120 | 20 | 23 | 1070 | 1 835 567 | 604 192 | 559 047 | 500 431 | 99.30 | 99.98 | 99.97 | 97.54 |
| 4/27/2023 | 1523 | 14 | 8 | 1500 | 1 837 090 | 604 206 | 559 055 | 501 931 | 99.38 | 99.98 | 99.97 | 97.84 |
| 4/28/2023 | 729 | 14 | 10 | 703 | 1 837 819 | 604 220 | 559 065 | 502 634 | 99.42 | 99.98 | 99.97 | 97.97 |
| 4/29/2023 | 195 | 5 | 6 | 184 | 1 838 014 | 604 225 | 559 071 | 502 818 | 99.43 | 99.98 | 99.98 | 98.01 |
| 4/30/2023 | 19 | 0 | 0 | 19 | 1 838 033 | 604 225 | 559 071 | 502 837 | 99.43 | 99.98 | 99.98 | 98.01 |
| 5/1/2023 | 15 | 0 | 0 | 15 | 1 838 048 | 604 225 | 559 071 | 502 852 | 99.43 | 99.98 | 99.98 | 98.02 |
| 5/2/2023 | 739 | 4 | 11 | 724 | 1 838 787 | 604 229 | 559 082 | 503 576 | 99.47 | 99.98 | 99.98 | 98.16 |
| 5/3/2023 | 891 | 35 | 26 | 824 | 1 839 678 | 604 264 | 559 108 | 504 400 | 99.52 | 99.99 | 99.98 | 98.32 |
| 5/4/2023 | 1120 | 7 | 9 | 1099 | 1 840 798 | 604 271 | 559 117 | 505 499 | 99.58 | 99.99 | 99.98 | 98.53 |
| 5/5/2023 | 722 | 5 | 18 | 699 | 1 841 520 | 604 276 | 559 135 | 506 198 | 99.62 | 99.99 | 99.99 | 98.67 |
| 5/6/2023 | 1331 | 7 | 5 | 1318 | 1 842 851 | 604 283 | 559 140 | 507 516 | 99.69 | 99.99 | 99.99 | 98.92 |
| 5/7/2023 | 4 | 0 | 0 | 4 | 1 842 855 | 604 283 | 559 140 | 507 520 | 99.69 | 99.99 | 99.99 | 98.93 |
| 5/8/2023 | 796 | 12 | 23 | 759 | 1 843 651 | 604 295 | 559 163 | 508 279 | 99.74 | 99.99 | 99.99 | 99.07 |
| 5/9/2023 | 891 | 11 | 4 | 876 | 1 844 542 | 604 306 | 559 167 | 509 155 | 99.79 | 100 | 99.99 | 99.24 |
| 5/10/2023 | 916 | 1 | 2 | 909 | 1 845 458 | 604 307 | 559 169 | 510 064 | 99.84 | 100 | 99.99 | 99.42 |
| 5/11/2023 | 612 | 6 | 12 | 594 | 1 846 070 | 604 313 | 559 181 | 510 658 | 99.87 | 100 | 99.99 | 99.54 |
| 5/12/2023 | 364 | 0 | 3 | 360 | 1 846 434 | 604 313 | 559 184 | 511 018 | 99.89 | 100 | 100 | 99.61 |
| 5/13/2023 | 20 | 0 | 0 | 20 | 1 846 454 | 604 313 | 559 184 | 511 038 | 99.89 | 100 | 100 | 99.61 |
| 5/15/2023 | 507 | 2 | 8 | 495 | 1 846 961 | 604 315 | 559 192 | 511 533 | 99.92 | 100 | 100 | 99.71 |
| 5/16/2023 | 505 | 1 | 1 | 503 | 1 847 466 | 604 316 | 559 193 | 512 036 | 99.94 | 100 | 100 | 99.81 |
| 5/17/2023 | 494 | 8 | 1 | 484 | 1 847 960 | 604 324 | 559 194 | 512 520 | 99.97 | 100 | 100 | 99.90 |
| 5/18/2023 | 314 | 3 | 15 | 295 | 1 848 274 | 604 327 | 559 209 | 512 815 | 99.99 | 100 | 100 | 99.96 |
| 5/19/2023 | 169 | 2 | 0 | 167 | 1 848 443 | 604 329 | 559 209 | 512 982 | 100 | 100 | 100 | 99.99 |
| 5/20/2023 | 51 | 0 | 0 | 51 | 1 848 494 | 604 329 | 559 209 | 513 033 | 100 | 100 | 100 | 100 |

^a^, The cumulative dose rate shown in % was calculated using the following formula: [(number of doses applied - cumulative/total number of doses for each category) * 100]; ^b^, the dose 2 category includes doses presented as a single dose. The number of administered vaccine doses was obtained from the National Health Data Network, Brazilian Ministry of Health, updated on 07/31/2024.

| **Supplementary Table 2:** Description of the distribution of doses of vaccines against coronavirus disease (COVID)-19 in the Quilombola population of Brazil according to Federation Units (States and Federal District) and vaccination coverage index (VCI) | | | | | | | | | | |
| --- | --- | --- | --- | --- | --- | --- | --- | --- | --- | --- |
| **Region** | **Dose 1** | **VCI^a^** | **Dose 2 and single dose** | **VCI^a^** | **Additional** | **Booster** | **2^o^ booster** | **Doses administered** | **VCI^a^** | **Total inhabitants^b^** |
| **Northeast** | 379 890 | 54.42 | 348 646 | 49.94 | 14 531 | 333 493 | 102 964 | 1 179 524 | 168.97 | 698 077 |
| Alagoas | 15 303 | 28.14 | 13 874 | 25.52 | 20 | 13 688 | 1938 | 44 823 | 82.43 | 54 374 |
| Bahia | 138 531 | 51.58 | 126 067 | 46.94 | 6705 | 106 742 | 41 466 | 419 511 | 156.20 | 268 573 |
| Ceará | 15 718 | 51.61 | 11 983 | 39.35 | 1185 | 13 225 | 5017 | 47 128 | 154.74 | 30 456 |
| Maranhão | 119 257 | 69.76 | 111 023 | 64.94 | 3544 | 116 104 | 29 376 | 379 304 | 221.87 | 170 961 |
| Paraíba | 8113 | 42.44 | 7638 | 39.95 | 10 | 7370 | 2213 | 25 344 | 132.57 | 19 117 |
| Pernambuco | 46 601 | 85.65 | 43 145 | 79.29 | 785 | 43 832 | 10 054 | 144 417 | 265.42 | 54 411 |
| Piauí | 16 293 | 38.56 | 15 554 | 36.81 | 212 | 15 701 | 8267 | 56 027 | 132.61 | 42 250 |
| Rio Grande do Norte | 8971 | 35.91 | 8944 | 35.80 | 1800 | 5901 | 791 | 26 407 | 105.71 | 24 980 |
| Sergipe | 11 103 | 33.69 | 10 418 | 31.61 | 270 | 10 930 | 3842 | 36 563 | 110.95 | 32 955 |
| **Southeast** | 97 497 | 56.68 | 91 005 | 52.90 | 2668 | 79 595 | 19 765 | 290 530 | 168.89 | 172 028 |
| Espírito Santo | 7450 | 46.58 | 7336 | 45.87 | 298 | 5122 | 276 | 20 482 | 128.07 | 15 993 |
| Minas Gerais | 71 729 | 54.83 | 66 550 | 50.87 | 2168 | 59 681 | 14 529 | 214 657 | 164.10 | 130 812 |
| Rio de Janeiro | 9705 | 65.32 | 8997 | 60.56 | 117 | 6644 | 2293 | 27 756 | 186.82 | 14 857 |
| São Paulo | 8613 | 83.09 | 8122 | 78.35 | 85 | 8148 | 2667 | 27 635 | 266.59 | 10 366 |
| **North** | 79 111 | 51.07 | 73 365 | 47.36 | 5163 | 62 536 | 12 716 | 232 891 | 150.34 | 154 911 |
| Acre | 0 | 0 | 7 | 0 | 1 | 5 | 4 | 17 | 0 | 0 |
| Amazonas | 1920 | 22.42 | 1800 | 21.02 | 743 | 955 | 889 | 6307 | 73.65 | 8563 |
| Amapá | 7067 | 82.01 | 6559 | 76.12 | 823 | 4000 | 1150 | 19 599 | 227.45 | 8617 |
| Pará | 64 228 | 49.49 | 59 468 | 45.83 | 3178 | 53 666 | 9960 | 190 500 | 146.80 | 129 770 |
| Rondônia | 706 | 50.07 | 540 | 38.30 | 11 | 384 | 136 | 1777 | 126.03 | 1410 |
| Roraima | 15 | 0 | 21 | 0 | 0 | 10 | 1 | 47 | 0 | 0 |
| Tocantins | 5175 | 79.00 | 4970 | 75.87 | 407 | 3 516 | 576 | 14 644 | 223.54 | 6551 |
| **Central-West** | 28 728 | 81.99 | 29 019 | 82.82 | 661 | 20 443 | 5912 | 84 763 | 241.92 | 35 037 |
| Federal District | 15 | 150.00 | 302 | 3020.00 | 11 | 587 | 257 | 1172 | 1 1720.00 | 10 |
| Goiás | 18 438 | 90.10 | 18 193 | 88.90 | 462 | 13 431 | 3785 | 54 309 | 265.38 | 20 465 |
| Mato Grosso do Sul | 2487 | 19.43 | 2539 | 19.83 | 33 | 1183 | 234 | 6476 | 50.59 | 12 802 |
| Mato Grosso | 7788 | 54.42 | 7985 | 453.69 | 155 | 5242 | 1636 | 22 806 | 1 295.80 | 1760 |
| **South** | 19 103 | 28.14 | 18 853 | 25.81 | 1014 | 16 966 | 4850 | 60 786 | 83.21 | 73 053 |
| Paraná | 379 890 | 51.58 | 4772 | 49.55 | 449 | 4290 | 1290 | 15 663 | 162.63 | 9631 |
| Rio Grande do Sul | 15 303 | 51.61 | 11 327 | 20.73 | 523 | 10 812 | 3169 | 37 358 | 68.38 | 54 631 |
| Santa Catarina | 138 531 | 69.76 | 2754 | 31.33 | 42 | 1864 | 391 | 7765 | 88.33 | 8791 |

^a^, the vaccination coverage index represents the number of doses of vaccines administered per 100 inhabitants. The index was calculated using the following formula: [(number of inhabitants/number of vaccine doses administered) * 100]; ^b^, the number of inhabitants and the number of vaccine doses administered were obtained from the National Health Data Network, Brazilian Ministry of Health, updated on 07/31/2024.

| **Supplementary Table 3:** Distribution of the Quilombola population by macro-region and Federative Unit of Brazil according to the Demographic Census of Brazil (2022) published by the Brazilian Institute of Geography and Statistics (IBGE of the Portuguese *Instituto Brasileiro de Geografia e Estatística*) and the National Health Data Network.* | | | |
| --- | --- | --- | --- |
| **Regions** | **IBGE (N)** | **National Network (N)** | **Difference (N, %)** |
| **Northeast** | 906 337 | 698 077 | 208 260 (22.98) |
| Alagoas | 37 724 | 54 374 | 16 650 (30.62) |
| Bahia | 397 502 | 268 573 | 128 929 (32.43) |
| Ceará | 23 994 | 30 456 | 6462 (21.22) |
| Maranhão | 269 168 | 170 961 | 98 207 (36.49) |
| Paraíba | 16 765 | 19 117 | 2352 (12.30) |
| Pernambuco | 78 864 | 54 411 | 24 453 (31.01) |
| Piauí | 31 786 | 42 250 | 10 464 (24.77) |
| Rio Grande do Norte | 22 371 | 24 980 | 2609 (10.44) |
| Sergipe | 28 163 | 32 955 | 4792 (14.54) |
| **Southeast** | 182 427 | 172 028 | 10 399 (5.70) |
| Espírito Santo | 15 659 | 15 993 | 334 (2.09) |
| Minas Gerais | 135 315 | 130 812 | 4503 (3.33) |
| Rio de Janeiro | 20 447 | 14 857 | 5590 (27.34) |
| São Paulo | 11 006 | 10 366 | 640 (5.82) |
| **North** | 167 311 | 154 911 | 12 400 (7.41) |
| Acre | 0 | 0 | 0 |
| Amazonas | 2812 | 8563 | 5751 (67.16) |
| Amapá | 12 894 | 8617 | 4277 (33.17) |
| Pará | 135 603 | 129 770 | 5833 (4.30) |
| Rondônia | 2925 | 1410 | 1515 (51.79) |
| Roraima | 0 | 0 | 0 |
| Tocantins | 13 077 | 6551 | 6526 (49.90) |
| **Central-West** | 44 997 | 35 037 | 9960 (22.13) |
| Federal District | 305 | 10 | 295 (96.72) |
| Goiás | 30 391 | 20 465 | 9926 (32.66) |
| Mato Grosso do Sul | 2572 | 1760 | 812 (31.57) |
| Mato Grosso | 11 729 | 12 802 | 1073 (8.38) |
| **South** | 29 114 | 73 053 | 43 939 (60.15) |
| Paraná | 7113 | 9631 | 2518 (26.14) |
| Rio Grande do Sul | 17 552 | 54 631 | 37 079 (67.87) |
| Santa Catarina | 4449 | 8791 | 4342 (49.39) |

*, the numerical and percentage differences in inhabitants between the two values of each database region were established; N, number of inhabitants; %, percentage. The number of Quilombola inhabitants was obtained from the National Health Data Network, Ministry of Health of Brazil, updated on 07/31/2024.

| **Supplementary Table 4:** Distribution of doses administered according to age in the Quilombola population and type of dose. | | | | | | |
| --- | --- | --- | --- | --- | --- | --- |
| **Age (years)^a^** | **Dose 1** | **Dose 2** | **Single dose** | **Booster** | **Additional** | **Vaccinated individuals** |
| 3 to 4 | 717 | 306 | 0 | 4 | 0 | 1027 |
| 5 to 11 | 10 758 | 8293 | 0 | 634 | 3 | 19 688 |
| 12 to 17 | 5818 | 4634 | 2 | 15 976 | 129 | 26 559 |
| 18 and 19 | 31 857 | 25 782 | 129 | 14 908 | 695 | 73 371 |
| 20 to 24 | 71 053 | 64 855 | 275 | 48 836 | 2544 | 187 563 |
| 25 to 29 | 67 123 | 62 114 | 276 | 49 294 | 2424 | 181 231 |
| 30 to 34 | 69 893 | 64 440 | 251 | 53 247 | 2677 | 190 508 |
| 35 to 39 | 71 083 | 66 411 | 247 | 57 388 | 2904 | 198 033 |
| 40 to 44 | 64 817 | 61 334 | 191 | 54 970 | 2905 | 184 217 |
| 45 to 49 | 56 257 | 53 300 | 129 | 50 010 | 2472 | 162 168 |
| 50 to 54 | 51 683 | 48 566 | 67 | 46 936 | 2405 | 149 657 |
| 55 to 59 | 45 935 | 43 747 | 63 | 44 722 | 2238 | 136 705 |
| 60 to 64 | 27 079 | 26 195 | 27 | 31 054 | 1304 | 85 659 |
| 65 to 69 | 17 419 | 16 938 | 11 | 21 901 | 756 | 57 025 |
| 70 to 74 | 7020 | 7005 | 2 | 11 551 | 344 | 25 922 |
| 75 to 79 | 2933 | 2960 | 5 | 5955 | 150 | 12 003 |
| +80 | 2265 | 2091 | 4 | 5639 | 87 | 10 086 |

^a^, age is presented in years and according to the open-access platform that contains information on the vaccines available in Brazil against coronavirus disease (COVID)-19 (https://infoms.saude.gov.br/). The number of doses of vaccines applied was obtained from the National Health Data Network, Ministry of Health of Brazil, updated on 07/31/2024.
